# Mentoring as a complex adaptive system – a systematic scoping review of prevailing mentoring theories in medical education

**DOI:** 10.1186/s12909-024-05707-5

**Published:** 2024-07-05

**Authors:** Mac Yu Kai Teo, Halah Ibrahim, Casper Keegan Ronggui Lin, Nur Amira Binte Abdul Hamid, Ranitha Govindasamy, Nagavalli Somasundaram, Crystal Lim, Jia Ling Goh, Yi Zhou, Kuang Teck Tay, Ryan Rui Song Ong, Vanessa Tan, Youru Toh, Anushka Pisupati, Vijayprasanth Raveendran, Keith Zi Yuan Chua, Elaine Li Ying Quah, Jeevasuba Sivakumar, Samyuktha Dhanalakshmi Senthilkumar, Keerthana Suresh, Wesley Teck Wee Loo, Ruth Si Man Wong, Yiying Pei, Julia Huina Sng, Simone Qian Min Quek, Jasmine Lerk Juan Owyong, Ting Ting Yeoh, Eng Koon Ong, Gillian Li Gek Phua, Stephen Mason, Ruaraidh Hill, Anupama Roy Chowdhury, Simon Yew Kuang Ong, Lalit Kumar Radha Krishna

**Affiliations:** 1 Yong Loo Lin School of Medicine, National University of Singapore, Level 11 NUHS Tower Block, 1E Kent Ridge Road, Singapore, 119228 Singapore; 2 Division of Supportive and Palliative Care, National Cancer Centre Singapore, 30 Hospital Boulevard, Singapore, 168583 Singapore; 3 Division of Cancer Education, National Cancer Centre Singapore, 30 Hospital Boulevard, Singapore, 168583 Singapore; 4 Department of Medical Sciences, Khalifa University College of Medicine and Health Sciences, Abu Dhabi, United Arab Emirates; 5 Department of Pharmacy, National Cancer Centre Singapore, 30 Hospital Boulevard, Singapore, 168583 Singapore; 6 Centre for Biomedical Ethics, National University of Singapore, Blk MD11, 10 Medical Drive, Singapore, #02-03, 117597 Singapore; 7 Duke-NUS Medical School, National University of Singapore, 8 College Road, Singapore, 169857 Singapore; 8 Division of Medical Oncology, National Cancer Centre Singapore, 30 Hospital Boulevard, Singapore, 168583 Singapore; 9 Medical Social Services, Singapore General Hospital, Block 3, Singapore, 169854 Singapore; 10 Assisi Hospice, 832 Thomson Road, Singapore, 574627 Singapore; 11 Lien Centre for Palliative Care, Duke-NUS Medical School, National University of Singapore, 8 College Road, Singapore, 169857 Singapore; 12 Palliative Care Institute Liverpool, Academic Palliative & End of Life Care Centre, Cancer Research Centre, University of Liverpool, 200 London Road, Liverpool, L3 9TA UK; 13 Health Data Science, University of Liverpool, Whelan Building The Quadrangle, Liverpool, Brownlow Hill, Liverpool, L69 3GB UK; 14 Department of Geriatric Medicine, Singapore General Hospital, Academia, Level 3, College Road, Singapore, 169608 Singapore; 15 PalC, The Palliative Care Centre for Excellence in Research and Education, PalC c/o Dover Park Hospice, 10 Jalan Tan Tock Seng, Singapore, 308436 Singapore

**Keywords:** Mentoring, Medical education, Complex adaptive systems, Host organization, Communities of practice, Mentorship theories

## Abstract

**Background:**

Effective mentorship is an important component of medical education with benefits to all stakeholders. In recent years, conceptualization of mentorship has gone beyond the traditional dyadic experienced mentor-novice mentee relationship to include group and peer mentoring. Existing theories of mentorship do not recognize mentoring’s personalized, evolving, goal-driven, and context-specific nature. Evidencing the limitations of traditional cause-and-effect concepts, the purpose of this review was to systematically search the literature to determine if mentoring can be viewed as a complex adaptive system (CAS).

**Methods:**

A systematic scoping review using Krishna’s Systematic Evidence-Based Approach was employed to study medical student and resident accounts of mentoring and CAS in general internal medicine and related subspecialties in articles published between 1 January 2000 and 31 December 2023 in PubMed, Embase, PsycINFO, ERIC, Google Scholar, and Scopus databases. The included articles underwent thematic and content analysis, with the themes identified and combined to create domains, which framed the discussion.

**Results:**

Of 5,704 abstracts reviewed, 134 full-text articles were evaluated, and 216 articles were included. The domains described how mentoring relationships and mentoring approaches embody characteristics of CAS and that mentorship often behaves as a community of practice (CoP). Mentoring’s CAS-like features are displayed through CoPs, with distinct boundaries, a spiral mentoring trajectory, and longitudinal mentoring support and assessment processes.

**Conclusion:**

Recognizing mentorship as a CAS demands the rethinking of the design, support, assessment, and oversight of mentorship and the role of mentors. Further study is required to better assess the mentoring process and to provide optimal training and support to mentors.

**Supplementary Information:**

The online version contains supplementary material available at 10.1186/s12909-024-05707-5.

## Background

Effective mentorship during medical training fosters professional development, personal growth, and ethical guidance [1–7]. For host institutions, established mentorship programs facilitate knowledge transfer, improve recruitment and retention, and contribute to a culture of continuous learning and growth, ultimately advancing the quality of healthcare delivery and research within the organization [1–5, 8, 9]. Yet, despite its importance, medical education still lacks a widely accepted operational definition of mentoring [10]. Mentorship is often conflated with advising or coaching. While advisors assist trainees in making informed academic decisions and coaches provide training and guidance to help trainees reach specific goals, mentorship is a bidirectional relationship whereby an experienced mentor provides personalized guidance and support to facilitate a mentee’s development [11]. In recent years, conceptualizations of mentorship have also evolved from this traditional dyadic experienced mentor and novice mentee relationship to peer and group mentoring formats and mentoring networks [11]. Recent reviews highlight the challenges related to mentoring and attribute multiple ethical issues, including bullying, coercion, misappropriation of mentee funding and resources, and publication parasitism to inadequate structuring, support, and oversight of mentorship programs [1, 12–19]. As accounts of ethical, legal, and professional issues related to mentoring continue to grow, the need for a common understanding and consistent approach to mentoring is evident [1, 18].

Current theories of mentoring struggle to contend with mentoring’s personalized, evolving, and reciprocal nature, which is often goal-sensitive and context-specific [20, 21]. Several authors have criticized conventional models that do not recognize the dynamic relationship between mentors and mentees and the influence of external factors [11, 12]. Some studies suggest that mentoring should be considered a complex adaptive system (CAS) [22–24]. With such a shift in thinking likely to change the design, support, and oversight of mentoring programs, we evaluate if mentoring displays the characteristics and functions of a CAS to address our primary research question *“does mentoring function as a complex adaptive system?”*

### Complex adaptive systems

Some authors propose that a CAS-led perspective better captures mentoring’s non-linear, diverse, individualized, and unpredictable interrelationships [25]. A CAS is a system composed of many interacting and interdependent components (agents), whereby one agent’s actions can change the context for the others [26]. Features of CAS include complexity, adaptation, non-linearity, and self-organization, resulting in the spontaneous emergence of new and unpredictable patterns, behaviors, and trajectories. We define these features throughout the manuscript and summarize the terms in Table 1 as characterized by Ellis et al. [27] and Gear et al. [28].

**Table 1:** Characteristics of a complex adaptive system (CAS) (as defined by Ellis et al. [27] and Gear et al. [28]

Core CAS elements	Features	Management principles
Multiple agents with schemata	Informal, collaborative networks of individuals or organizations that partner and contribute to solution making, each possessing their own beliefs, experiences, and expectations (schemata) The schemata held by each agent influence their perceptions, decisions, and actions within the mentorship system, contributing to its complexity and adaptability.	Respect democratic principles that lead to mutual adjustment; jointly steer courses of action
Self-organizing networks	The spontaneous emergence of new relationships, forms, or patterns of behavior arising from shared interests and goals and repeated agent interactions over time	Facilitate open and transparent lines of communication flow across the network, so that authority and legitimacy become vested in the process as a whole, not on the perspective of one agent
Co-evolution	An ongoing process in which agents are influenced by, and mutually adapt to, changes generated by agent interaction Innovative pathways of governance emerge – a variety of what is known as “emergent behavior” in CAS	Recognize and nurture the bidirectional and mutually beneficial nature of mentorship.
System adaptation	A system adjusts its structure, behavior, or function in response to changes in its environment or internal dynamics Involves the system’s ability to modify itself to maintain stability, optimize performance, or achieve objectives despite fluctuations or disturbances	Promote a culture of respect, continuous learning, and feedback.
Agent	A system element or part capable of responding to other agent actions and information Agents can including mentors, mentees, program coordinators, institutional leaders, and other stakeholders involved in the design, implementation, and evaluation of mentorship programs.	Foster a culture of collaboration and shared responsibility This principle emphasizes teamwork, cooperation, and collective accountability among all stakeholders involved in the mentorship process.
Non-linearity	A characteristic of agent interaction whereby small changes in one part of the system can lead to disproportionately large and unpredictable effects elsewhere, often resulting in emergent behaviors that are not directly proportional to the initial inputs	Embrace the unpredictability of mentorship dynamics and encourage mentors and mentees to be flexible and adaptable in their approaches, recognizing that small interactions can sometimes lead to major changes in personal or professional development and learning.
Feedback loops	Recursive mechanisms arising from multiple agent interactions that either amplify (positive) or dampen (negative) certain patterns or behaviors over time Positive feedback loops support a change trajectory while negative feedback loops tend to undermine or negate change.	Support mechanisms for regular and reciprocal feedback between mentors and mentees to foster a culture of continuous improvement.
Emergence	New system properties or complex patterns or behaviors are generated by interactions between the agents	Encourage openness and creativity to foster innovative ideas, perspectives, and solutions.
Boundaries	Artificial frames or socially constructed delineations or demarcations that define the scope, interactions, and relationships within the system that connect (not separate) a system with its environment. System fluidity means that boundaries cannot be defined objectively. Boundaries can be interpersonal, institutional, social, or conceptual, and often influence the flow of information and resources within the system and between the system and its environment.	Foster opportunities to transcend boundaries and collaborate across disciplines, professions, and organizational structures to promote innovation, inclusivity, and resilience in the mentorship system.
“Far-from-equilibirum”	A dynamic state in which complex systems maintain a stable appearance by balancing multiple interactions between diverse agents and feedback loops A state of dynamic interactions, where agents challenge existing norms and practices, and explore new possibilities for professional development and personal growth Stability can be disproportionately disrupted by small changes.	Recognize that small actions can have a large impact on the personal and professional development of others; strive to create positive change.
Path dependency	The influence of historical events, decisions, or behaviors on the current system’s behavior and trajectory Arises when past experiences or choices create constraints or biases that influence future events within the system	Recognize and critically evaluate institutional practices that may be influenced by historical factors and biases; proactively seek opportunities to promote diversity, inclusivity, and innovation in mentorship practices.

## Methods

### Theoretical lens

**Fig. 1:**
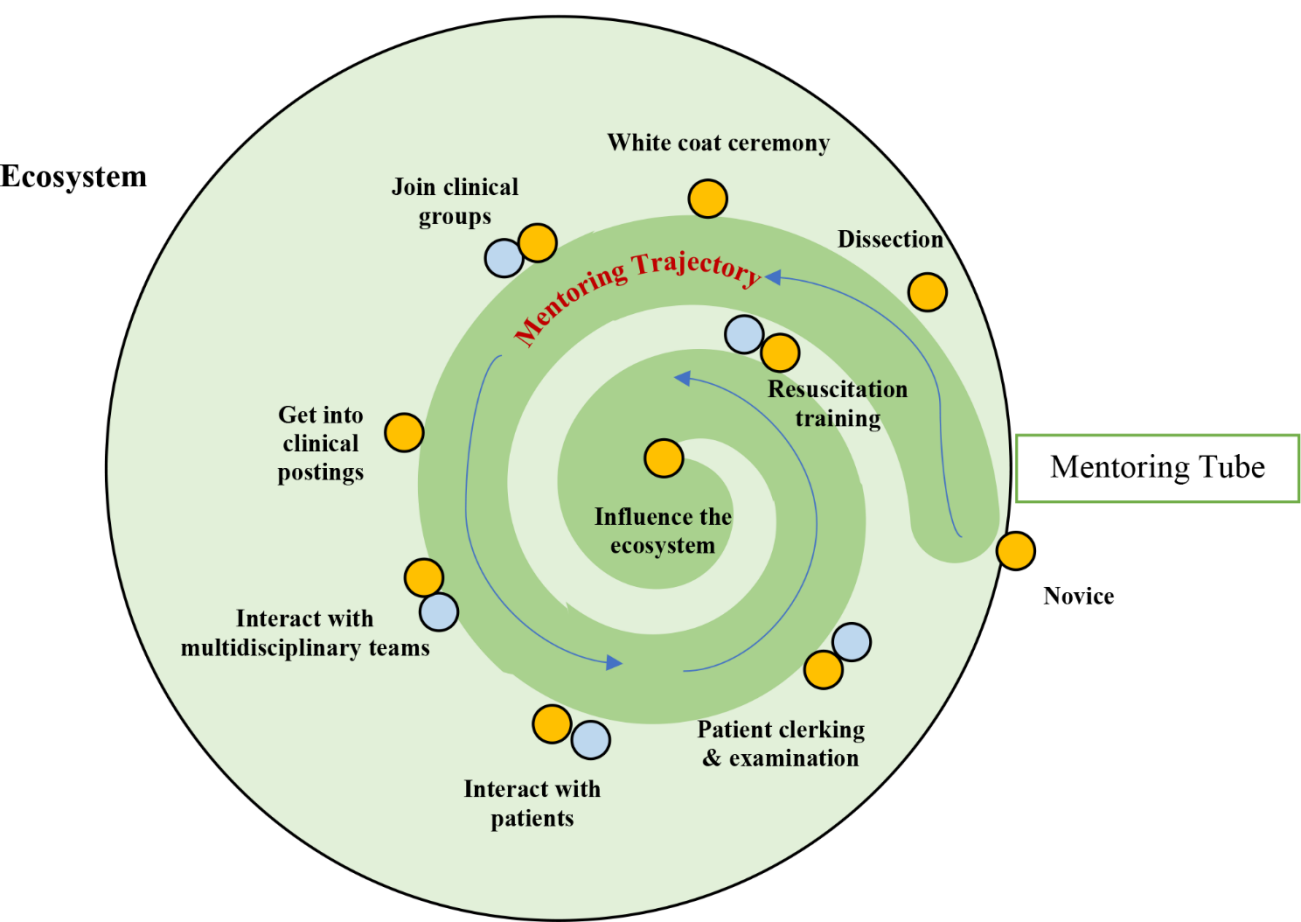
Mentoring ecosystem. The yellow circles represent the mentee’s microenvironment while the blue circles symbolize other stakeholders’ microenvironments. The dark green spiral represents the mentoring tube, and the thin blue lines represent the changing course of the mentoring relationship along the mentoring trajectory. The mentoring trajectory is framed around key stages of development. Some of these stages are highlighted. The mentoring trajectory is not depicted as a smooth course underlining the inevitable changes apparent across the mentoring stages

A mentoring ecosystem encompasses a broad range of mentors, mentees, and stakeholders, including institutions, all contributing to individual growth and development through mentorship. The concept of the mentoring ecosystem is like a Community of Practice, or a social network with mutual experiences and values [29], and is shaped around a predetermined course from marginal participation at the periphery of the mentoring program to a more central role within the mentoring program [29] (Fig. 1). This mentoring trajectory is framed on mentoring stages [30, 31], or clearly delineated phases of the mentoring process. Transitions from one stage to another create ideal assessment points, which in turn, inform the longitudinal mentoring support system, or mentoring umbrella. The mentoring umbrella is a framework where multiple forms of mentoring and support, including supervision, coaching, tutoring, instruction, and teaching, are provided to support an individual’s growth and development, like how an umbrella provides protection and coverage [32]. This approach ensures that mentees receive comprehensive support from different sources to enhance their learning, skill development, and career advancement [32]. The combination of the mentoring trajectory and mentoring umbrella creates the mentoring tube, which guides mentoring progress.

## SEBA review methodology

**Fig. 2:**
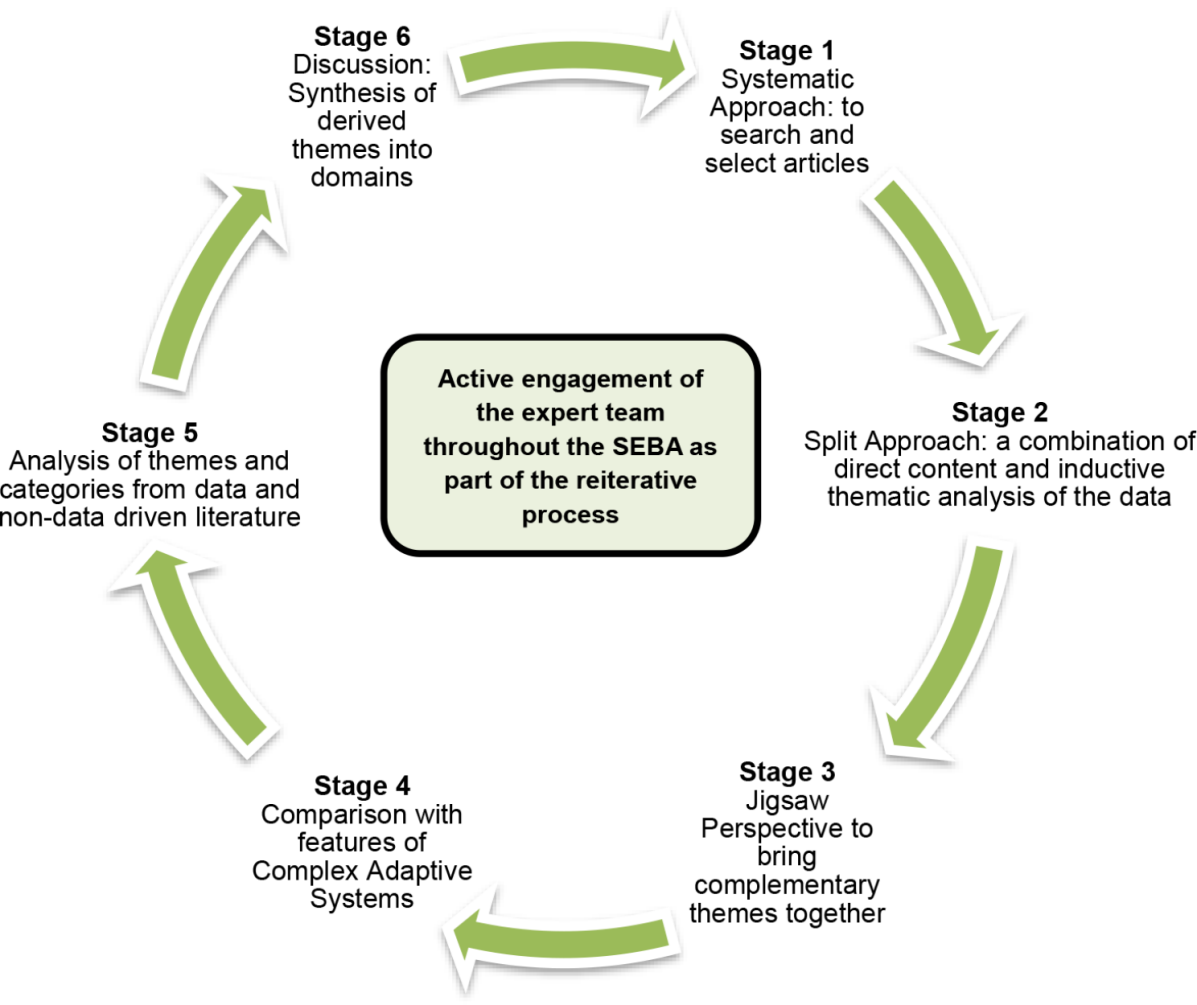
The SEBA process

Using Krishna’s Systematic Evidence-Based Approach (SEBA) to guide our scoping review [32–35], we explore mentoring in medical education as a sociocultural construct shaped by multiple stakeholder and host organization-related factors. This approach also accommodates the CAS lens through which we evaluate the different aspects of mentoring for features of CAS. SEBA is a methodologic framework for conducting systematic scoping reviews. The steps of the SEBA process involve: (1) a systematic approach whereby teams of medical education experts and researchers agree upon the research questions, search terms, and databases to be included; (2) the split approach in which a research team conducts inductive thematic analysis of the included articles allowing themes to emerge from the data while other research team(s) independently use a predefined set of codes to guide the analysis and identify themes; (3) the jigsaw perspective involves combining overlapping and complementary themes to create larger categories of themes; (4) a comparison process with the features of CAS ensures that relevant themes are not omitted; (5) analysis of data and non-data driven literature compares the themes derived from evidenced-based publications with those from non-data-based articles (editorials, grey literature, letters, opinion pieces, and perspectives) for similarity to ensure that the non-data-based articles do not bias the analysis; and (6) synthesis where the derived themes create the domains that inform the discussion (Fig. 2).

### Reflexivity

The research team consisted of medical students and research assistants, guided by Internal Medicine and Palliative Care consultants, with expertise in medical education, qualitative analysis, and conducting systematic reviews. The medical students were members of a peer-mentorship research program; their personal experiences influenced the study design and data interpretation. To provide a balanced review, an expert team comprising of a librarian from the National University of Singapore’s (NUS) Yong Loo Lin School of Medicine (YLLSoM) and local educational experts and clinicians at YLLSoM, National Cancer Centre Singapore, Palliative Care Institute Liverpool, and Duke-NUS Medical School guided the 6-stages of the SEBA process. The teams also engaged in personal and group reflexivity throughout the process to minimize the impact of personal experience bias.

### Stage 1: systematic approach

This SEBA-systematic scoping review is guided by the PRISMA-P checklist to ensure a reproducible and robust mapping of current notions of mentoring.

#### Identifying the research question

Guided by the expert team, the research team determined the primary research question to be: “does mentoring function as a complex adaptive system?” The secondary research question is: “what characteristics of CAS are evident in mentoring?”

#### Inclusion criteria

A population, concept, context (PCC) study design format was adopted to guide the research [36] (Table 2). We included all study types (quantitative, qualitative, mixed methods) and non-empirical manuscripts (perspectives, editorials, letters) involving medical students and medical trainees and physicians in Internal Medicine and its related subspecialties. We excluded studies from other disciplines and those involving mentorship by patients or interdisciplinary mentors, along with studies dealing with supervision, coaching, role-modeling, advising, or sponsorship. In keeping with Pham et al.’s [37] recommendations on sustaining the research process and accommodating existing manpower and time constraints, the research team restricted the searches to articles published between 1st January 2000 and 31st December 2023.

**Table 2:** Population, concept, context, inclusion and exclusion criteria applied to database search

PCC	Inclusion criteria	Exclusion criteria
**Population**	• Junior physicians, residents, and medical students in Internal Medicine and its specialities, as delineated by the American College of Physicians including Allergy and Immunology, Clinical Medicine, Community Medicine, Dermatology, General Practice, Geriatrics, Hospital Medicine, Neurology, Palliative Medicine, Cardiology, Endocrinology, Gastroenterology, Haematology, Immunology, Infectious Disease, Nephrology, Respiratory Medicine, and Rheumatology	• Clinical specialties not associated with Internal Medicine such as surgical specialties, Paediatrics, Emergency Medicine, Obstetrics and Gynaecology, and Clinical and Translational Science
**Concept**	• Accounts of mentoring • Theories of mentoring involving junior physicians, residents and/or medical students mentored by senior clinicians aimed at advancing the professional and/or personal development of the mentee. • The theories and accounts should include, but are not limited to explaining: o Mentoring processes o Mentoring relationship o Host organization o Outcomes of mentoring o Barriers to mentoring o Mentoring structure o Mentoring framework o Mentoring culture o Mentoring environment	• Mentoring for leadership, mentoring patients, or mentoring by patients, interdisciplinary mentoring • Supervision, coaching, role-modeling, advising, and sponsorship
**Context**	• Personal outcomes of mentoring • Professional development outcomes • Career related outcomes • Research and academia outcomes	• Studies where mentoring outcomes were not the main component evaluated

#### Database searching

Eleven members of the research teams searched five bibliographic databases (PubMed, Embase, PsycINFO, Cochrane Database of Systematic Reviews, CINAHL, Scopus) between 13 February 2023 and 20 April 2024 (Table 3). The research teams, each comprised of medical students and a senior reviewer, independently carried out the searches. The search terms and strategies used for database searching are detailed in Appendix 1.

**Table 3:** Agent-related factors

Individual	Structural and procedural factors impacting agent’s conduct, progress, and development
Unique norms, beliefs, values, principles and roles (*belief systems*) [7]	Regnant education approaches and the norms, skills and motivations set out by the team, the speciality, the institution and/or society, along with the program’s value, support and assessment systems (*desired characteristics*)
Working styles; opportunities [38, 39]; attitudes; emotions [40]; experience; skills; goals; demographic [40, 41]; socio-cultural [42–44]; ideological, contextual and psycho-emotional features (*narratives*)	The effects of the program’s formal curriculum; working hours; rules [45]; disciplinary consequences [46]; programs [47, 48]; attention to professional identity formation (PIF) [49–51]; administrative support [52]; faculty training and evaluation [52, 53] stage of training; access to personalized support and communication networks; hidden curriculum [50, 54–63]; prevailing discourses [58, 64–67]; daily activities [56, 68, 69]; and rites of passage [61, 63, 70–76] (*curricula*). Poor curricular design and support [77, 78] Overly hierarchal structure (176, 177) Formal and structured [78, 79] approach Poor contextualized learning [78, 80–84]
Psycho-emotional well-being and the adoption of reflective practice [85–87]; and personal coping strategies [88–94] including level of resilience [95, 96], and ability to cope with emotionally-rich experiences [97], failures [98], moments of crisis [64], disorienting experiences [99], and transitions [63, 98, 100–105] (*coping strategies*)	Differences in education approaches across different training sites; evolving expectations and stages of training; differences in support and assessment systems; and the program’s belief systems and shared identity (*host organization related facets*)
Personal, professional, ethical, psychosocial, emotional, cultural, organizational, societal, and legal spheres (*contextual considerations*)	Program’s learning objectives [106]; goals [107, 108]; timelines and professional standards [109, 110]; codes of conduct; expectations [111, 112]; implicit norms [113]; culture [114]; sociocultural norms and legal requirements [115–118] (henceforth *netiquette*);
Developing competencies; skills; knowledge; evolving goals; levels of engagement; judgment; decisions; and actions (*developing competencies*)	Situated practice [80, 81, 83, 84, 119–123] Poor contextualized learning [78, 80–84]
Stakeholder’s nature, quality, setting and progress of stakeholders’ interactions (*maturing relationship*).	Mentoring culture is contained within the mentoring ecosystem and is shaped by the program’s hidden curriculum [50, 54–63]; prevailing discourses [58, 64–67]; daily activities [56, 68, 69]; rites of passage [61, 63, 70–76], emotionally rich experiences [97]; failures [98]; moments of crisis [64]; disorienting experiences [99]; and transitions [63, 98, 100–105, 124]; as well as environmental, practical, clinical, administrative, cultural, social, and organizational, legal, ethical, and professional considerations.
Stakeholder’s reactions, mean-making, adaptation, and development; the importance they place on an interaction or specific incident; the stakeholder’s level of resilience and psycho-emotional status; and the available support to them [35, 125] (*meaning making and psycho-emotional state*)	Access to timely, individualised, context specific and appropriate role modelling; clinically relevant tutoring that caters to the mentee’s abilities, needs, goals and opportunities; supervised immersion into the clinical practice that accommodates the individual’s narratives, experiences, contextual considerations and goals; timely and comprehensive guided reflections; individualised, necessary, prompt, and constructive feedback; context specific advice; stage specific assessment led coaching; and longitudinal, personalised, appropriate, timely, and holistic mentored support (*mentoring umbrella*)
	The waxing and waning nature of the intensity of practice [50, 126–128] Flexible [78, 129, 130] approach that that caters to the apprentice’s personal needs [79, 131], abilities [121], changing contextual considerations [130, 132], quality of the apprenticeship relationship [77, 78, 81, 119, 128, 130, 132–136], and the learning environment [77, 79, 84, 132]. Time, practical and logistical limitations hinder apprenticeship [129, 137]
	Mentor selection including approachability [79, 81, 83, 126–128, 130, 132, 133, 135, 137–143], training and experience [77–79, 81, 129, 134, 135, 139, 144, 145], commitment to their training roles, openness to feedback [77, 80, 119, 141], and the tutor’s ability to role model, support and nurture clinical and professional attitudes, skills and conduct [50, 84, 126, 133, 138, 139, 146] Ineffective mentor [50, 78, 81, 84, 130, 132, 137, 147]

#### Extracting and charting the data

Each group independently reviewed the abstracts and titles and discussed their findings at online meetings where Sandelowski and Barroso [148]’s ‘negotiated consensual validation’ was used to achieve consensus on the final list of full-text articles to be reviewed. Data extracted from each manuscript meeting inclusion criteria included the author, year of publication, study type, study population, study location, components of the mentorship ecosystem, including mentoring approach/theories, stakeholders, mentoring structure and relationships, environment and external influences, and main findings of the study. The characteristics of all included studies are listed in Appendix 1.

### Stage 2: Split approach

Krishna’s ‘Split Approach’ [37–151] was employed to enhance the reliability of the data analyses. This approach involves the research team dividing (or ‘splitting’) into different groups to independently analyze the manuscripts. This concurrent analysis enables review of data from different perspectives while also aiming to reduce omission of new findings or negative reports. For this review, three groups of researchers independently analyzed the included articles. Using best practices, the first team summarized and tabulated the included full-text articles [152, 153] (Appendix 1). Concurrently, the second team analyzed the included articles using Braun and Clarke’s approach to thematic analysis [154]. A third team of researchers employed Hsieh and Shannon’s [155] approach to directed content analysis to analyze the included articles using pre-determined codes drawn from several published manuscripts on mentorship in medical education [7, 156, 157]. These studies were chosen because they provide the most recent review of mentoring practice at the time of this review [7] and offer the most recent longitudinal work on the subject [156, 157].

### Stage 3: Jigsaw perspective

In keeping with SEBA’s iterative process, the themes were reviewed by the expert and research teams. Overlaps between the themes were viewed as pieces of a jigsaw puzzle with the intention of combining overlapping or complementary pieces to create a bigger piece of the puzzle to form larger categories of themes.

### Stage 4: Comparison

Comparisons of the themes were made with the features of CAS identified by Ellis et al. [27] and Gear et al. [28], specifically multiple agents, self-organizing networks, co-evolution, system adaptation, non-linearity, feedback loops, emergence, boundaries, path dependency, and ‘far from equilibrium (Table 1). This step ensures that important themes were not omitted.

## Results

**Fig. 3:**
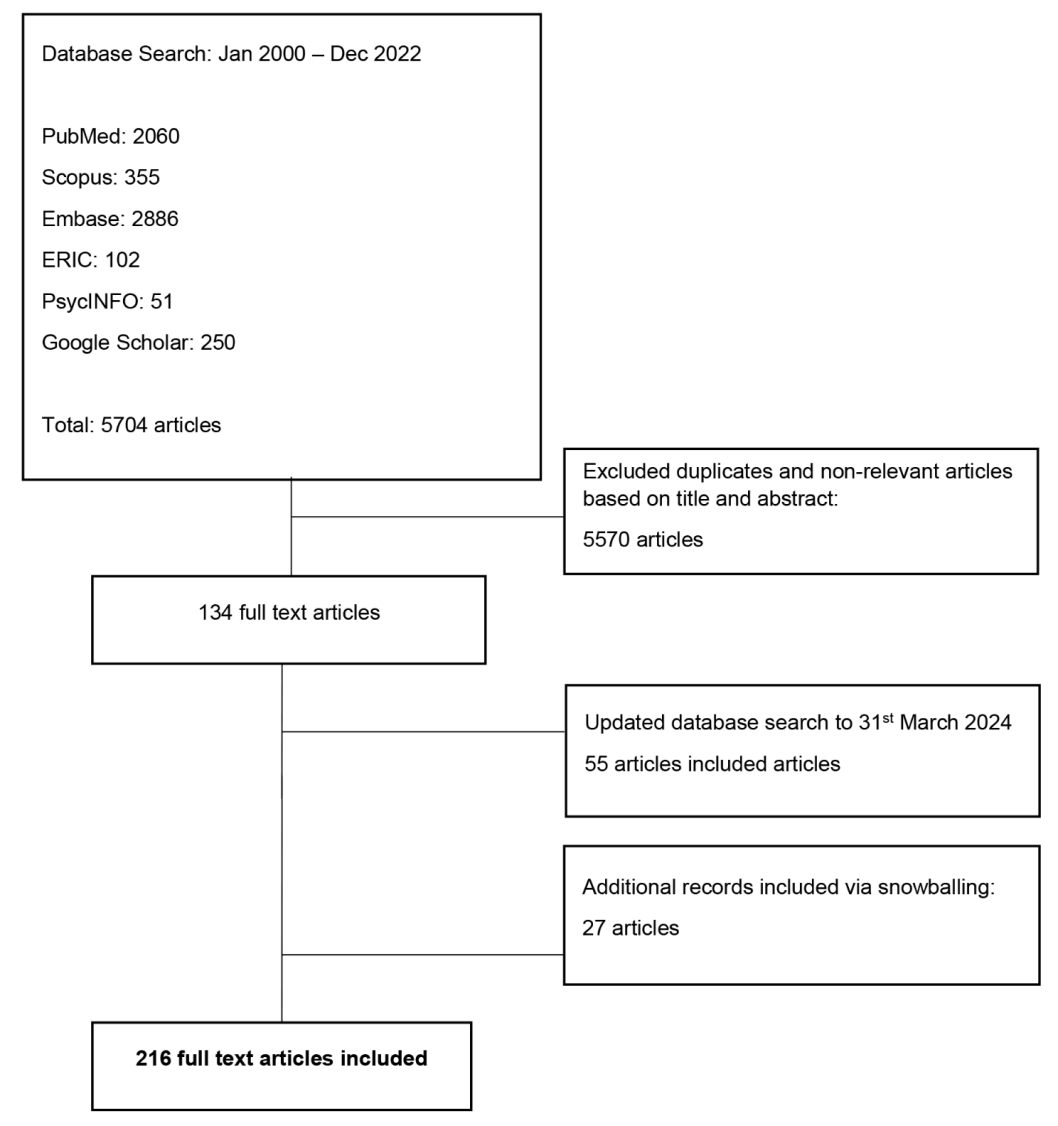
PRISMA flowchart. Snowballing articles were derived from searching the references of all included articles

The PRISMA diagram illustrates the process (Fig. 3). Of 5,704 abstracts reviewed, 134 full-text articles were evaluated, and 216 articles were included (additional articles included following snowballing, or review of the references of included articles). The themes elicited during thematic analysis of all 216 manuscripts were overlapped and combined (jigsaw approach) into larger categories of themes and compared with features of CAS to create 2 domains, namely mentoring relationships and mentoring programs, each with sub-themes as detailed below.

### Domain 1. Mentoring relationships

Mentoring relationships are influenced by the various stakeholders (agents) and the mentoring process.

#### Multiple stakeholders (agents)

A key feature of CAS is the presence of multiple agents interacting within collaborative networks [27, 28, 30, 158–160]. Our results support that mentors, mentees, institutional leaders, and multiple other stakeholders interact within the mentorship ecosystem by exchanging resources and information, thereby influencing each other’s perspectives and behaviors and collectively shaping the trajectory and outcomes of the mentorship dynamic. Several authors explored the roles of mentors, peer-mentors, mentees, and the host organization in mentoring programs and noted that the nature of collaborative networks can be a mix of formal and informal approaches [7, 161–166]. Even within formal programs [30, 167–170], with their clearly defined roles and responsibilities, expectations, and codes of conduct, the presence of multiple agents, each with their roles, goals, responsibilities, and areas of interest, suggests that interactions may veer toward the informal. Such variation draws attention shifting nature of the relationships between different stakeholders throughout a mentorship.

Similarly, stakeholder influence on mentees varies according to the circumstances and time. Some studies described fluidity in the nature of interactions between stakeholders, suggesting that membership in the mentoring relationship can change [7, 34, 157, 163]. New members may replace those who have completed their respective tasks, while other members may leave and re-enter the mentoring program at different points with different roles and responsibilities and varying levels of participation. Re-forming and adjusting mentoring relationships between new and returning stakeholders introduces more complexity. In addition, medical education’s hierarchical nature also impacts mentoring interactions and relationships, particularly when considering evolving circumstances and changing goals, expectations, and timelines.

The presence of multiple agents highlights the bilateral, but not necessarily equal, impact that mentoring relationships have on individual members. Stakeholder views and responses to their mentoring experiences are influenced by multiple factors, including their personal belief systems, developing competencies, coping strategies, psycho-emotional state, and maturing relationships with other stakeholders. Concurrently, the agent’s conduct, actions, and motivations are also influenced by contextual considerations, including changes to their professional roles and responsibilities, as well as stage-specific modifications to the curricula, host organization-related factors, mentoring culture, and access to support (Table 3).

#### Mentoring structures

The process, or structures (7, 49, 56, 60–63), of mentoring play a key role in shaping mentoring relationships [168, 171, 172]. We use Krishna et al.’s [6] concept of the mentoring ecosystem to illustrate the role that mentoring stages, mentoring trajectory, mentoring environment, mentoring umbrella, and the mentoring approach have on the CAS-related concepts of path dependency, boundaries, and adaptations in mentoring relationships [47].

##### Path dependency

Current concepts of path dependency [165, 173, 174] focus on the impact of past experiences or trajectories [7] on the current and future state of the system, suggesting that the cumulative effects of individual and programmatic experiences within the system have an enduring impact on its current structure and future potential [27, 28, 159]. Path dependency acknowledges that previous mentorship experiences [158, 175], historical choices [170] made by mentors and mentees, and the development of mentoring structures can shape the current dynamics and long-term possibilities of mentoring relationships. The impact of many of these effects is managed through the alignment of expectations [171] and available support.

##### Boundaries

Boundaries in CAS represent sociocultural constructs that highlight points of contact with other entities. Mentorship programs often span multiple levels of organization, including individual, interpersonal, institutional, and societal levels. These ‘fuzzy boundaries’ surrounding the micro-environments of each stakeholder [176] underscore the connections that influence the environment and adjacent micro-environments, adding another layer of complexity to the system and influencing the types of outcomes that can be achieved. The impact of these changes on the micro-environment depends on various factors, including the nature and duration of the mentoring relationship, the seniority, roles [166], and motivations of the stakeholders, the roles and expectations [170, 177] within the specific stages of mentoring, and the support provided by the mentoring umbrella [33, 35, 157, 178]. Moreover, the ‘fuzzy boundaries’ also enable the mentoring umbrella to shape micro-environments by providing timely and appropriate feedback, support, and remediation along the mentoring trajectory.

##### System adaptation

System adaptation refers to a system’s ability to modify itself to maintain stability, optimize performance, or achieve objectives despite disturbances [27, 28]. Within CAS, adaptations [179] are made to avoid major changes [180] to the system. Here, adaptability hinges on finding a balance between flexibility and consistency [7], focusing on making the smallest possible changes to the least significant elements to facilitate meaningful changes within the evolving micro-environments along the mentoring trajectory. In the mentoring ecosystem [181], the aim is to prioritize changes at the individual level and among a select few stakeholders [158, 176] to preserve stability in the broader program [182] while nurturing the mentorship process.

##### Co-evolution

Within CAS, interactions between agents give rise to mutual transformations [171, 183, 184]. As mentees adapt to the different goals, roles, and situated learning requirements across distinct mentoring stages along the mentoring trajectory, their interactions with other stakeholders [163] lead to changes in these stakeholders. This co-evolution is focused on preserving the quality of interactions within their mentoring relationship, referred to as mentoring dynamics. These dynamics are pivotal in shaping the personalized and enduring mentoring relationships that underpin mentoring success [6, 185, 186]. For example, as a mentee gains confidence and skills through effective feedback, the mentor can gain valuable insights to improve communication and feedback practices.

##### Feedback loops

Reflections on new mentoring experiences [171, 180], adaptations, and co-evolutions serve to reinforce positive changes while mitigating the repercussions of negative changes. This recursive influence of feedback loops also extends to thought processes, decision-making, and future actions [33, 35, 157, 168, 178, 187].

##### Emergence

The processes of adaptation [176], co-evolution, and feedback loops [163] give rise to the concept of ‘emergent behavior,’ behavior that emerges from interactions within the system, often focused on sustaining specific goals [27, 28]. ‘Emergent behaviour’ is shaped by the prevailing conditions, available resources and options, guidance received, and stakeholder interpretation of unfolding events.

##### Self-organization

When individuals experience shifts in their attitudes, thinking [32, 188], practice, and belief systems in response to ongoing changes, feedback, and environmental shifts, self-organization occurs. The mentoring framework, development of competencies [189], coping strategies, meaning-making process, and psycho-emotional state of individuals are pivotal in shaping self-organization within their micro-environment. These transformations in thoughts, emotions, and practices are guided by regnant standards [1, 7, 151, 190–201].

Self-organization subsequently influences the mentoring trajectory. When these changes align with mentoring objectives and approach, and are consistent with the overall trajectory, they facilitate goal attainment. However, if the trajectory deviates from alignment with these elements, mentees may struggle to reach their goals.

##### Non-linearity

This evolving membership [158, 171, 180, 202] of mentoring programs set within a hierarchical environment characterized by differences in diversity [174, 203–206], gender [206, 207], seniority, roles, and responsibilities [7, 163], coupled with adaptations, co-evolution, and the emergence of new behaviours, lead to non-linear responses [32, 164, 170] in interactions among stakeholders with diverse roles and responsibilities. This non-linearity [156, 159, 163, 208] is also apparent in the way individuals respond to various stimuli [1, 18, 33, 35, 95–210].

##### Far from equilibrium

The evolving processes [173] of adaptations [211], co-evolution, emergent behaviour, self-organization, non-linearity, and the influence of feedback loops [163, 187] expose a system in a state ‘far from equilibrium’ [27, 28], highlighted further during the COVID-19 pandemic [158, 212, 213] .In this context, even minor changes can lead to disproportionate impacts on mentoring relationships and processes [1, 7, 18, 21, 29, 35, 95, 156, 198, 210, 214–221]. In mentorship, being ‘far from equilibrium’ can also suggest a state of continuous learning, growth, and innovation, where mentor and mentees interactions are dynamic, challenging existing norms and practices and creating new possibilities for personal and professional development.

### Domain 2. Mentoring programs

Mentoring programs [30, 169, 179, 210, 212, 222–227] are often integrated [188, 208, 228] within the formal curriculum [7] and subject to oversight [176, 229] and evaluation by the host organization [53, 156, 230]. The increased emphasis on oversight within mentorship has grown amidst mounting concerns about ethical issues in mentoring [1, 12, 14–19]. While mentoring programs allow for a degree of flexibility, adaptability, and responsiveness, these functions are constrained by host organization-related factors and standards [231]. There are concerted efforts to instil consistency into practice, as evident in the structuring of the mentoring trajectory through the mentoring framework, the personalization of mentoring experiences, support, and assessments [189], and the establishment of clear transition points from one mentoring stage to the next, ensuring that the required knowledge, skills [232], and competencies for progression have been acquired. Furthermore, there is an emphasis on establishing clear expectations [233, 234] for the roles and responsibilities of stakeholders at each stage, particularly in light of their differing roles along the mentoring trajectory and maintaining clear standards for their engagement [202] as some stakeholders move in and out of various mentoring stages.

Moreover, mentoring programs can also establish clear areas of interest, goals, expectations, timelines, and entry criteria [210]. The mentoring project, setting, structure, and the faculty involved also help distinguish the mentoring process from other mentoring projects [38]. For example, the Palliative Medicine Initiative (PMI), as described by Krishna and colleagues, represents a structured research mentoring program jointly established within the Division of Supportive and Palliative Care and the Centre for Biomedical Ethics at the Yong Loo Lin School of Medicine at the University of Singapore. Comprised mainly of palliative care physicians and ethicists as mentors, the PMI is framed as a unique research-oriented mentoring program, with a specific focus on ethics and palliative care research.

Several authors frame mentoring projects in medical education as a community of practice (CoP). In CoPs, learning is a collaborative and social process. The unique nature of each mentoring project, with its specific inclusion and exclusion criteria [188], focal points, approaches, and distinct project boundaries underlie the notion that each project functions as a CoP. As most programs host more than one mentoring project, the mentoring program can be viewed as a landscape of practice (LoP), a complex tapestry of various CoPs [199, 235–240]. This view of mentoring programs as LoPs is supported by recent studies [156, 157], which reveal complexities within the mentoring process that arise when members of the host organization, mentors, or peer mentors engage in more than one project or CoP simultaneously, leading to a situation in which events or adaptations in one project can not only affect stakeholders in other CoPs but, perhaps more importantly, can also impact the nature and quality of their mentoring relationships in other projects within the LoP [156].

### Stage 5: analysis of evidence-based and non-data driven literature

Evidenced-based data from bibliographic databases were separated from grey literature and opinion, perspectives, editorials, letters, and non-data-based articles drawn from bibliographic databases and both groups were thematically analysed separately. The themes from both groups were compared to determine if there were additional themes in the non-data-driven group that could influence the narrative. There was consensus from the research team that themes from non-data-driven and peer-reviewed evidence-based publications were similar and did not bias the analysis.

## Discussion

### Stage 6: synthesis

In answering the research question “what characteristics of CAS are evident in mentoring?”, this review highlights how mentoring relationships involve multiple stakeholders and exhibit CAS-like features, such as emergence, adaptability, self-organization, co-evolution, non-linearity, path dependency, and a state far-from-equilibrium. These dynamics highlight the unpredictable and nonlinear nature of mentorship. However, traditional views of mentoring relationships impose rigid boundaries and predefined trajectories, akin to CoPs, which can stifle the natural evolution and complexity of mentoring relationships. The data challenge us to rethink how we define and approach mentorship, emphasizing the need to embrace its adaptability and self-organizing nature. They suggest that by acknowledging and leveraging the CAS-like characteristics of mentorship, we can foster more innovative and effective mentoring processes.

Our findings also emphasize that efforts to guide the mentoring process can occur at all stages of this journey. This is evident in how emergent behavior, adaptations, co-evolution, and self-organization are influenced by many host organization-related factors and are incorporated within professional codes of conduct, ethical standards, and organizational expectations. These features underscore the need for a more nuanced CAS-based theory to describe mentoring relationships and the factors that impact them. Adapting a resilience framework, or a model that emphasizes the capacity of systems to absorb disturbances, adapt to changing conditions, and maintain core functions [241], for example, to the context of CAS and mentorship in medical education can provide a different perspective on the dynamic and non-linear nature of mentorship relationships and how they can be influenced by factors such as feedback loops, emergence, self-organization, and adaptation. Ultimately, this can promote more supportive and sustainable approaches to mentoring to better address the diverse and evolving needs of mentees.

Our findings have several curricular and policy implications. First, given the multiple agents involved and the unpredictable nature of mentoring relationships [27, 28], mentoring programs should be embedded within a formal framework. This structure allows the host organization to establish clear guidelines and align expectations, timelines, and support. Moreover, it promotes consensus on the mentoring approach, structure, trajectory, and stages. Within this formal structure, accessible, longitudinal training opportunities should be established. Communication, assessment, and support systems for all stakeholders can also be put in place to create an environment that is conducive to effective mentoring, thereby minimizing path dependency, or the impact of historical decisions or biases [27, 28]. As mentorship relationships are non-linear whereby small changes can have a disproportionate impact on the mentee’s development and career trajectory [27, 28], the host organization should take an active role in supporting mentor training and conducting regular assessments of individual projects and the program as a whole. This is particularly important as mentoring is recognized as a proactive process that relies on the involvement of mentoring faculty. This also acknowledges co-evolution in mentorship and supports bidirectional growth and learning between mentors and mentees. Formal recognition of mentor contributions is warranted, along with the allocation of protected time from clinical service to ensure that mentors can effectively meet their mentoring obligations.

To mitigate the risks and biases of path dependency [27, 28], host organizations should evaluate institutional practices that may be influenced by historical factors and biases and conduct comprehensive, longitudinal assessments of the stakeholders, their mentoring relationships, progress, development, the program environment, structure, and approach. As part of this process, the use of mentoring diaries can provide a better understanding of mentee and mentor experiences over time and changing situations. Additionally, mentoring portfolios can provide multisource feedback and evidence of research, clinical, and professional development.

### Limitations

Analysis of literature on mentoring programs in medical schools is largely drawn from North American and European practices, possibly limiting generalizability to non-Western settings. We limited the search to studies involving medical students and residents in internal medicine and related sub-specialties. Mentoring experiences of surgical and other non-medical specialty residents may be different. While introducing perspective data gives insights into the initiation and development of mentoring programs, selection or reporting bias may be introduced. Further, the applicability of the findings in other healthcare settings may be compromised by conflations of mentoring in clinical and non-clinical settings.

## Conclusion

The literature supports the resemblance between mentorship and complex adaptive systems, highlighting the dynamic, emergent, and nonlinear nature of mentoring relationships, while advocating for a paradigm shift towards more supportive and efficient mentorship practices in medical education. Further study of the environments and boundaries of mentoring relationships are needed to guide our evolving perspective of mentoring.

### Electronic supplementary material

Below is the link to the electronic supplementary material.

Supplementary Material 1

Supplementary Material 2

## Data Availability

The datasets supporting the conclusions of this article are included within the article and its additional files.

## Abbreviations

CAS: Complex Adaptive System
SEBA: Systematic Evidence-Based Approach
PCC: Population, Concept Context
PIF: Professional Identity Formation
CoP: Community of Practice
LoP: Landscape of Practice
