# Supplementary material for: Mentoring as a complex adaptive system – a systematic scoping review of prevailing mentoring theories in medical education
Source: BMC Med Educ. 2024 Jul 5;24:726. doi: 10.1186/s12909-024-05707-5 (PMC11225364; doi:10.1186/s12909-024-05707-5)
Supplement: Supplementary file 1 — Supplementary Material 1 [file 12909_2024_5707_MOESM1_ESM.docx]

**Appendix 1.** **Search Terms and Strategies Used for Database Searching**

| **PubMed** | (mentor[MeSH] OR mentoring[MeSH] OR mentors[MeSH] OR mentorship[MeSH] OR mentor[tiab] OR mentors[tiab] OR mentoring[tiab] OR mentorship[tiab] OR “mentoring structure”[tiab] OR “mentoring structures”[tiab] OR “mentoring relationship”[tiab] OR “mentoring relationships”[tiab] OR “mentoring environment”[tiab] OR “mentoring environments”[tiab]) AND (“Schools, Medical”[MeSH] OR “Medicine”[MeSH] OR “Students, Medical”[MeSH] OR “medical student”[tiab] OR “medical students”[tiab] OR “medical school”[tiab] OR “medical schools”[tiab] OR medicine[tiab])  AND  (theory[tiab] OR theories[tiab] OR concepts[tiab] OR concept[tiab] OR approach[tiab] OR approaches[tiab] OR principle[tiab] OR principles[tiab] OR basis[tiab]) |
| --- | --- |
| **Scopus** | ALL ( 'mentor' OR 'mentors' OR 'mentoring' OR 'mentorship' ) AND ( 'theory' OR 'concept' OR 'approach' OR 'principle' ) |
| **Embase** | (‘mentoring’/exp OR ‘mentor’/exp OR ‘mentor’:ti,ab OR ‘mentors’:ti,ab OR ‘mentoring’:ti,ab OR ‘mentorship’:ti,ab OR ‘mentoring structure’:ti,ab OR ‘mentoring structures’:ti,ab OR ‘mentoring relationship’:ti,ab OR ‘mentoring relationships’:ti,ab OR ‘mentoring environment’:ti,ab OR ‘mentoring environments’:ti,ab) AND (‘medical school’/exp OR ‘medicine’/exp OR ‘medical students’/exp OR ‘medical student’:ti,ab OR ‘medical students’:ti,ab OR ‘medical school’:ti,ab OR ‘medical schools’:ti,ab OR ‘medicine’:ti,ab)  AND  (‘theory’/exp OR ‘conceptual framework’/exp OR ‘theory’:ti,ab OR ‘theories’:ti,ab OR ‘concepts’:ti,ab OR ‘concept’:ti,ab OR ‘approach’:ti,ab OR ‘approaches’:ti,ab OR ‘principle’:ti,ab OR ‘principles’:ti,ab OR ‘basis’:ti,ab) |
| **PsycInfo** | (exp Mentor/ OR mentor.tw. OR mentors.tw. OR mentoring.tw. OR mentorship.tw. OR mentoring structure.tw. OR mentoring structures.tw. OR mentoring relationship.tw. OR mentoring relationships.tw. OR mentoring environment.tw. OR mentoring environments.tw.) AND (exp Medical students/ OR exp Medical education/ OR medical student.tw. OR medical students.tw. OR medical school.tw. OR medical schools.tw. OR medicine.tw.)  AND  (exp theory/ OR theories.tw OR concepts.tw) |
| **Google Scholar** | intitle:mentor* AND (intitle:medicine OR intitle:medical) AND intitle:theor* |
| **ERIC** | abstract:"mentor" AND "medicine" |
