# Supplementary material for: Mentoring as a complex adaptive system – a systematic scoping review of prevailing mentoring theories in medical education
Source: BMC Med Educ. 2024 Jul 5;24:726. doi: 10.1186/s12909-024-05707-5 (PMC11225364; doi:10.1186/s12909-024-05707-5)
Supplement: Supplementary file 2 — Supplementary Material 2 [file 12909_2024_5707_MOESM2_ESM.docx]

**Appendix 2. Tabulated Summaries of Included Articles**

|  | Author(s) | Title | Year | Journal | Summary |
| --- | --- | --- | --- | --- | --- |
| 1 | B. Ellis and S. Herbert | Complex adaptive systems (CAS): An overview of key elements, characteristics and application to management theory," | 2011 | Inform Prim Care | **Objective:** To identify key elements and characteristics of complex adaptive systems (CAS) relevant to implementing clinical governance, drawing on lessons from quality improvement programmes and the use of informatics in primary care.  **Method:** The research strategy includes a literature review to develop theoretical models of clinical governance of quality improvement in primary care organisations (PCOs) and a survey of PCOs.  **Results:** Complex adaptive system theories are a valuable tool to help make sense of natural phenomena, which include human responses to problem solving within the sampled PCOs. The research commenced with a survey; 76% (n16) of respondents preferred to support the implementation of clinical governance initiatives guided by outputs from general practice electronic health records. There was considerable variation in the way in which consultation data was captured, recorded and organised. Incentivised information sharing led to consensus on coding policies and models of data recording ahead of national contractual requirements. Informatics was acknowledged as a mechanism to link electronic health record outputs, quality improvement and resources. Investment in informatics was identified as a development priority in order to embed clinical governance principles in practice.  **Conclusions**: Complex adaptive system theory usefully describes evolutionary change processes, providing insight into how the origins of quality assurance were predicated on rational reductionism and linearity. New forms of governance do not neutralise previous models, but add further dimensions to them. Clinical governance models have moved from deterministic and 'objective' factors to incorporate cultural aspects with feedback about quality enabled by informatics. The socio-technical lessons highlighted should inform healthcare management. |
| 2 | C. Gear, E. Eppel, and J. Koziol-McLain, | Advancing Complexity Theory as a Qualitative Research Methodology | 2018 | Int J Qual Methods | Although complexity theory is increasingly used to explain and understand complex health-system behavior, little is known about utilizing complexity theory to augment qualitative research methods. We advance this field by describing our use of complexity theory as a qualitative research methodology to explore sustainable health-care responses to intimate partner violence. We outline how complexity theory shaped our theoretical perspective, conceptualization of the research problem, and selection of methodology and methods. We show how a research methodology informed by complexity theory can capture new insights into complex problems, advancing the application of complexity theory and qualitative research design. |
| 3 | L.K.R. Krishna, A. Pisupati, Y.T. Ong, K.J.H. Teo, M.Y.K. Teo, V. Venktaramana, et al. | Assessing the effects of a mentoring program on professional identity formation | 2023 | BMC Med Educ | **Background**: Medical education has enjoyed mixed fortunes nurturing professional identity formation (PIF), or how medical students think, feel and act as physicians. New data suggests that structured mentoring programs like the Palliative Medicine Initiative (PMI) may offer a means of developing PIF in a consistent manner. To better understand how a well-established structured research mentoring program shapes PIF, a study of the experiences of PMI mentees is proposed.  **Methodology:** Acknowledging PIF as a sociocultural construct, a Constructivist approach and Relativist lens were adopted for this study. In the absence of an effective tool, the Ring Theory of Personhood (RToP) and Krishna-Pisupati Model (KPM) model were used to direct this dual Systematic Evidence-Based Approach (Dual-SEBA) study in designing, employing and analysing semi-structured interviews with PMI mentees and mentoring diaries. These served to capture changes in PIF over the course of the PMI’s mentoring stages. Transcripts of the interviews and mentoring diaries were concurrently analysed using content and thematic analysis. Complementary themes and categories identified from the Split Approach were combined using the Jigsaw Approach and subsequently compared with mentoring diaries in the Funnelling Process. The domains created framed the discussion.  **Results:** A total of 12 mentee interviews and 17 mentoring diaries were analysed, revealing two domains—PMI as a Community of Practice (CoP) and Identity Formation. The domains confirmed the centrality of a structured CoP capable of facilitating longitudinal mentoring support and supporting the Socialisation Process along the mentoring trajectory whilst cultivating personalised and enduring mentoring relationships.  **Conclusion:** The provision of a consistent mentoring approach and personalised, longitudinal mentoring support guided along the mentoring trajectory by structured mentoring assessments lay the foundations for more effective mentoring programs. The onus must now be on developing assessment tools, such as a KPM-based tool, to guide support and oversight of mentoring relationships. |
| 4 | L.K.R. Krishna, A. Pisupati, K.J.H. Teo, M.Y.K. Teo, C.W.N. Quek, K.Z.Y. Chua, et al. | Professional identity formation amongst peer-mentors in a research-based mentoring programme | 2023 | BMC Med Educ | **Background:** Mentoring plays a pivotal yet poorly understood role in shaping a physician’s professional identity formation (PIF) or how they see, feel and act as professionals. New theories posit that mentoring nurtures PIF by functioning as a community of practice through its structured approach and its support of a socialisation process made possible by its assessment-directed personalized support. To test this theory and reshape the design, employ and support of mentoring programs, we evaluate peer-mentor experiences within the Palliative Medicine Initiative’s structured research mentoring program.  **Methods:** Semi-structured interviews with peer mentors under the Palliative Medicine Initiative (PMI) at National Cancer Centre Singapore were conducted and triangulated against mentoring diaries to capture longitudinal data of their PMI experiences. The Systematic Evidence-Based Approach (SEBA) was adopted to enhance the trustworthiness of the data. SEBA employed concurrent content and thematic analysis of the data to ensure a comprehensive review. The Jigsaw Perspective merged complementary themes and categories identified to create themes/categories. The themes/categories were compared with prevailing studies on mentoring in the Funnelling Process to reaffirm their accuracy.  **Results:** Twelve peer-mentors participated in the interviews and eight peer-mentors completed the mentoring diaries. The domains identified were community of practice and identity work.  **Conclusions:** The PMI’s structured mentoring program functions as a community of practice supporting the socialisation process which shapes the peer-mentor’s belief system. Guided by a structured mentoring approach, stage-based assessments, and longitudinal mentoring and peer support, peer-mentors enhance their detection and evaluation of threats to their regnant belief system and adapt their self-concepts of identity and personhood to suit their context. These insights will help structure and support mentoring programs as they nurture PIF beyond Palliative Medicine. |
| 5 | K.T. Tay, X.H. Tan, L.H.E. Tan, D. Vythilingam, A.M.C. Chin, V. Loh, et al. | A systematic scoping review and thematic analysis of interprofessional mentoring in medicine from 2000 to 2019 | 2021 | J Interprof Care | Interprofessional mentoring in palliative care sees different members of the interprofessional team providing holistic, personalised and longitudinal mentoring support, skills training and knowledge transfer as they mentor trainees at different points along their mentoring journeys. However, gaps in practice and their risk of potential mentoring malpractice even as interprofessional mentoring use continues to grow in palliative medicine underlines the need for careful scrutiny of its characteristics and constituents in order to enhance the design, evaluation and oversight of interprofessional mentoring programmes. Hence, a systematic scoping review on prevailing accounts of interprofessional mentoring in medicine is con- ducted to address this gap. Using Arksey and O’Malley’s (2005) methodological framework for conducting scoping reviews and identical search strategies, 6 reviewers performed independent literature reviews of accounts of interprofessional mentoring published in 10 databases. Braun and Clarke’s (2006) thematic analysis approach was adopted to evaluate across different mentoring settings. A total of 11111 abstracts were identified from 10 databases, 103 full-text articles reviewed and 14 full-text articles were thematically analysed to reveal 4 themes: characterizing, implementing, evaluating and obstacles to interprofessional mentoring. Interprofessional mentoring is founded upon a respectful and collaborative mentoring relationship that thrives despite inevitable differences in individual values, ethical perspectives at different career stages within diverse working environments. This warrants effective mentor-mentee trainings, alignment of expectations, roles and responsibilities, goals and timelines, and effective oversight of the programmes. Drawing upon the data provided, an interprofessional mentoring framework is forwarded to guide the design, evaluation and oversight of the programmes. |
| 6 | V. Venktaramana, Y.T. Ong, J.W. Yeo, A. Pisupati, and L.K.R. Krishna | Understanding mentoring relationships between mentees, peer and senior mentors | 2023 | BMC Med Educ | **Background:** Mentoring relationships play a critical but poorly understood role in mentoring’s overall success. To overcome these knowledge gaps, a study of mentee experiences in the Palliative Medicine Initiative, a structured research-based mentoring program, is proposed. The program’s clearly described mentoring approach, competency-based mentoring stages and curated mentoring environment ensure a consistent mentoring experience. It provides a unique platform to study mentoring relationships longitudinally and its implications on professional identity formation.  **Methodology:** The Tool Design Systematic Evidence-Based Approach methodology is used to map and employ current understanding. A review of recent reviews on mentoring processes, mentoring’s effects, professional identity formation and professional identity formation assessment tools lay the foundation for the design of semi-structured interviews and mentoring diaries to evaluate the characteristics of successful mentoring relationships and mentoring’s impact on professional identity formation. The data accrued from these tools were evaluated using this methodology whilst changes in professional identity formation were assessed using the Ring Theory of Personhood.  **Results:** The semi-structured interviews revealed four themes: stakeholders, mentoring stages, mentoring relationships and professional identity formation whilst the mentoring diaries revealed two: mentoring processes and mentoring relationships. Two final domains emerged – mentoring relationships and professional identity formation.  **Conclusions**: The Palliative Medicine Initiative’s structured stage-based mentoring approach, trained stakeholders, curated environment, assessment-directed and personalized mentoring support reveal seven developmental stages of mentoring relationships. These culminate in changes to the values, beliefs and principles that shape how mentees see, feel and act as professionals. These findings suggest that mentoring programs may help to further develop and fine-tune their professional identity formation. |
| 7 | L.K.R. Krishna, L.H.E. Tan, Y.T. Ong, K.T. Tay, J.M. Hee, M. Chiam, et al. | Enhancing Mentoring in Palliative Care: An Evidence Based Mentoring Framework | 2020 | J Med Educ Curric Dev | **Background:** Growing concerns over ethical issues in mentoring in medicine and surgery have hindered efforts to reinitiate mentoring for Palliative Care (PC) physicians following the easing of COVID-19 restrictions. Ranging from the misappropriation of mentee's work to bullying, ethical issues in mentoring are attributed to poor understanding and structuring of mentoring programs, underlining the need for a consistent approach to mentoring practices.  **Methods:** Given diverse practices across different settings and the employ of various methodologies, a novel approach to narrative reviews (NR)s is proposed to summarize, interpret, and critique prevailing data on novice mentoring. To overcome prevailing concerns surrounding the reproducibility and transparency of narrative reviews, the Systematic Evidenced Based Approach (SEBA) adopts a structured approach to searching and summarizing the included articles and employed concurrent content and thematic analysis that was overseen by a team of experts.  **Results:** A total of 18 915 abstracts were reviewed, 62 full text articles evaluated and 41 articles included. Ten themes/categories were ascertained identified including Nature; Stakeholders; Relationship; Approach; Environment; Benefits; Barriers; Assessments; Theories and Definitions.  **Conclusion**: By compiling and scrutinizing prevailing practice it is possible to appreciate the notion of the mentoring ecosystem which sees each mentee, mentor, and host organization brings with them their own microenvironment that contains their respective goals, abilities, and contextual considerations. Built around competency based mentoring stages, it is possible to advance a flexible yet consistent novice mentoring framework. |
| 8 | M.V. Kavas, M. Demirören, A.M.A. Koşan, S.T. Karahan, and N.Y. Yalim | Turkish students’ perceptions of professionalism at the beginning and at the end of medical education: a cross-sectional qualitative study | 2015 | Med Educ Online | **Aim:** Medical students' perceptions of professionalism might reflect the impact of the current educational processes on their professional identity development. This study focuses on Ankara University Faculty of Medicine students' perceptions of 'good doctor' along with the factors effective on the formation of these perceptions.  **Method:** Six focus groups with 59 medical students from Grade-1 and Grade-6 were held. The transcripts of discussions were analyzed thematically.  **Results:** Results regarding 'being a good physician' mostly mirrored the findings of previous studies framing the medical professionalism concept. The thematic pattern of the discussions on the relation between professional development and medical education suggests that students suffer from a gradual erosion of perception during medical education. That the education cannot either change the person for the better or might downgrade the person instead of improving her/him were shared by participants from both grades. Students consider clinical practice and role models two main variables determining the person's qualification as a professional.  **Conclusions**: The formal and hidden programs determine the quality and efficacy of the professional education together. Attempts to restructure medical education must recognize the reciprocal dynamics between these two components and, thus, should carefully work out the practical aspect of the educational processes. |
| 9 | K.J.H. Teo, M.Y.K. Teo, A. Pisupati, R.S.R. Ong, C.K. Goh, C.H.X. Seah, et al. | Assessing professional identity formation (PIF) amongst medical students in Oncology and Palliative Medicine postings: a SEBA guided scoping review | 2022 | BMC Palliat Care | **Background:** Introduction to a multi-professional team who are working and caring for the dying, and facing complex moral and ethical dilemmas during Oncology and Palliative Medicine postings influence a medical student’s professional identity formation (PIF). However, limited appreciation of PIF, inadequate assessments and insufficient support jeopardise this opportunity to shape how medical students think, feel and act as future physicians. To address this gap, a systematic scoping review (SSR) of PIF assessment methods is proposed.  **Methods:** A Systematic Evidence-based Approach (SEBA) guided SSR of assessments of PIF in medical schools published between 1st January 2000 and 31st December 2021 in PubMed, Embase, ERIC and Scopus databases was carried out. Included articles were concurrently content and thematically analysed using SEBA’s Split Approach and the themes and categories identified were combined using SEBA’s Jigsaw Perspective. The review hinged on the following questions: “what is known about the assessment of professional identity formation amongst medical students?”, “what are the theories and principles guiding the assessment of professional identity formation amongst medical students?”, “what factors influence PIF in medical students?”, “what are the tools used to assess PIF in medical students?”, and “what considerations impact the implementation of PIF assessment tools amongst medical students?”.  **Results:** Two thousand four hundred thirty six abstracts were reviewed, 602 full-text articles were evaluated, and 88 articles were included. The 3 domains identified were 1) theories, 2) assessment, and 3) implementation in assessing PIF. Differing attention to the different aspects of the PIF process impairs evaluations, jeopardise timely and appropriate support of medical students and hinder effective implementation of PIF assessments.  **Conclusion:** The Krishna-Pisupati model combines current theories and concepts of PIF to provide a more holistic perspective of the PIF process. Under the aegis of this model, Palliative Care and Oncology postings are envisaged as Communities of Practice influencing self-concepts of personhood and identity and shaping how medical students see their roles and responsibilities as future physicians. These insights allow the forwarding of nine recommendations to improve assessments of PIF and shape the design of a PIF-specific tool that can direct timely and personalized support of medical students. |
| 10 | E.Y.H. Koh, K.K. Koh, Y. Renganathan, and L. Krishna | Role modelling in professional identity formation: a systematic scoping review | 2023 | BMC Med Educ | **Background:** Role modelling’s pivotal part in the nurturing of a physician’s professional identity remains poorly understood. To overcome these gaps, this review posits that as part of the mentoring spectrum, role modelling should be considered in tandem with mentoring, supervision, coaching, tutoring and advising. This provides a clinically relevant notion of role modelling whilst its effects upon a physician’s thinking, practice and conduct may be visualised using the Ring Theory of Personhood (RToP).  **Methods:** A Systematic Evidence Based Approach guided systematic scoping review was conducted on articles published between 1 January 2000 to 31 December 2021 in the PubMed, Scopus, Cochrane, and ERIC databases. This review focused on the experiences of medical students and physicians in training (learners) given their similar exposure to training environments and practices.  **Results**: 12,201 articles were identified, 271 articles were evaluated, and 145 articles were included. Concurrent independent thematic and content analysis revealed five domains: existing theories, definitions, indications, characteristics, and the impact of role modelling upon the four rings of the RToP. This highlights dissonance between the introduced and regnant beliefs and spotlights the influence of the learner’s narratives, cognitive base, clinical insight, contextual considerations and belief system on their ability to detect, address and adapt to role modelling experiences.  **Conclusion**: Role modelling’s ability to introduce and integrate beliefs, values and principles into a physician’s belief system underscores its effects upon professional identity formation. Yet, these effects depend on contextual, structural, cultural and organisational influences as well as tutor and learner characteristics and the nature of their learner-tutor relationship. The RToP allows appreciation of these variations on the efficacy of role modelling and may help direct personalised and longitudinal support for learners. |
| 11 | J. Y. Lim, S.Y.K. Ong, C.Y.H. Ng, K. L.E. Chan, S.Y.E.A. Wu, W.Z. So, et al | A systematic scoping review of reflective writing in medical education | 2023 | BMC Med Educ | **Background:** Reflective writing (RW) allows physicians to step back, review their thoughts, goals and actions and recognise how their perspectives, motives and emotions impact their conduct. RW also helps physicians consolidate their learning and boosts their professional and personal development. In the absence of a consistent approach and amidst growing threats to RW’s place in medical training, a review of theories of RW in medical education and a review to map regnant practices, programs and assessment methods are proposed.  **Methods:** A Systematic Evidence-Based Approach guided Systematic Scoping Review (SSR in SEBA) was adopted to guide and structure the two concurrent reviews. Independent searches were carried out on publications featured between 1st January 2000 and 30th June 2022 in PubMed, Embase, PsychINFO, CINAHL, ERIC, ASSIA, Scopus, Google Scholar, OpenGrey, GreyLit and ProQuest. The Split Approach saw the included articles analysed separately using thematic and content analysis. Like pieces of a jigsaw puzzle, the Jigsaw Perspective combined the themes and categories identified from both reviews. The Funnelling Process saw the themes/categories created compared with the tabulated summaries. The final domains which emerged structured the discussion that followed.  **Results:** A total of 33,076 abstracts were reviewed, 1826 full-text articles were appraised and 199 articles were included and analysed. The domains identified were theories and models, current methods, benefits and shortcomings, and recommendations.  **Conclusions:** This SSR in SEBA suggests that a structured approach to RW shapes the physician’s belief system, guides their practice and nurtures their professional identity formation. In advancing a theoretical concept of RW, this SSR in SEBA proffers new insight into the process of RW, and the need for longitudinal, personalised feedback and support. |
| 12 | L. Krishna, Y.P. Toh, S. Mason, and R. Kanesvaran | Mentoring stages: A study of undergraduate mentoring in palliative medicine in Singapore | 2019 | PLoS One | **Background:** Mentoring nurtures a mentee’s personal and professional development. Yet conflation of mentoring approaches and a failure to contend with mentoring’s nature makes it difficult to study mentoring processes and relationships. This study aims to understand of mentee experiences in the Palliative Medicine Initiative (PMI). The PMI uses a consistent mentoring approach amongst a homogeneous mentee population offers a unique opportunity to circumnavigate conflation of practices and the limitations posed by mentoring’s nature. The data will advance understanding of mentoring processes.  **Methods**: Sixteen mentees discussed their PMI experiences in individual face-to-face audio-recorded interviews. The two themes identified from thematic analysis of interview transcripts were the stages of mentoring and communication.  **Results:** The 6 stages of mentoring are the ‘pre-mentoring stage’, ‘initial research meetings’, ‘data gathering’, ‘review of initial findings, ‘manuscript preparation” and ‘reflections’. These sub- themes sketch the progression of mentees from being dependent on the mentor for support and guidance, to an independent learner with capacity and willingness to mentor others. Each subtheme is described as stages in the mentoring process (mentoring stages) given their association with a specific phase of the research process. Mentoring processes also pivot on effective communication which are influenced by the mentor’s characteristics and the nature of mentoring interactions.  **Conclusion:** Mentoring relationships evolve in stages to ensure particular competencies are met before mentees progress to the next part of their mentoring process. Progress is dependent upon effective communication and support from the mentor and appropriate and timely adaptations to the mentoring approach to meet the mentee’s needs and goals. Adaptations to the mentoring structure are informed by effective and holistic evaluation of the mentoring pro- cess and the mentor’s and mentee’s abilities, goals and situations. These findings underline the need to review and redesign the way assessments of the mentoring process are con- structed and how mentoring programs are structured. |
| 13 | J. Hee, Y.L. Toh, H.W. Yap, Y.P. Toh, R. Kanesvaran, S. Mason, et al. | The Development and Design of a Framework to Match Mentees and Mentors Through a Systematic Review and Thematic Analysis of Mentoring Programs Between 2000 and 2015 | 2020 | Mentor Tutoring: Partnersh Learn | The need for longitudinal and holistic support of medical students throughout their training has led to the development of formal mentoring programs. Whilst success of formal mentoring programs has been attributed to (a) pairing men- tees with trained and experienced mentors (matching), (b) the quality of mentoring interactions and (c) the presence of a nurturing environment, little attention has been attributed to establishing an effective matching process in medical school. We sought, in this review, to redress this gap within the context of matching medical students for mentoring. Modified PRISMA guidelines were followed to guide the review process. PubMed, ERIC, Cochrane Database, OVID and ScienceDirect databases were searched for articles on matching in medical schools and in allied health specialities published between 2000 and 2015. |
| 14 | J. M. Dancer | Mentoring in healthcare: theory in search of practice? | 2003 | Clin Manag | Mentoring has been a confused and often misunderstood activity, which has been introduced somewhat haphazardly to date in the healthcare sector. The general principles are outlined in an effort to clarify the mentoring process, and the Egan Skilled Helper Model is described as a suitable framework which can be adopted and adapted for a developmental style of mentoring applicable to healthcare professionals, both clinical and non-clinical. Examples of existing schemes are described, together with the issues, including benefits and disadvantages, which require further consideration. It is acknowledged that the benefits of the mentoring process are difficult to quantify, but it is to be hoped that improved understanding of the process, together with an appreciation of modern knowledge-based management theory, will lead to a greater concerted effort on the part of senior NHS management to provide an adequately co-ordinated, skilled and resourced mentoring service for all who would benefit. |
| 15 | E.T. Welsh, D. Bhave, and K. Yong Kim | Are you my mentor? Informal mentoring mutual identification | 2012 | Career Dev Int | **Purpose:** The purpose of this study is to understand the extent to which potential mentors and proteges agree that an informal mentoring relationship exists. Because these relationships are generally tacitly understood, either the mentor or protégé could perceive that there is a mentoring relationship when the other person does not agree. Whether gender affects this is also to be examined. Design/methodology/approach – Individuals were asked to identify their mentoring partners. Each report of a partner was then compared to the partner’s list to determine whether there was a match (i.e. both reported the relationship as an informal mentoring relationship) or a mismatch (i.e. where one partner reported the relationship as an informal mentoring relationship but the other did not). This pattern of matches and mismatches was then analyzed to determine level of matching and gender differences.  **Findings**: There is little agreement between mentoring partners: neither potential proteges nor potential mentors were very accurate at identifying reciprocal informal mentoring partners. However, gender was not found to be related to different levels of matching. |
| 16 | T.F. Reiss, J. Moss, T.R. Watkins, and A. Malhotra, | BEAR cage: mentoring through engagement | 2016 | Am J Respir Crit Care Med | **Abstract**: As health sciences is broadening its focus to include transforming new knowledge into various health care system improvements, establishing a career as a scientific investigator is increasingly a challenging process. To achieve success in this environment, young investigators need to integrate knowledge from many different disciplines. They also require understanding of both the art and science of research translation, as it enhances scientific creativity and efficient strategic thinking, leading to real-world innovations. Furthermore, this knowledge will better position young investigators for careers and leadership positions across university, industry, nonprofit, and government settings, including regulatory agencies such as the U.S. Food and Drug Administration and the European Medicines Agency. And it will improve their ability to compete for ever-tightening resources in these environments. Going forward, we must make every effort to find new venues to enhance and further develop this art and wisdom as a complementary discipline to the more traditional research skills. |
| 17 | T. Sonawane, R. Meshram, G. Jagia, R. Gajbhiye, and S. Adhikari | Effects of Mentoring in First Year Medical Undergraduate Students using DASS-21 | 2021 | J Clin Diagn Res | **Introduction**: Medical students often require high level of specialised institutional and personal support to facilitate success. Distress is commonly observed in medical undergraduate students which leads to poor academic performances. The stress though looks reasonable it needs to be addressed with right amount of counseling. A good mentoring session helps in reducing depression, stress and anxiety. Aim: To determine the effects of mentoring in first year medical students using Depression, Anxiety and Stress Scale - 21 Items (DASS 21).  **Materials and Methods:** It was an interventional study conducted in Seth GSMC, Department of Physiology, Mumbai, Maharashtra, India, from July 2019 to February 2020. Total 120, first-year medical undergraduates were recruited in the study. At the beginning, an orientation session was conducted for the faculty. Mentoring sessions were conducted for students once in a week. Data was collected using DASS-21 in the beginning in July 2019 to February 2019. Statistical analysis was done using Wilcoxon sign rank test. Five-point Likert scale was used for qualitative analysis of the feedback received from mentors as well as mentees. The p-value <0.05 was considered as significant.  **Results:** Out of 15 faculty members were eight were males and seven were females with mean age of 47±8 and 46±1 years, respectively. Among mentees, 64 were males and 56 were females with mean age of 17±8 and 17±6 years. A significant decrease was obtained in the levels of depression, anxiety and stress scores of students after mentoring. Wilcoxan sign rank sum test was used. The p-value before and after mentoring session was 0.00418 for Depression, 0.00033 for anxiety and 0.00805 for stress.  **Conclusion:** Mentoring was found to reduce stress, anxiety and depression in first-year medical undergraduate students. The mentoring program was found to be useful to students as well as faculty. It should be extended through all the years of under graduation. |
| 18 | M. Stamm and B. Buddeberg-Fische | The impact of mentoring during postgraduate training on doctors' career success | 2011 | Med Educ | **Objectives**: Although mentoring is perceived as key to a successful and satisfying career in medicine, there is a lack of methodologically sound studies to support this view. This study made use of a longitudinal design to investigate the impact of mentoring during postgraduate specialist training on the career success of doctors.  **Methods:** We analysed data pertaining to 326 doctors (172 women, 52.8%; 154 men, 47.2%) from a cohort of medical school graduates participating in the prospective SwissMedCareer Study, assessing personal characteristics, the possession of a mentor, mentoring support provided by the development network, and career success. The impact of personal characteristics on having a mentor was investigated using multiple linear regression analysis. The impacts of having a mentor and of having development network mentoring support on career success were analysed using hierarchical multiple regression analysis.  **Results:** Up to 50% of doctors reported having a mentor. A significant gender difference was found, with fewer female than male doctors having a mentor (40.7% versus 60.4% at the fifth assessment; p £ 0.001). Apart from gender, significant predictors of having a mentor were instrumentality (b = 0.24, p £ 0.01) and extraprofessional concerns (b =) 0.15, p £ 0.05). Both having a mentor and having career support from the development network were significant predictors of both objective (b = 0.15, p £ 0.01; b = 0.17, p £ 0.01) and subjective (b = 0.17, p £ 0.01; b = 0.14, p £ 0.05) career success, but not of career satisfaction.  **Conclusions**: This study confirmed the positive impact of mentoring on career success in a cohort of Swiss doctors in a longitudinal design. However, female doctors, who are mentored less frequently than male doctors, appear to be disadvantaged in this respect. Formal mentoring programmes could reduce barriers to mentorship and promote the career advancement of female doctors in particular. |
| 19 | B.R. Yehia, P.F. Cronholm, N. Wilson, S.C. Palmer, S.D. Sisson, C.E. Guilliames, et al. | Mentorship and pursuit of academic medicine careers: a mixed methods study of residents from diverse backgrounds | 2014 | BMC Med Educ | **Background**: Mentorship influences career planning, academic productivity, professional satisfaction, and most notably, the pursuit of academic medicine careers. Little is known about the role of mentoring in recruiting Black/ African American and Hispanic/Latino residents into academia. The objective of this study was to assess the influence of mentoring on academic medicine career choice among a cohort of racially and ethnically diverse residents.  **Methods:** A strategic convenience sample of U.S. residents attending national professional conferences between March and July 2010; residents completed a quantitative survey and a subset participated in focus groups.  **Results:** Of the 250 residents, 183 (73%) completed surveys and 48 participated in focus groups. Thirty-eight percent of residents were white, 31% Black/African American, 17% Asian/other, and 14% Hispanic/Latino. Most respondents (93%) reported that mentorship was important for entering academia, and 70% reported having sufficient mentorship to pursue academic careers. Three themes about mentorship emerged from focus groups: (1) qualities of successful mentorship models; (2) perceived benefits of mentorship; and (3) the value of racial/ethnic and gender concordance. Residents preferred mentors they selected rather than ones assigned to them, and expressed concern about faculty using checklists. Black/African American, Hispanic/Latino, and female residents described actively seeking out mentors of the same race/ethnicity and gender, but expressed difficulty finding such mentors. Lack of racial/ethnic concordance was perceived as an obstacle for minority mentees, requiring explanation of the context and nuances of their perspectives and situations to non-minority mentors.    **Conclusions**: The majority of residents in this study reported having access to mentors. However, data show that the lack of diverse faculty mentors may impede diverse residents’ satisfaction and benefit from mentorship relationships compared to white residents. These findings are important for residency programs striving to enhance resident mentorship and for institutions working to diversify their faculty and staff to achieve institutional excellence. |
| 20 | L.T. Eby, M.M. Butts, J. Durley, and B.R. Ragins, | Are bad experiences stronger than good ones in mentoring relationships? Evidence from the protégé and mentor perspective | 2010 | J Vocat Behav | Two studies examined the relative importance of good versus bad mentoring experiences in predicting subjective states associated with the mentoring relationship. Study 1 examined the protégé perspective and found general support for the proposition that, on average, bad is stronger than good in predicting protégé outcomes. Study 2 adopted the mentor perspective and found mixed support for the prediction that, on average, bad is stronger than good. The results are discussed in terms of advancing research and theory on the relational processes associated with mentoring in the workplace and the need to consider the relational context to more fully understand the relative predictive power of good and bad mentoring experiences. |
| 21 | M.S.F. Mohd Shafiaai, A. Kadirvelu, and N. Pamidi | Peer mentoring experience on becoming a good doctor: student perspectives | 2020 | BMC Med Educ | **Background:** PASS is a peer-led structured academic mentoring program designed to provide academic assistance for new students in their transition from college to university studies and also for students struggling in certain units. This study aims to establish acquired skills by peer leaders associated with peer-led mentoring via the PASS program, and to explore the role played by these acquired skills in their journey to become a successful doctor.  **Methods:** Study participants were forty selected second-year undergraduate medical students at Monash University Malaysia with commendable examination results. Validated pre-test and post-test questionnaires were administered to explore changes in the level of communication, leadership, professional, and pedagogical skills before and after participation in peer mentoring program. Qualitative analysis of focused group interviews was performed by an independent investigator to identify how the skills developed as a peer mentor may help with becoming a good doctor. Major themes were identified with the thematic-analysis approach.  **Results:** Thirty-eight students completed the pre-test and post-test questionnaires. Peer leaders reported improvement in oral and written skills for teaching; increased confidence to give constructive feedback; better stress management; efficient time management; improved interpersonal skills; and enhanced problem-solving and critical thinking capabilities. Eight major themes were identified from the interview and peer leaders reported positive experience of working in diverse environments and shouldering of responsibilities.  **Conclusions**: Peer-led mentoring provides a good opportunity for medical students to shoulder responsibilities as a leader and offers an experience of managing a team of their peers and juniors which in turn may enhance their communication, interpersonal, and leadership skills. |
| 22 | L.J. Morrison, E. Lorens, G. Bandiera, W.C. Liles, L. Lee, R. Hyland, et al. | Impact of a formal mentoring program on academic promotion of Department of Medicine faculty: A comparative study | 2014 | Med Teach | **Purpose:** To evaluate the impact of a formal mentoring program on time to academic promotion and differences in gender-based outcomes.  **Methods:** Comparisons of time to promotion (i) before and after implementation of a formal mentoring program and (ii) between mentored and non-mentored faculty matched for covariates. Using paired-samples t-testing and mixed repeated measures ANCOVA, we explored the effect of mentor assignment and influence of gender on time to promotion.  **Results**: Promotional data from 1988 to 2010 for 382 faculty members appointed before 2003 were compared with 229 faculty members appointed in 2003 or later. Faculty appointed in 2003 or later were promoted 1.2 years (mean) sooner versus those appointed before 2003 (3.7 [SD = 1.7] vs. 2.5 [SD = 2], p < 0.0001). Regardless of year of appointment, mentor assignment appears to be significantly associated with a reduction in time to promotion versus non-mentored (3.4 [SD = 2.4] vs. 4.4 [SD = 2.6], p = 0.011). Gender effects were statistically insignificant. Post hoc analyses of time to promotion suggested that observed differences are not attributable to temporal effects, but rather assignment to a mentor.  **Conclusions:** Mentoring was a powerful predictor of promotion, regardless of the year of appointment and likely benefited both genders equally. University resource allocation in support of mentoring appears to accelerate faculty advancement. |
| 23 | C.H. Ng, Z.H. Ong, J.W.H. Koh, R.Z.E. Ang, L.H.S. Tan, K.T. Tay, et al. | Enhancing Interprofessional Communications Training in Internal Medicine. Lessons Drawn From a Systematic Scoping Review From 2000 to 2018 | 2018 | J Contin Educ Health Pro | **Introduction:** Interprofessional communication (IPC) enhances patient experiences and outcomes and improves well-being and satisfaction among health care professionals. This scoping review seeks to guide design of IPC training in internal medicine.  **Methods**: The framework of Arksey and O'Malley (2005) guided this systematic scoping review in internal medicine across PubMed, Embase, CINAHL, Scopus, PsycINFO, ERIC, JSTOR, and Google Scholar databases for publications from the years 2000 to 2018.  **Results**: Twenty-two thousand eight hundred seventy-four abstracts were retrieved, 326 full-text articles were reviewed, and 32 articles were included. The themes identified using directed content analysis were indications for an IPC program, training stages, and obstacles. The rationale for IPC programs was to improve interprofessional teamwork and enhance patient care. IPC training occurs in five stages beginning with instilling the role, value, and skills behind IPC and gradually practicing these skills within the clinical setting. The challenges to IPC highlight the need to confront workplace hierarchies and the lack of resources.  **Discussion:** The findings of this systematic scoping review also serve to underscore the importance of understanding, evaluating, and influencing the clinical environment and the work environment and the need for new assessment tools that will guide the individualized, longitudinal, competency-based learning process that underpins IPC training. |
| 24 | S. Pinilla, T. Pander, P. von der Borch, M.R. Fischer, and K. Dimitriadis, | 5 years of experience with a large-scale mentoring program for medical students | 2015 | GMS Z Med Ausbild | **Abstract:** In this paper, we present our 5-year-experience with a large-scale mentoring program for undergraduate medical students at the Ludwig Maximilians-Universität Munich (LMU). We implemented a two-tiered program with a peer-mentoring concept for preclinical students and a 1:1-mentoring concept for clinical students aided by a fully automated online-based matching algorithm. Approximately 20-30% of each student cohort participates in our voluntary mentoring program. Defining ideal program evaluation strategies, recruiting mentors from beyond the academic environment and accounting for the mentoring network reality remain challenging. We conclude that a two-tiered program is well accepted by students and faculty. In addition, the online-based matching seems to be effective for large-scale mentoring programs. |
| 25 | J.H. Sng, Y. Pei, Y.P. Toh, T.Y. Peh, S.H. Neo, and L.K.R. Krishna | Mentoring relationships between senior physicians and junior doctors and/or medical students: A thematic review | 2017 | Med Teach | **Introduction:** Mentoring relationships are pivotal to the outcome of the mentoring process. This thematic review seeks to study the key aspects of mentoring relationships between senior physicians and junior doctors and/or medical students to inform efforts to improve mentoring programs.  **Methods:** Literature search was performed on publications across PubMed, ERIC, Cochrane Database of Systematic Reviews, OVID and ScienceDirect databases between 1 January 2000 and 31 December 2015 by three independent reviewers. The BEME guide and STORIES statement were used to develop a narrative from the articles selected.  **Results:** Thematic analysis of 49 articles reveals five semantic themes of initiation process, developmental process, evaluation process, sustaining mentoring relationship, and obstacles to effective mentoring. The evolving and relational-dependent nature of mentoring pivots upon the compatibility of mentors and mentees and the quality of their interactions, which in turn depend on mentoring environments and awareness of mentor-, mentee-, organizational-related factors and changes in context and goals.  **Conclusions**: Embrace of a consistent mentoring approach to ensure effective oversight of the mentoring process must be balanced with sufficient flexibility to ensure a mentee-centered approach. Efforts must be made to optimize the key aspects of mentoring relationships in order to ensure successful mentoring processes and outcomes. |
| 26 | C.W.S. Cheong, E. W.Y. Chia, K. T. Tay, W.J. Chua, F. Q.H. Lee, E.Y.H. Koh, et al. | A systematic scoping review of ethical issues in mentoring in internal medicine, family medicine and academic medicine | 2020 | Adv Health Sci Educ Theory Pract | Mentoring’s role in medical education is threatened by the potential abuse of mentoring relationships. Particularly affected are mentoring relationships between senior clinicians and junior doctors which lie at the heart of mentoring. To better understand and address these concerns, a systematic scoping review into prevailing accounts of ethical issues and professional lapses in mentoring is undertaken. Arksey and O’Malley’s methodological framework for conducting scoping reviews was employed to explore the scope of ethical concerns in mentoring in general medicine. Databases searched included Pub- Med, ScienceDirect, ERIC, Embase, Scopus, Mednar and OpenGrey. 3391 abstracts were identified from the initially search after removal of duplicates, 412 full-text articles were reviewed, 98 articles were included and thematically analysed. Unsatisfactory matching, misaligned expectations, inadequate mentor training, cursory codes of conduct, sketchy standards of practice, meagre oversight and unstructured processes have been identified as potential causes for ethical and professional breaches in mentoring practice. Changes in how professionalism is viewed suggest further studies of educational culture should also be carried out. The host organization plays a major role in establishing codes of conduct, expectations, and holistically, longitudinally oversight of the mentoring process and mentoring relationships. |
| 27 | S. Goh, R.S.M. Wong, E.L.Y. Quah, K.Z.Y. Chua, W.Q. Lim, A.D.R. Ng, et al. | Mentoring in palliative medicine in the time of COVID-19: a systematic scoping review | 2022 | BMC Med Educ | **Introduction:** The redeployment of mentors and restrictions on in-person face-to-face mentoring meetings during the COVID-19 pandemic has compromised mentoring efforts in Palliative Medicine (PM). Seeking to address these gaps, we evaluate the notion of a combined novice, peer-, near-peer and e-mentoring (CNEP) and interprofessional team-based mentoring (IPT) program.  **Methods:** A Systematic Evidence Based Approach (SEBA) guided systematic scoping review was carried out to study accounts of CNEP and IPT from articles published between 1st January 2000 and 28th February 2021. To enhance trustworthiness, concurrent thematic and content analysis of articles identified from structured database search using terms relating to interprofessional, virtual and peer or near-peer mentoring in medical education were employed to bring together the key elements within included articles.  **Results**: Fifteen thousand one hundred twenty-one abstracts were reviewed, 557 full text articles were evaluated, and 92 articles were included. Four themes and categories were identified and combined using the SEBA’s Jigsaw and Funnelling Process to reveal 4 domains - characteristics, mentoring stages, assessment methods, and host organizations. These domains suggest that CNEP’s structured virtual and near-peer mentoring process complement IPT’s accessible and non-hierarchical approach under the oversight of the host organizations to create a robust mentoring program.  **Conclusion:** This systematic scoping review forwards an evidence-based framework to guide a CNEP-IPT program. At the same time, more research into the training and assessment methods of mentors, near peers and mentees, the dynamics of mentoring interactions and the longitudinal support of the mentoring relationships and programs should be carried out. |
| 28 | D. Sambunjak, S. E. Straus, and A. Marusic | A systematic review of qualitative research on the meaning and characteristics of mentoring in academic medicine | 2010 | J Gen Intern Med | **Background:** Mentorship is perceived to play a significant role in the career development and productivity of academic clinicians, but little is known about the characteristics of mentorship. This knowledge would be useful for those developing mentorship programs.  **Objective:** To complete a systematic review of the qualitative literature to explore and summarize the development, perceptions and experiences of the mentoring relationship in academic medicine.  Date sources: Medline, PsycINFO, ERIC, Scopus and Current Contents databases from the earliest available date to December 2008.  **Review methods**: We included studies that used qualitative research methodology to explore the meaning and characteristics of mentoring in academic medicine. Two investigators independently assessed articles for relevance and study quality, and extracted data using standardized forms. No restrictions were placed on the language of articles.  **Results:** A total of 8,487 citations were identified, 114 full text articles were assessed, and 9 articles were selected for review. All studies were conducted in North America, and most focused on the initiation and cultivation phases of the mentoring relationship. Mentoring was described as a complex relationship based on mutual interests, both professional and personal. Mentees should take an active role in the formation and development of mentoring relationships. Good mentors should be sincere in their dealings with mentees, be able to listen actively and understand mentees' needs, and have a well-established position within the academic community. Some of the mentoring functions aim at the mentees' academic growth and others at personal growth. Barriers to mentoring and dysfunctional mentoring can be related to personal factors, relational difficulties and structural/institutional barriers.  **Conclusions:** Successful mentoring requires commitment and interpersonal skills of the mentor and mentee, but also a facilitating environment at academic medicine's institutions. |
| 29 | J.M. Hee, H.W. Yap, Z.X. Ong, S.Q.M. Quek, Y.P. Toh, S. Mason, et al. | Understanding the Mentoring Environment Through Thematic Analysis of the Learning Environment in Medical Education: a Systematic Review | 2019 | JJ Gen Intern Med | **Background:** Mentoring's success has been attributed to individualised matching, holistic mentoring relationships (MRs) and personalised mentoring environments (MEs). Whilst there is growing data on matching and MRs, a dearth of ME data has hindered development of mentoring programme. Inspired by studies likening MEs to learning environments (LEs) and data highlighting common characteristics between the two, this systematic review scrutinises reports on LEs to extrapolate the findings to the ME context to provide a better understanding of ME and their role in the mentoring process.  **Methods:** Using identical search strategies, 6 reviewers carried out independent literature reviews of LEs in clinical medicine published between 1 January 2000 and 31 December 2015 using PubMed, ERIC, Cochrane Database of Systematic Reviews, Google Scholar and Scopus databases. Braun and Clarke's (2006) approach to thematic analysis was adopted to circumnavigate LE's evolving, context-specific, goal-sensitive, learner-tutor relationally dependent nature.  **Results:** A total of 4574 abstracts were identified, 90 articles were reviewed, and 58 full-text articles were thematically analysed. The two themes identified were LE structure and LE culture. LE structure regards the framework that guides interactions within the LE. LE culture concerns the values and practices influencing learner-tutor-host organisation interactions.  **Discussion**: LE is the product of culture and structure that influence and are influenced by the tutor-learner-host organisation relationship. LE structure guides the evolving tutor-learner-host organisation relationship whilst the LE culture nurtures it and oversees the LE structure. Similarities between LEs and MEs allow LE data to inform programme designers of ME's role in mentoring's success. |
| 30 | E.W.Y. Chia, K.T. Tay, S. Xiao, Y.H. Teo, Y.T. Ong, M. Chiam, et al. | The Pivotal Role of Host Organizations in Enhancing Mentoring in Internal Medicine: A Scoping Review | 2020 | J Med Educ Curric Dev | In undergraduate and postgraduate medical education, mentoring offers personalized training and plays a key role in continuing medical education and the professional development of healthcare professionals. However, poor structuring of the mentoring process has been attributed to failings of the host organization and, as such, we have conducted a scoping review on the role of the host organization in mentoring programs. Guided by Levac et al's methodological framework and a combination of thematic and content analysis, this scoping review identifies their "defining" and secondary roles. Whilst the "defining" role of the host is to set standards, nurture, and oversee the mentoring processes and relationships, the secondary roles comprise of supporting patient care and specific responsibilities toward the mentee, mentor, program, and organization itself. Critically, striking a balance between structure and flexibility within the program is important to ensure consistency in the mentoring approach whilst accounting for the changing needs and goals of the mentees and mentors. |
| 31 | C.S. Kow, Y.H. Teo, Y.N. Teo, K.Z.Y. Chua, E.L.Y. Quah, N.H.B.A. Kamal, et al | A systematic scoping review of ethical issues in mentoring in medical schools | 2020 | BMC Med Educ | **Background:** Mentoring provides mentees and mentors with holistic support and research opportunities. Yet, the quality of this support has been called into question amidst suggestions that mentoring is prone to bullying and professional lapses. These concerns jeopardise mentoring’s role in medical schools and demand closer scrutiny.  **Methods**: To better understand prevailing concerns, a novel approach to systematic scoping reviews (SSR) s is proposed to map prevailing ethical issues in mentoring in an accountable and reproducible manner. Ten members of the research team carried out systematic and independent searches of PubMed, Embase, ERIC, ScienceDirect, Scopus, OpenGrey and Mednar databases. The individual researchers employed ‘negotiated consensual validation’ to determine the final list of articles to be analysed. The reviewers worked in three independent teams. One team summarised the included articles. The other teams employed independent thematic and content analysis respectively. The findings of the three approaches were compared. The themes from non-evidence based and grey literature were also compared with themes from research driven data.  **Results:** Four thousand six titles were reviewed and 51 full text articles were included. Findings from thematic and content analyses were similar and reflected the tabulated summaries. The themes/categories identified were ethical concerns, predisposing factors and possible solutions at the mentor and mentee, mentoring relationship and/or host organisation level. Ethical concerns were found to stem from issues such as power differentials and lack of motivation whilst predisposing factors comprised of the mentor’s lack of experience and personality conflicts. Possible solutions include better program oversight and the fostering of an effective mentoring environment.  **Conclusions**: This structured SSR found that ethical issues in mentoring occur as a result of inconducive mentoring environments. As such, further studies and systematic reviews of mentoring structures, cultures and remediation must follow so as to guide host organisations in their endeavour to improve mentoring in medical schools. |
| 32 | S.Y.S. Lim, E.Y.H. Koh, B.J.X. Tan, Y. P. Toh, S. Mason, and L.K.R. Krishna | Enhancing geriatric oncology training through a combination of novice mentoring and peer and near-peer mentoring: A thematic analysis of mentoring in medicine between 2000 and 2017 | 2020 | Geriatr Oncol | **Objectives**: Training in Geriatric Oncology is in crisis, facing increasing demands in the face of a growing population of older adults, a lack of trainers, and the need to adapt training to different settings and trainee needs. A combination of novice mentoring and near-peer and peer mentoring (C-NP mentoring) has been proposed to provide trainees with personalized training and additional support. This study proposes to evaluate the possibility of establishing a C-NP mentoring program in geriatric oncology, through extrapolation of data from well-established practices in Internal Medicine programs.  **Materials and Methods:** A systematic scoping review was carried out to provide scope of prevailing data and highlight the key processes behind effective C-NP mentoring programs. Six reviewers carried out independent literature searches on C-NP mentoring in medicine using Embase, ERIC, PubMed, and Scopus databases for articles published between 1st January 2000 and 31st December 2017. The Best Evidence Medical Education (BEME) collaboration guide and the STORIES (STructured apprOach to the Reporting In healthcare education of Evidence Synthesis) statement were used to develop a narrative from the thematic analysis of selected articles. Braun & Clarke (2006)’s approach to thematic analyses and Sambunjak et al.’s approach of “negotiated consensual validation” were then used to identify the final list of themes.  **Results:** 3913 citations were identified, 133 full-text articles were reviewed, and fifteen full-text articles were included. Thematic analysis was employed to circumnavigate mentoring's context-specific nature and identified ten semantic themes including the need, outcomes, obstacles, and improvements for C-NP mentoring, mentee and mentor participation and training, and matching and mentoring processes.  **Conclusion:** Data from this review allows the forwarding of the C-NP Mentoring Framework that will potentially enhance Geriatric Oncology training. The framework ensures a balance of consistency in recruitment, training, matching, pre-mentoring meetings, assessments processes, and flexibility to inculcate personalized aspects to the training and support. The C-NP Mentoring Framework will also enable effective oversight of the program and timely support of mentees in need. |
| 33 | B. Barron | Interest and self-sustained learning as catalysts of development: A learning ecology perspective | 2006 | Hum Dev | Adolescents often pursue learning opportunities both in and outside school once they become interested in a topic. In this paper, a learning ecology framework and an associated empirical research agenda are described. This framework highlights the need to better understand how learning outside school relates to learning within schools or other formal organizations, and how learning in school can lead to learning activities outside school. Three portraits of adolescent learners are shared to illustrate different pathways to interest development. Five types of self-initiated learning processes are identified across these case portraits. These include the seeking out of text based informational sources, the creation of new interactive activity contexts such as projects, the pursuit of structured learning opportunities such as courses, the exploration of media, and the development of mentoring or knowledge-sharing relationships. Implications for theories of human development and ideas for research are discussed. |
| 34 | R.L. Cruess, S.R. Cruess, and Y. Steinert | Medicine as a community of practice: implications for medical education | 2018 | Acad Med | The presence of a variety of independent learning theories makes it difficult for medical educators to construct a comprehensive theoretical framework for medical education, resulting in numerous and often unrelated curricular, instructional, and assessment practices. Linked with an understanding of identity formation, the concept of communities of practice could provide such a framework, emphasizing the social nature of learning. Individuals wish to join the community, moving from legitimate peripheral to full participation, acquiring the identity of community members and accepting the community's norms. Having communities of practice as the theoretical basis of medical education does not diminish the value of other learning theories. Communities of practice can serve as the foundational theory, and other theories can provide a theoretical basis for the multiple educational activities that take place within the community, thus helping create an integrated theoretical approach. Communities of practice can guide the development of interventions to make medical education more effective and can help both learners and educators better cope with medical education's complexity. An initial step is to acknowledge the potential of communities of practice as the foundational theory. Educational initiatives that could result from this approach include adding communities of practice to the cognitive base; actively engaging students in joining the community; creating a welcoming community; expanding the emphasis on explicitly addressing role modeling, mentoring, experiential learning, and reflection; providing faculty development to support the program; and recognizing the necessity to chart progress toward membership in the community. |
| 35 | L. Wesley, M. Ikbal, J. Wu, M. Wahab, and C. Yeam | Towards a practice guided evidence based theory of mentoring in palliative care | 2017 | J Palliat Care Med | Provision of end of life care and coping with the emotional and existential distress engendered by palliative care demands the provision of holistic support and training for palliativists. Mentoring is an effective means of meeting this need; however little is known of mentoring in palliative care and a universally accepted learning theory of mentoring remains lacking in this setting. To advance mentoring practice in palliative care, we review the only two evidenced based mentoring theories based upon narrative reviews of mentoring practice in the key specialties within palliative care teams. Building upon mentoring’s mentee, mentor and organizational dependent, goal specific, context sensitive features highlighted in both recent reviews of mentoring this paper proffers a working theory of mentoring. Constructed Krishna’s Mentoring Pyramid that underlines the 5 core elements of successful mentoring programs, we propose melding elements of the cognitive apprenticeship model with the adult learning theory using the multi-theories model of adult learning offers an effective starting point for a mentoring theory. More context-specific studies are needed to provide better insight into the validity of this framework in the ongoing pursuit of an interprofessional mentoring theory in Palliative Medicine. |
| 36 | M.J. Karcher, G.P. Kuperminc, S.G. Portwood, C.L. Sipe, and A.S. Taylor | Mentoring programs: A framework to inform program development, research, and evaluation | 2006 | J Community Psychol | As mentoring programs have proliferated, considerable variation in approaches to programmatic mentoring has emerged. Concomitant confusion exists about the context, structure, and goals that constitute mentoring as a distinct intervention. This article presents a brief summary of what is currently known about different approaches to mentoring and proposes a framework that identifies both the common and the specific elements among different youth mentoring approaches. Rather than focusing solely on the participants and contexts of mentoring programs, such as peer- or school-based mentoring, as the key elements that differentiate programs, the authors suggest that more fruitful program development and research will result from a closer examination of the context, structure, and goals of programs, as well as of three critical program elements: content, infrastructure, and dosage. To understand better how and under what conditions mentoring works, program developers and researchers should test hypotheses regarding the influences of these program elements based on theory-driven expectations about the interrelationships among proximal, enabling, and distal outcomes of mentoring programs. |
| 37 | J.N. Wells and C.S. Cagle | Preparation and participation of undergraduate students to inform culturally sensitive research | 2009 | Nurse Educ Today | This article provides insights into undergraduate students’ reflections on their learning from taking part in a student-staff collaborative study in their first year at a UK university. The study comprised a series of small-scale research and evaluation activities. In the individual narratives and jointly developed model presented in the article, the student authors identify a range of learning from their participation in this study and from co-authoring this article. |
| 38 | B. Bozeman and M.K. Feeney | Toward a useful theory of mentoring: A conceptual analysis and critique | 2007 | Admin Soc | In this review and critique of mentoring theory and research, the authors identify persistent problems in the development of mentoring theory. Their conceptual analysis highlights these problems with a “thought experiment” illustrating the inability of mentoring theory and research to resolve certain fundamental issues, the resolution of which is a prerequisite for the advancement of explanatory theory. They conclude with ideas about demarcating “mentoring” from the sometimes confounding concepts “training” or “socialization.” Absent an ability to distinguish mentoring from related activities, progress in explanatory theory will remain impeded. |
| 39 | Y.T. Ong, C.W.N. Quek, A. Pisupati, E.K.Y. Loh, V. Venktaramana, M. Chiam, et al. | Mentoring future mentors in undergraduate medical education | 2022 | PLoS One | **Background:** Efforts to support flagging mentoring programs facing shortages of experienced clinical mentors have had an unexpected and welcome effect. Supplementing traditional mentoring programs with peer-mentoring have not only addressed gaps in practice, structure, support and mentee oversight but have offered mentees charged with peer-mentoring duties the opportunity to take on mentoring roles under senior supervision. This study evaluates the experiences of peer-mentors within a local research mentoring program to better understand and advance this endeavor.    **Methods:** Semi-structured interviews and post-interview surveys based on recent reviews on mentoring were employed. Adapting the Systematic Evidence Based Approach, data was analysed using thematic and content analysis. Results were combined using the Jigsaw Perspective to ensure that key elements of the different mentoring stages were identified.    **Results:** The interviews and surveys revealed the following domains: Motivation, Initiation, Practicing, and Mentoring Environment.    **Conclusion****:** These findings provide novel insight into a structured framework that may help guide the experiences, training, assessment, and oversight of peer-mentors beyond the auspices of our local program. These general observations will equip host organizations with the direction they need to take in designing and executing peer-mentoring training and assessment programs of their own. Whilst the stages of peer-mentoring need further evaluation and an effective means of assessment and support pivotal, we believe our findings suggest that peer-mentoring may not only help to address the shortfall in mentors but is an invaluable learning experience that prepares and instils key values, beliefs and principles in young would-be mentors. |
| 40 | D. Indyk, D. Deen, A. Fornari, M.T. Santos, W.H. Lu, and L. Rucker, | The influence of longitudinal mentoring on medical student selection of primary care residencies | 2011 | BMC Med Educ | **Background:** The number of students selecting careers in primary care has declined by 41% in the last decade, resulting in anticipated shortages.  **Methods**: First-year medical students interested in primary care were paired with primary care mentors. Mentors were trained, and mentors and students participated in focus groups at the end of each academic year. Quantitative and qualitative results are presented.  **Results:** Students who remained in the mentoring program matched to primary care programs at 87.5% in the first year and 78.9% in the second year, compared to overall discipline-specific match rates of 55.8% and 35.9% respectively. Students reported a better understanding of primary care and appreciated a relationship with a mentor.  **Conclusions**: A longitudinal mentoring program can effectively support student interest in primary care if it focuses on the needs of the students and is supportive of the mentors. |
| 41 | S.N. Meeuwissen, R.E. Stalmeijer, and M. Govaerts | Multiple‐role mentoring: mentors’ conceptualisations, enactments and role conflicts | 2019 | Med Educ | **Introduction:** Outcome-based approaches to education and the inherent emphasis on programmatic assessment in particular, require models of mentoring in which mentors fulfil dual roles: coach and assessor. Fulfilling multiple roles could result in role confusion or even role conflicts, both of which may affect mentoring processes and outcomes. In this study, we explored how mentors conceptualise and enact their role in a multiple-role mentoring system and to what extent they experience role conflicts.  **Methods:** We conducted a constructivist grounded theory study at one undergraduate medical school. A purposive sample of 12 physician-mentors active in a programmatic assessment system was interviewed. Data analysis followed stages of open, axial and selective coding through which themes were constructed.  **Results:** Three predominant mentoring approaches were constructed: (i) empowering (a reflective and holistic approach to student development); (ii) checking (an observant approach to check whether formal requirements are met), and (iii) directing (an authoritative approach to guide students' professional development). Each approach encompassed a corresponding type of mentor-mentee relationship: (i) partnership; (ii) instrumental, and (iii) faculty-centred. Furthermore, mentors' strategies, focus, agency provided to students and perception of the assessment system characterised mentoring approaches and relationships. Role conflicts were mainly experienced by mentors with a directing mentoring approach. They used various coping mechanisms, including deviation from assessment guidelines.  **Conclusions:** In multiple-role mentoring in the context of programmatic assessment, mentors adopted certain predominant mentoring approaches, which were characterised by different strategies for mentoring and resulted in different mentor-mentee relationships. Multiple-role mentoring does not necessarily result in role conflict. Mentors who do experience role conflict seem to favour the directing approach, which is most at odds with key principles of competency-based education and programmatic assessment. These findings build upon existing mentoring literature and offer practical suggestions for faculty development regarding approaches to mentoring in programmatic assessment systems. |
| 42 | D. Rangachari, L.E. Brown, D.E. Kern, and M.T. Melia | Clinical coaching: Evolving the apprenticeship model for modern housestaff | 2017 | Med Teach | **Background:** Direct observation with feedback to learners should be a mainstay in resident education, yet it is infrequently done and its impact on consultation skills has rarely been assessed.  **Approach:** This project presents the framework and implementation of a longitudinal low-frequency, high-intensity direct observation and coaching intervention, and elaborates on insights learned. Internal medicine interns at one residency training program were randomized to an ambulatory coaching intervention or usual precepting. Over one year, coached interns had three complete primary care visits directly observed by a faculty clinician-coach who provided feedback informed by a behavior checklist. Immediately after each of the coached patient encounters, interns completed a structured self-assessment and coaches led a 30-minute feedback session informed by intern self-reflection and checklist items. Interns with usual precepting had two mini-CEX observations over the course of the year without other formal direct observation in the ambulatory setting.  **Evaluation:** As part of the post-intervention assessment, senior faculty members blinded to intervention and control group assignments evaluated videotaped encounters. Coached interns completed an average of 21/23 behaviors from the checklist, while interns from the control group completed 18 (p < 0.05). The median overall grade for coached interns was B+, compared to B−/C+ for controls (p < 0.05).  **Reflection:** Coaching interns longitudinally using a behavior checklist is feasible and associated with improved consultation performance. Direct observation of complete clinical encounters followed by systematic coaching is educationally valuable, but time and resource intensive. |
| 43 | L. Pront, D. Gillham, and L.W. Schuwirth, | Competencies to enable learning‐focused clinical supervision: a thematic analysis of the literature | 2016 | Med Educ | **Context:** Clinical supervision is essential for development of health professional students and widely recognised as a significant factor influencing student learning. Although considered important, delivery is often founded on personal experience or a series of predetermined steps that offer standardised behavioural approaches. Such a view may limit the capacity to promote individualised student learning in complex clinical environments. The objective of this review was to develop a comprehensive understanding of what is considered 'good' clinical supervision, within health student education. The literature provides many perspectives, so collation and interpretation were needed to aid development and understanding for all clinicians required to perform clinical supervision within their daily practice.  **Method:** A comprehensive thematic literature review was carried out, which included a variety of health disciplines and geographical environments.  **Results:** Literature addressing 'good' clinical supervision consists primarily of descriptive qualitative research comprising mostly small studies that repeated descriptions of student and supervisor opinions of 'good' supervision. Synthesis and thematic analysis of the literature resulted in four 'competency' domains perceived to inform delivery of learning-focused or 'good' clinical supervision. Domains understood to promote student learning are co-dependent and include 'to partner', 'to nurture', 'to engage' and 'to facilitate meaning'.  **Conclusions:** Clinical supervision is a complex phenomenon and establishing a comprehensive understanding across health disciplines can influence the future health workforce. The learning-focused clinical supervision domains presented here provide an alternative perspective of clinical supervision of health students. This paper is the first step in establishing a more comprehensive understanding of learning-focused clinical supervision, which may lead to development of competencies for clinical supervision. |
| 44 | H. Birden, N. Glass, I. Wilson, M. Harrison, T. Usherwood, and D. Nass | Teaching professionalism in medical education: a Best Evidence Medical Education (BEME) systematic review. BEME Guide No. 25 | 2013 | Med Teach | **Introduction:** We undertook a systematic review to identify the best evidence for how professionalism in medicine should be taught.  **Methods:** Eligible studies included any articles published between 1999 and 2009 inclusive. We reviewed papers presenting viewpoints and opinions as well as empirical research. We performed a comparative and thematic synthesis on all papers meeting inclusion criteria in order to capture the best available evidence on how to teach professionalism.  **Results:** We identified 217 papers on how to teach professionalism. Of these, we determined 43 to be best evidence. Few studies provided comprehensive evaluation or assessment data demonstrating success. As yet, there has not emerged a unifying theoretical or practical model to integrate the teaching of professionalism into the medical curriculum.  **Discussion:** Evident themes in the literature are that role modelling and personal reflections, ideally guided by faculty, are the important elements in current teaching programmes, and are widely held to be the most effective techniques for developing professionalism. While it is generally held that professionalism should be part of the whole of a medical curriculum, the specifics of sequence, depth, detail, and the nature of how to integrate professionalism with other curriculum elements remain matters of evolving theory. |
| 45 | T. Singh and A. Singh | Abusive culture in medical education: Mentors must mend their ways | 2018 | J Anaesthesiol Clin Pharmacol | Bullying and harassment occur in all organizations, although rates seem to be higher in healthcare institutions, and such behavior may be more common in medical facilities. Despite promising efforts, student mistreatment remains an ongoing challenge in medical education, with published studies continuing to report high rates (80%–90%). Trainees, medical students, and female staff and colleagues are identified as the most likely targets.  Examples of mistreatment include sexual harassment; discrimination or harassment based on race, religion, ethnicity, gender, or sexual orientation; humiliation and psychological or physical punishment. One of the very first documentations with regard to student mistreatment in medicine was by Silver in 1982. Since the publication of this article, there has been increasing awareness of and research about medical student abuse, yet nothing much has changed in terms of the abusive culture of medical education; medical students are still being abused in the medical workplace. No review to date focuses on why this problem exists or how we might find solutions to reduce workplace harassment of residents.  For individuals, being exposed to bullying can have serious implications for mental and physical health including depression, helplessness, anxiety and despair, suicide ideation, psychosomatic and musculoskeletal complaints, and the risk of cardiovascular disease.Studies have found increased alcohol consumption, cigarette smoking, and drug usage and decreased satisfaction with residency and thoughts of desertion and even rethinking on their career choice in residents faced with increased stress and mistreatment. There is a clear link between bullying and the quality and effectiveness of training. A doctor in training who is subject to bullying behavior every day is much less likely to receive effective and fulfilling training. Critically for healthcare, doctors who were bullied were more likely to have committed one or more serious, or potentially serious, medical errors and can hamper the ability to provide effective and safe patient care. At an organizational level, the cost of bullying can also be substantial, taking into account absenteeism, turnover, and productivity. |
| 46 | A. Olaussen, P. Reddy, S. Irvine, and B. Williams, | Peer-assisted learning: time for nomenclature clarification | 2016 | Med Educ Online | **Background:** Peer-assisted learning (PAL) is used throughout all levels of healthcare education. Lack of formalised agreement on different PAL programmes may confuse the literature. Given the increasing interest in PAL as an education philosophy, the terms need clarification. The aim of this review is to 1) describe different PAL programmes, 2) clarify the terminology surrounding PAL, and 3) propose a simple pragmatic way of defining PAL programmes based on their design.  **Methods:** A review of current PAL programmes within the healthcare setting was conducted. Each programme was scrutinised based on two aspects: the relationship between student and teacher, and the student to teacher ratio. The studies were then shown to fit exclusively into the novel proposed classification.  **Results:** The 34 programmes found, demonstrate a wide variety in terms used. We established six terms, which exclusively applied to the programmes. The relationship between student and teacher was categorised as peer-to-peer or near-peer. The student to teacher ratio suited three groupings, named intuitively ‘Mentoring’ (1:1 or 1:2), ‘Tutoring’ (1:3–10), and ‘Didactic’ (1:>10). From this, six novel terms – all under the heading of PAL – are suggested: ‘Peer Mentoring’, ‘Peer Tutoring’, ‘Peer Didactic’, ‘Near-Peer Mentoring’, ‘Near-Peer Tutoring’, and ‘Near-Peer Didactic’.  **Conclusions:** We suggest herein a simple pragmatic terminology to overcome ambiguous terminology. Academically, clear terms will allow effective and efficient research, ensuring furthering of the educational philosophy |
| 47 | K. Gray, L. Annabell, and G. Kennedy | Medical students' use of Facebook to support learning: Insights from four case studies | 2010 | Med Teach | Recent research indicates that university students are interested and active in supporting their learning by using Facebook, a popular social networking website. This study aimed to add to our understanding of how or how effectively students may be using Facebook for this purpose. Researchers surveyed the extent and key features of Facebook use among 759 medical students at one university, and explored in depth the design and conduct of four Facebook study groups. 25.5% of students reported using Facebook for education related reasons and another 50.0% said they were open to doing so. The case studies showed conservative approaches in students' efforts to support their development of medical knowledge, skills and attributes in this way. Both technological affordances and group dynamics were factors contributing to groups' mixed successes. These cases indicate that using Facebook as part of learning and teaching is as much of a challenge for many students as it may be for most educators. |
| 48 | A. Klarare, J. Hansson, B. Fossum, C.J. Fürst, and C. Lundh Hagelin, | Team type, team maturity and team effectiveness in specialist palliative home care: an exploratory questionnaire study | 2019 | J Interprof Care | **Abstract:** To meet complex needs in persons and families within specialist palliative care, care team members are expected to work together in performing a comprehensive assessment of patient needs. Team type (how integrated team members work) and team maturity (group development) have been identified as components in team effectiveness and productivity. The aim of the study reported in this paper was to identify team types in specialist palliative care in Sweden, and to explore associations between team type, team maturity and team effectiveness in home care teams.  A national web-based survey of team types, based on Thylefors questionnaire, and a survey of healthcare professionals using the Group Development Questionnaire (GDQ-SE3) to assess team developmental phase, effectiveness and productivity were used in an exploratory cross-sectional design. The participants were: Specialist palliative care teams in Sweden registered in the Palliative Care Directory (n = 77), and members of 11 specialist palliative home care teams. Teams comprised physicians, registered nurses, social workers, physiotherapists and/or occupational therapists, full-or part-time. Our national web survey results showed that the 77 investigated teams had existed from 7 to 21 years, were foremost of medium size and functioned as inter- or transprofessional teams.  Results from the 61 HCPs, representing 11 teams, indicated that more mature teams tended to work in an integrated manner, rather than in parallel. The effectiveness ratio varied from 52% to 86% in teams. Recommendations arising from our findings include the need for clarification of team goals and professional roles together with prioritizing the development of desirable psychosocial traits and team processes in clinical settings. |
| 49 | A. Abu-Zaid | Protecting medical students against workplace research bullying: A graduate's experience and standpoint | 2020 | Educ Health (Abingdon) | Medical schools should offer rigorous educational programs to students and faculty on the fundamentals of research integrity, healthy research partnership, and successful mentor–mentee relationship. Additionally, medical schools should establish vigorous and easy-to-access institutional protocols for reporting such research-related ethical misconduct. Whistleblowers (student victims of authorship abuse) should be encouraged to disclose such practices and get protected, while perpetrators (faculty authorship bullies) should be penalized. It is time for research bullying, inclusive of all its overt and covert flavors, to be regarded as a research misconduct that warrants a serious disciplinary action. |
| 50 | L. S. Kwok, | The White Bull effect: abusive coauthorship and publication parasitism | 2005 | J Med Ethics | Junior researchers can be abused and bullied by unscrupulous senior collaborators. This article describes the profile of a type of serial abuser, the White Bull, who uses his academic seniority to distort authorship credit and who disguises his parasitism with carefully premeditated deception. Further research into the personality traits of such perpetrators is warranted. |
| 51 | D.M. Torre, B.J. Daley, J.L. Sebastian, and D.M. Elnicki, | Overview of current learning theories for medical educators | 2006 | Am J Med | **Purpose:** To explore how academic physicians perform social and professional identities and how their personal experiences inform professional identity formation.  **Method:** Semistructured interviews and observations were conducted with 25 academic physicians of diverse gender and racial/ethnic backgrounds at the University of Utah School of Medicine from 2015 to 2016. Interviews explored the domains of social identity, professional identity, and relationships with patients and colleagues. Patient interactions were observed. Interviews and observations were audio-recorded, transcribed, and analyzed using grounded theory.  **Results**: Three major themes emerged: Physicians' descriptions of identity differed based on social identities, as women and racially/ethnically minoritized participants linked their gender and racial/ethnic identities, respectively, to their professional roles more than men and white, non-Latino/a participants; physicians' descriptions of professional practice differed based on social identities, as participants who associated professional practices with personal experiences often drew from events connected to their minoritized identities; and physicians' interactions with patients corresponded to their self-described actions.  **Conclusions:** Professional identity formation is an ongoing process, and the negotiation of personal experiences is integral to this process. This negotiation may be more complex for physicians with minoritized identities. Implications for medical education include providing students, trainees, and practicing physicians with intentional opportunities for reflection and instruction on connecting personal experiences and professional practice. |
| 52 | B. Gormley | An application of attachment theory: Mentoring relationship dynamics and ethical concerns | 2008 | Mentor Tutoring: Partnersh Learn | In this theoretical paper, mentoring relationships are conceptualized as close relationships that occur along a spectrum from highly functional to highly dysfunctional, with most occurring in between. A complex set of factors describe the functioning level of mentoring relationships: (a) the attachment styles of the mentors and mentees; (b) interpersonal processes, including conflict management; (c) the quality of the relationship that results from combining the attachment styles of mentors and mentees; (d) mentoring outcomes; and (e) other contributors (e.g., gender or cultural differences, organizational climate). Healthy and unhealthy attachment styles among mentors and mentees and their contributions to mentoring processes and outcomes are presented. |
| 53 | D.C. Taylor and H. Hamdy | Adult learning theories: implications for learning and teaching in medical education: AMEE Guide No. 83 | 2013 | Med Teach | There are many theories that explain how adults learn and each has its own merits. This Guide explains and explores the more commonly used ones and how they can be used to enhance student and faculty learning. The Guide presents a model that combines many of the theories into a flow diagram which can be followed by anyone planning learning. The schema can be used at curriculum planning level, or at the level of individual learning. At each stage of the model, the Guide identifies the responsibilities of both learner and educator. The role of the institution is to ensure that the time and resources are available to allow effective learning to happen. The Guide is designed for those new to education, in the hope that it can unravel the difficulties in understanding and applying the common learning theories, whilst also creating opportunities for debate as to the best way they should be used. |
| 54 | C. Lewis and E. Olshansky, | Relational-cultural theory as a framework for mentoring in academia: Toward diversity and growth-fostering collaborative scholarly relationships | 2016 | Mentor Tutoring: Partnersh Learn | Abstract: Mentoring in academia that encourages collaboration and interpersonal relationships is important in helping newer faculty members attain success. Developing such programs is challenging within our prevailing academic context that rewards competition and individually delineated success. We propose that Relational Cultural Theory, a feminist approach to healthy psychological growth, developed by Jean Baker Miller and colleagues at the Stone Center at Wellesley College, is an appropriate framework to guide effective mentoring programs, with a particular focus on cross-cultural mentoring of protégés in academia who are women and/or of color. We suggest the traditionally individual-oriented definition of success in academia could be modified toward more emphasis on collaboration, recognizing, and celebrating the rich diversity within academia. This emphasis can strengthen organizations by increasing and embracing diversity, adding to the richness of ideas and approaches to societal problems. Mentoring is defined through an expanded view that recognizes healthy collaboration as an indicator of success in academia. |
| 55 | S. Heeneman and W. de Grave, | Tensions in mentoring medical students toward self-directed and reflective learning in a longitudinal portfolio-based mentoring system–an activity theory analysis | 2017 | Med Teach | **Background:** In medical education, students need to acquire skills to self-direct(ed) learning (SDL), to enable their development into self-directing and reflective professionals. This study addressed the mentor perspective on how processes in the mentor-student interaction influenced development of SDL.  **Methods**: n = 22 mentors of a graduate-entry medical school with a problem-based curriculum and longitudinal mentoring system were interviewed (n = 1 recording failed). Using activity theory (AT) as a theoretical framework, thematic analysis was applied to the interview data to identify important themes.  **Results:** Four themes emerged: centered around the role of the portfolio, guiding of students' SDL in the context of assessment procedures, mentor-role boundaries and longitudinal development of skills by both the mentor and mentee. Application of AT showed that in the interactions between themes tensions or supportive factors could emerge for activities in the mentoring process.  **Conclusion:** The mentors' perspective on coaching and development of reflection and SDL of medical students yielded important insights into factors that can hinder or support students' SDL, during a longitudinal mentor-student interaction. Coaching skills of the mentor, the interaction with a portfolio and the context of a mentor community are important factors in a longitudinal mentor-student interaction that can translate to students' SDL skills. |
| 56 | B.J. Irby, J.N. Boswell, L.J. Searby, F. Kochan, R. Garza, and N. Abdelrahman | The Wiley international handbook of mentoring | 2020 | John Wiley & Sons | In today’s networked world society, mentoring is a crucial area for study that requires a deep international understanding for effective implementation. Despite the immense benefits of mentoring, current literature on this subject is surprisingly sparse. The Wiley International Handbook of Mentoring fills the need for a comprehensive volume of in-depth information on the different types of mentoring programs, effective mentoring practices, and emerging practical and applicable theories. Based on sound research methodologies, this unique text presents original essays by experts from over ten different countries, demonstrating the ways mentoring can make a difference in the workplace and in the classroom; these experts have an understanding of mentoring worldwide having worked in mentoring in over forty countries.  . |
| 57 | P. E. S. Crampton and Y. Afzali | Professional identity formation, intersectionality and equity in medical education | 2021 | Med Educ | Considering the literature on Professional Identity Formation, the authors draw the powerful and alarming conclusion that it is almost devoid of exploration into how race and ethnicity have an influence |
| 58 | T.R. Wyatt, D. Balmer, N. Rockich-Winston, C.J. Chow, J. Richards, and Z. Zaidi | Whispers and shadows’: A critical review of the professional identity literature with respect to minority physicians | 2021 | Med Educ | **Objectives:** Professional identity formation (PIF) is a growing area of research in medical education. However, it is unclear whether the present research base is suitable for understanding PIF in physicians considered to be under-represented in medicine (URM). This meta-ethnography examined the qualitative PIF literature from 2012 to 2019 to assess its capacity to shine light on the experiences of minoritised physicians.  **Methods:** Data were gathered using a search of six well-known medical education journals for the term 'professional identity' in titles, keywords, abstracts and subheadings, delineated with the date range of 2012-2019. All non-relevant abstracts were removed and papers were then further reduced to those that focused only on learners' experiences. This left 67 articles in the final dataset, which were analysed using a collaborative approach among a team of researchers. The team members used their professional expertise as qualitative researchers and personal experiences as minoritised individuals to synthesise and interpret the PIF literature.  **Results:** Four conceptual categories were identified as impacting PIF: Individual versus Sociocultural Influences; the Formal versus the Hidden Curriculum; Institutional versus Societal Values; and Negotiation of Identity versus Dissonance in Identity. However, a major gap was identified; only one study explored experiences of PIF in URM physicians and there was an almost complete absence of critical stances used to study PIF. Combined, these findings suggest that PIF research is building on existing theories without questioning their validity with reference to minoritised physicians.  **Conclusions**: From a post-colonial perspective, the fact that race and ethnicity have been largely absent, invisible or considered irrelevant within PIF research is problematic. A new line of inquiry is needed, one that uses alternative frameworks, such as critical theory, to account for the ways in which power and domination influence PIF for URM physicians in order to foreground how larger sociohistorical issues influence and shape the identities of minoritised physicians |
| 59 | C.J. Chow, C.L. Byington, L.M. Olson, K.P.G. Ramirez, S. Zeng, and A.M. López | A Conceptual Model for Understanding Academic Physicians’ Performances of Identity: Findings From the University of Utah | 2018 | Acad Med | **Purpose:** To explore how academic physicians perform social and professional identities and how their personal experiences inform professional identity formation.  **Method:** Semi structured interviews and observations were conducted with 25 academic physicians of diverse gender and racial/ethnic backgrounds at the University of Utah School of Medicine from 2015 to 2016. Interviews explored the domains of social identity, professional identity, and relationships with patients and colleagues. Patient interactions were observed. Interviews and observations were audio-recorded, transcribed, and analyzed using grounded theory.  **Results:** Three major themes emerged: Physicians' descriptions of identity differed based on social identities, as women and racially/ethnically minoritized participants linked their gender and racial/ethnic identities, respectively, to their professional roles more than men and white, non-Latino/a participants; physicians' descriptions of professional practice differed based on social identities, as participants who associated professional practices with personal experiences often drew from events connected to their minoritized identities; and physicians' interactions with patients corresponded to their self-described actions.  **Conclusions:** Professional identity formation is an ongoing process, and the negotiation of personal experiences is integral to this process. This negotiation may be more complex for physicians with minoritized identities. Implications for medical education include providing students, trainees, and practicing physicians with intentional opportunities for reflection and instruction on connecting personal experiences and professional practice. |
| 60 | E. Ong and L. Krishna | Perspective from Singapore | 2014 | NUS Press | **Background:** Healthcare providers often struggle to balance the sometimes competing considerations of maximizing quality of life (QoL) and quantity of life with disease-modifying treatment (DMT). These decisions require in-depth dialog between all parties in order to understand the concerns and perspectives of the patient and caregiver in this period.  **Objectives:** The objectives of this study were to explore Singaporean patients’ and caregivers’ attitudes towards QoL and DMT, and to examine the reasons behind their beliefs.  Methods: Participants were given a video vignette of a family discussing how best to care for their mother who is recently diagnosed with cancer and were interviewed regarding their thoughts on QoL and DMT for a 70-year-old patient with stage IV metastatic cancer.  **Subjects/Setting:** A total of 21 patients and caregivers were recruited from a tertiary oncology centre.  **Results:** Both patients and caregivers show little support for pursuing QoL, despite the likely compromise to the patients’ QoL. These participants believed that not pursuing DMT was tantamount to giving up and accepting death. Whilst patients did accept that in some circumstances a QoL approach would be acceptable, caregivers remained adamant upon a DMT approach. The perspectives of caregivers reflected the influence of the Confucian-inspired practice of filial piety.  **Conclusion:** Local sociocultural beliefs and values continue to play a significant consideration in end-of-life decision-making. However, compliance with these beliefs have evolved, with greater consideration given to clinical and QoL factors. |
| 61 | E. Ong, L. Krishna, and P. Neo | The sociocultural and ethical issues behind the decision for artificial hydration in a young palliative patient with recurrent intestinal obstruction | 2015 | Ethics & Med | The decision to employ artificial hydration (AH) at the end of life is a complex process that must necessarily be made upon holistic consideration of a particular patient's situation. To highlight the complex interplay of ethical, clinical, practical, and psychosocial considerations behind such a decision, we discuss the decision-making process behind the determination to commence AH for a young 24-year-old Chinese woman with progressive metastatic ovarian adenocarcinoma who maintained a good functional status despite recurrent episodes of intestinal obstructions. |
| 62 | A. Surbone and L. Baider | Personal values and cultural diversity | 2013 | J Med Person | **Abstract:** Cross-cultural encounters in medicine are increasing in multi-ethnic societies and cultural pluralism enriches our lives. Yet, when values and ethical norms diverge in clinical encounters, bedside misunderstanding and conflicts can arise. Examples are the diverse different attitudes and practices of truth-telling, roles of families with regard to information and decision-making, approaches to end-of-life matters and significance of caregiving in different cultures. Understanding and negotiating these differences in the clinical setting is impossible without analyzing the meaning of culture and values. Culture shapes the experience and perception of illness and influences the coping mechanism adopted by each patient, through different values and belief systems. While a framework of common values underlies any therapeutic relationship, cultural differences may have major impact on the practice of medicine. In cultures centered on individual rights and freedom, for example, patient autonomy is the highest ethical value and patient information and active participation to the decision-making process are the norm. In contrast, in family and community-centered cultures, connectedness and reciprocal protection are highly valued, leading to lack of disclosure of medical truth and of patient involvement in decision-making. While in western cultures, cure is the highest goal of medicine, in cultures where suffering has redemptive meanings or endurance is supremely valued, traditional healing methods and goals differ. In this article, we attempt to clarify the complexity of cultural diversity with regard to values and norms that influence how people deal with illness, suffering, and dying by analyzing a clinical case and introducing the notion of cultural competence. |
| 63 | A. Au, | Online physicians, offline patients | 2018 | Int J Sociol Soc Policy | **Purpose:** The purpose of this paper is to demonstrate how the nature, gravity, and consequences of physician use of social media use surpass professional identity, by bringing to attention the nuanced, potential conflicts between patient-physician interests in current educational policies.  **Design:** Analyzing a case study of a physician publicly posting and commenting on many of his patients’ information, conversations, and medical conditions on social media.  **Findings:** Physician social media use carries many issues that concern ethics and the patient, rather than professional identity and the physician. In response, two sets of ethical standards are developed: one that deals with what constitutes impermissible behaviors online, and another that stipulates appropriate punishments for violations of these codes.  **Originality/value:** Most medical education policies and the literature have emphasized professional identity- formation with regards to physician use of social media, rather than ethics. Furthermore, no study exists that presents a clear, concrete, insider perspective at physicians’ improper use of social media. |
| 64 | D. Al-Abdulrazzaq, A. Al-Fadhli, and A. Arshad | Advanced medical students’ experiences and views on professionalism at Kuwait University | 2014 | BMC Med Educ | **Background:** Professionalism is a core competency in the medical profession worldwide. Numerous studies investigate how this competency is taught and learned. However, there are few reports on the students’ views and experiences with professionalism especially in the Arab world. Our aim was to explore the experiences and views of Kuwait final-year medical students on professionalism.  **Methods:** This was a questionnaire study of final-year medical students at Kuwait University (n = 95). Open- and close-ended questions were used to determine the students’ experiences and views on definition, teaching, learning, and assessment of professionalism.  **Results:** Eighty-five of the students completed the questionnaire (89.5%). A total of 252 attributes defining professionalism were listed by our respondents. The majority (98.0%) of these attributes were categorized under the CanMEDS theme describing professionalism as commitment to patients, profession, and society through ethical practice. The most helpful methods in learning about professionalism for the students were contact with positive role models, patients and families, and with their own families, relatives and peers. The students’ rating of the quality and quantity of teaching professionalism in the institution was quite variable. Despite this, 68.2% of the students felt very or somewhat comfortable explaining the meaning of medical professionalism to junior medical students. Almost half of the students felt that their education had always or sometimes helped them deal with professionally-challenging situations. Majority (77.6%) of the students thought that their academic assessments should include assessment of professionalism and should be used as a selection criterion in their future academic careers (62.3%). Most of the students discussed and sought advice regarding professionally-challenging situations from their fellow medical students and colleagues. Seventy-five (88.2%) students did not know which organizational body in the institution deals with matters pertaining to medical professionalism.  **Conclusion:** This study highlights the influence of the curriculum, the hidden curriculum, and culture on medical students’ perception of professionalism. Medical educators should take in account such influences when teaching and assessing professionalism. Future research should aim at creating a framework of competencies that addresses professionalism in a context suitable for the Arabian culture. |
| 65 | A. Byszewski, W. Hendelman, C. McGuinty, and G. Moineau | Wanted: role models - medical students’ perceptions of professionalism | 2012 | BMC Med Educ | **Background:** Transformation of medical students to become medical professionals is a core competency required for physicians in the 21st century. Role modeling was traditionally the key method of transmitting this skill. Medical schools are developing medical curricula which are explicit in ensuring students develop the professional competency and understand the values and attributes of this role. The purpose of this study was to determine student perception of professionalism at the University of Ottawa and gain insights for improvement in promotion of professionalism in undergraduate medical education.  **Methods:** Survey on student perception of professionalism in general, the curriculum and learning environment at the University of Ottawa, and the perception of student behaviors, was developed by faculty and students and sent electronically to all University of Ottawa medical students. The survey included both quantitative items including an adapted Pritzker list and qualitative responses to eight open ended questions on professionalism at the Faculty of Medicine, University of Ottawa. All analyses were performed using SAS version 9.1 (SAS Institute Inc. Cary, NC, USA). Chi-square and Fischer’s exact test (for cell count less than 5) were used to derive p-values for categorical variables by level of student learning.  **Results:** The response rate was 45.6% (255 of 559 students) for all four years of the curriculum. 63% of the responses were from students in years 1 and 2 (preclerkship). Students identified role modeling as the single most important aspect of professionalism. The strongest curricular recommendations included faculty-led case scenario sessions, enhancing interprofessional interactions and the creation of special awards to staff and students to “celebrate” professionalism. Current evaluation systems were considered least effective. The importance of role modeling and information on how to report lapses and breaches was highlighted in the answers to the open ended questions.  **Conclusions**: Students identify the need for strong positive role models in their learning environment, and for effective evaluation of the professionalism of students and teachers. Medical school leaders must facilitate development of these components within the MD education and faculty development programs as well as in clinical milieus where student learning occurs. |
| 66 | S. E. Smith, V. R. Tallentire, H. S. Cameron, and S. M. Wood | The effects of contributing to patient care on medical students' workplace learning | 2013 | Med Educ | **Context:** Previous research has suggested that as medical students become more senior, they should increasingly take on the roles they will enact as newly qualified doctors by con- tributing to patient care. However, student contribution to patient care carries inherent risks to patient safety. This study aimed to pro- vide students with a new opportunity to con- tribute to patient care and to use this as a platform from which to explore the influence of contributing to patient care on medical student learning.  **Method:** This study took place in the context of final-year medical student pre- scribing education at the University of Edin- burgh, Edinburgh, UK. Students on attachment at a district general hospital were afforded a unique opportunity to learn prescribing by completing in-patient drug charts in a process termed ‘pre-prescribing’. All students were invited to participate in focus groups conducted by the principal researcher. Focus group discussions were audio-recorded, transcribed verbatim and thematically analysed.  **Results:** Six focus groups, each lasting 20– 50 minutes, were conducted with four to seven participants (33 students in total). The emerging themes took the form of develop- mental outcomes and learning processes. Developmental outcomes included ability to perform the task, modification of attitudes towards the task, formation of a professional identity, and development of relationships within the team. The central feature of the experience which influenced all developmental outcomes, was making mistakes. The themes interact in complex ways and all con- tribute towards development as a professional.  **Conclusion:** This study has demonstrated that contributing to patient care enhances students’ development as professionals. Some of these developmental outcomes, such as improvements in knowledge and skills, may be achievable to some extent within the class- room. Other changes, such as developing relationships, forming a sense of professional identity and modifying attitudes, might arguably be achievable only within the context of contributing to patient care. |
| 67 | N. P. Kenny, K. V. Mann, and H. MacLeod | Role Modeling in Physicians’ Professional Formation: Reconsidering an Essential but Untapped Educational Strategy | 2003 | Acad Med | Role modeling remains one crucial area where standards are elusive and where repeated negative learning experiences may adversely impact the development of professionalism in medical students and residents. The literature is mainly descriptive, defining the attributes of good role models from both learners and practitioners’ perspectives. Because physicians are not “playing a role” as an actor might, but “embodying” different types of roles, the cognitive and behavioral processes associated with successfully internalizing roles (e.g., the good doctor/medical educator) are important.  In this article, the authors identify foundational questions regarding role models and professional character formation; describe major social and historical reasons for inattention to character formation in new physicians; draw insights about this important area from ethics and education theory (philosophical inquiry, apprenticeship, situated learning, observational learning, reflective practice); and suggest the practical consequences of this work for faculty recruitment, affirmation, and development. |
| 68 | N.D. Rosenblum, M. Kluijtmans, and O. Ten Cate | Professional Identity Formation and the Clinician–Scientist: A Paradigm for a Clinical Career Combining Two Distinct Disciplines | 2016 | Acad Med | This Perspective focuses on the challenges to the clinician– scientist’s professional identity and its development. First, the authors identify the particular challenges that arise from the different cultures of clinical care and science and the implications for clinician– scientist professional identity formation. Next, the authors synthesize insights about professional identity development within a dual-discipline career and apply their analysis to a discussion about the implications for clinician–scientist identity formation. Although not purposely developed to address identity formation, the authors highlight those elements within clinician–scientist training and career development programs that may implicitly support identity development. Finally, the authors highlight a need to identify empirically the elements that compose and determine clinician–scientist professional identity and the processes that shape its formation and sustainability. |
| 69 | E.M. Meyer, S. Zapatka, and R.S. Brienza | The Development of Professional Identity and the Formation of Teams in the Veterans Affairs Connecticut Healthcare System’s Center of Excellence in Primary Care Education Program (CoEPCE) | 2015 | Acad Med | **Purpose:** The United States Department of Veterans Affairs Connecticut Healthcare System (VACHS) is one of five Centers of Excellence in Primary Care Education (CoEPCE) pilot sites. The overall goal of the CoEPCE program, which is funded by the Office of Academic Affiliations, is to develop and implement innovative approaches for training future health care providers in postgraduate education programs to function effectively in teams to provide exceptional patient care. This longitudinal study employs theoretically grounded qualitative methods to understand the effect of a combined nursing and medical training model on professional identity and team development at the VACHS CoEPCE site.  **Method:** The authors used qualitative approaches to understand trainees’ experiences, expectations, and impressions of the program. From September 2011 to August 2012, they conducted 28 interviews of 18 trainees (internal medicine [IM] residents and nurse practitioners [NPs]) and subjected data to three stages of open, iterative coding.  **Results**: Major themes illuminate both the evolution of individual professional identity within both types of trainees and the dynamic process of group identity development. Results suggest that initially IM residents struggled to understand NPs’ roles and responsibilities, whereas NP trainees doubted their ability to work alongside physicians. At the end of one academic year, these uncertainties disappeared, and what was originally artificial had transformed into an  organic interprofessional team of health providers who shared a strong sense of understanding and trust.  **Conclusions:** This study provides early evidence of successful interprofessional collaboration among NPs and IM residents in a primary care training program. |
| 70 | C. Gilligan, T. Loda, F. Junne, S. Zipfel, B. Kelly, G. Horton, et al. | Medical identity; perspectives of students from two countries | 2020 | BMC Med Educ | **Background:** The development of professional identity is a fundamental element of medical education. There is evidence that in Germany, students’ perceptions of the ideal and real doctor differ, and that of themselves as physicians falls between these constructs. We sought to compare students’ perceptions of themselves, the ideal doctor, and the ‘real’ doctor and investigate differences from first to final year in the relationships between these constructs, as well as differences between Australian and German cohorts.  **Methods:** Students in the first and final years of their medical program at one Australian and one German university were invited to complete the Osgood and Hofstatter polarity profile, involving the description of their mental image of the ideal and real doctor, and the doctor they hope to become, with adjectives provided.  **Results:** One hundred sixty-seven students completed the survey in Australia (121 year 1, 46 year 5) and 188 in Germany (164 year 1, 24 year 6). The perception of the ideal doctor was consistent across all respondents, but that of the real doctor and self-image differed between country and year. Differences existed between country cohorts in perceptions of ‘confidence’, ‘strength’, ‘capability’ and ‘security’.  **Conclusions:** The pattern previously reported among German students was maintained, but a different pattern emerged among Australian students. Differences between countries could reflect cultural differences or variations in the overt and hidden curricula of medical schools. Some of the constructs within the profiles are amenable to educational interventions to improve students’ confidence and sense of capability. |
| 71 | X.M. Wang, M. Swinton, and J.J. You | Medical students’ experiences with goals of care discussions and their impact on professional identity formation | 2019 | Med Educ | **Context:** Goals of care (GoC) discussions occur amongst patients, family members and clinicians in order to establish plans of care and are invaluable aspects of end-of-life care. In previous research, medical learners have reported insufficient training and emotional distress about end-of-life decision making, but most studies have focused on postgraduate trainees and have been quantitative or have evaluated specific educational interventions. None have qualitatively explored medical students’ experiences with GoC discussions, their perceptions of associated hidden curricula, and the impacts of these on professional identity formation (PIF), the individualised developmental processes by which laypersons evolve to think, act and feel like, and ultimately become, medical professionals.  **Methods:** Using purposive sampling at one Canadian medical school, individual semi-structured interviews were conducted with 18 medical students to explore their experiences with GoC discussions during their core internal medicine clerkship. Interviews were audiorecorded, transcribed and anonymised. Concurrently with data collection, transcripts were analysed iteratively and inductively using interpretative phenomenological analysis, a qualitative research approach that allows the rich exploration of subjective experiences.  **Results**: Participants reported minimal support and supervision in conducting GoC discussions, which were experienced as ethically challenging, emotionally powerful encounters exemplifying tensions between formal and hidden curricula. Role modelling and institutional culture were key mechanisms through which hidden curricula were transmitted, subverting formal curricula in doing so and contributing to participants’ emotional distress. Participants’ coping responses were generally negative and included symptoms of burnout, the pursuit of standardisation, rationalisation, compartmentalisation and the adaptation of previously held, more idealised professional identities.  **Conclusions:** GoC discussions in this study were often led by inexperienced medical students and impacted negatively on their PIF. Through complex emotional processes, they struggled to reconcile earlier concepts of physician identities with newly developing ones and often reluctantly adopted suboptimal professional behaviours and attitudes. Improved education about GoC discussions is necessary for patient care and may represent concrete and specific opportunities to influence students’ PIF positively. |
| 72 | Y. Witman | What do we transfer in case discussions? The hidden curriculum in medicine… | 2014 | Perspect Med Educ | Medical students and junior doctors learn according to the formal curriculum, but they also learn unwritten rules, the specific logic of the medical world, through a socialization process called ‘the hidden curriculum’. The result of the process seems to be an exclusive professional identity, the medical habitus. This article underlines the importance of the hidden curriculum in medicine, especially in meetings where patients are discussed. These case discussions, common daily rituals in medical practice, demonstrate the dynamic interaction of learning and practice within the medical world. The article illustrates how medical core values are transferred informally and implicitly, and the role of power relations in this process. Not only are residents being assessed and trained in case discussions, but also staff are assessing each other and learning continuously. Therefore, these meetings might significantly contribute to self-regulation in medicine. The significance of the hidden curriculum should not be underestimated. Insights into the dynamics of case discussions may help medical specialists to make the most of this moment of learning and to avoid the pitfalls, for the benefit of both residents and experienced medical specialists. |
| 73 | F.W. Hafferty and R. Franks | The hidden curriculum, ethics teaching, and the structure of medical education | 1994 | Acad Med | The authors raise questions regarding the wide-spread calls emanating from lay and medical audiences alike to intensify the formal teaching of ethics within the medical school curriculum. In particular, they challenge a prevailing belief within the culture of medicine that while it may be possible to teach information about ethics (e.g., skills in recognizing the presence of common ethical problems, skills in ethical reasoning, or improved understanding of the language and concepts of ethics), course material or even an entire curriculum can in no way decisively influence a student's personality or ensure ethical conduct. To this end, several issues are explored, including whether medical ethics is best framed as a body of knowledge and skills or as part of one's professional identity. The authors argue that most of the critical determinants of physician identity operate not within the formal curriculum but in a more subtle, less officially recognized "hidden curriculum." The overall process of medical education is presented as a form of moral training of which formal instruction in ethics constitutes only one small piece. Finally, the authors maintain that any attempt to develop a comprehensive ethics curriculum must acknowledge the broader cultural milieu within which that curriculum must function. In conclusion, they offer recommendations on how an ethics curriculum might be more fruitfully structured to become a seamless part of the training process. |
| 74 | C. Whitehead, A. Kuper, R. Freeman, B. Grundland, and F. Webster | Compassionate care? A critical discourse analysis of accreditation standards | 2014 | Med Educ | **Context:** We rely upon formal accreditation and curricular standards to articulate the priorities of professional training. The language used in standards affords value to certain constructs and makes others less apparent. Leveraging standards can be a useful way for educators to incorporate certain elements into training. This research was designed to look for ways to embed the teaching and practice of compassionate care into Canadian family medicine residency training.  **Methods:** We conducted a Foucauldian critical discourse analysis of compassionate care in recent formal family medicine residency training documents. Critical discourse analysis is premised on the notion that language is connected to practices and to what is accorded value and power. We assembled an archive of texts and examined them to analyse how compassionate care is constructed, how notions of compassionate care relate to other key ideas in the texts, and the implications of these framings.  **Results:** There were very few words, metaphors or statements that related to concepts of compassionate care in our archive. Even potential proxies, notably the doctor-patient relationship and patient-centred care, were not primarily depicted in ways that linked them to ideas of compassion or caring. There was a reduction in language related to compassionate care in the 2013 standards compared with the standards published in 2006.  **Conclusions:** Our research revealed negative findings and a relative absence of the construct of compassionate care in our archival documents. This work demonstrates how a shift in curricular focus can have the unintended consequence of making values that are taken for granted less visible. Given that standards shape training, we must pay attention not only to what we include, but also to what we leave out of formal documents. We risk losing important professional values from training programmes if they are not explicitly highlighted in our standards. |
| 75 | L. Seoane, L.M. Tompkins, A. De Conciliis, and P.G. Boysen | Virtues Education in Medical School: The Foundation for Professional Formation | 2016 | Ochsner J | **Background:** Studies have shown that medical students have high rates of burnout accompanied by a loss of empathy as they progress through their training. This article describes a course for medical students at The University of Queensland-Ochsner Clinical School in New Orleans, LA, that focuses on the development of virtues and character strengths necessary in the practice of medicine. Staff of the Ochsner Clinical School and of the Institute of Medicine, Education, and Spirituality at Ochsner, a research and consulting group of Ochsner Health System, developed the course. It is a curricular innovation designed to explicitly teach virtues and their associated prosocial behaviors as a means of promoting professional formation among medical students. Virtues are core to the development of prosocial behaviors that are essential for appropriate professional formation.  **Methods**: Fourth-year medical students receive instruction in the virtues as part of the required Medicine in Society (MIS) course. The virtues instruction consists of five 3-hour sessions during orientation week of the MIS course and a wrap-up session at the end of the 8-week rotation. Six virtues-courage, wisdom, temperance, humanity, transcendence, and justice-are taught in a clinical context, using personal narratives, experiential exercises, contemplative practices, and reflective practices.  **Results:** As of July 2015, 30 medical students had completed and evaluated the virtues course. Ninety-seven percent of students felt the course was well structured. After completing the course, 100% of students felt they understood and could explain the character strengths that improve physician engagement and patient care, 100% of students reported understanding the importance of virtues in the practice of medicine, and 83% felt the course provided a guide to help them deal with the complexities of medical practice. Ninety-three percent of students stated they would use the character strengths for their own well-being, and 90% said they would change their approach to the practice of medicine as a result of this course. Overall, 92% of students rated the course as outstanding or good.  **Conclusion:** We developed a course to teach virtues and their associated prosocial behaviors that are important for the practice of medicine. After completing the course, students self-reported improved understanding of the virtues and their importance to the practice of medicine. We plan further studies to determine if participation in the course leads to less burnout and improved resilience. |
| 76 | E. Gaufberg, D. Bor, P. Dinardo, E. Krupat, E. Pine, B. Ogur, et al. | In Pursuit of Educational Integrity: Professional Identity Formation in the Harvard Medical School Cambridge Integrated Clerkship | 2017 | Perspect Biol Med | Graduates of Harvard Medical School’s Cambridge Integrated Clerkship (CIC) describe several core processes that may underlie professional identity formation (PIF): encouragement to integrate pre-professional and professional identities; support for learner autonomy in discovering meaningful roles and responsibilities; learning through caring relationships; and a curriculum and an institutional culture that make values explicit. The authors suggest that the benefits of educational integrity accrue when idealistic learners inhabit an educational model that aligns with their own core values, and when professional development occurs in the context of an institutional home that upholds these values. Medical educators should clarify and animate principles within curricula and learning environments explicitly in order to support the professional identity formation of their learners. |
| 77 | L. V. Monrouxe | Identity, identification and medical education: why should we care? | 2010 | Med Educ | **Context:** Medical education is as much about the development of a professional identity as it is about knowledge learning. Professional identities are contested and accepted through the synergistic internal-external process of identification that is constituted in and through language and artefacts within specific institutional sites. The ways in which medical students develop their professional identity and subsequently conceptualise their multiple identities has important implications for their own well-being, as well as for the relationships they form with fellow workers and patients.  **Objectives:** This paper aims to provide an overview of some current thinking about identity and identification with the aim of highlighting some of the core underlying processes that have relevance for medical educationists and researchers. These processes include aspects that occur within embodied individuals (e.g. the development of multiple identities and how these are conceptualised), processes specifically to do with interactional aspects of identity (e.g. how identities are constructed and co-constructed through talk) and institutional processes of identity (e.g. the influence of patterns of behaviour within specific hierarchical settings).  **Implications**: Developing a systematic understanding into the processes through which medical students develop their identities will facilitate the development of educational strategies, placing medical students' identification at the core of medical education.  **Conclusions:** Understanding the process through which we develop our identities has profound implications for medical education and entails that we adopt and develop new methods of collecting and analysing data. Embracing this challenge will provide better insights into how we might develop students' learning experiences, facilitating their development of a doctor identity that is more in line with desired policy requirements. |
| 78 | A.W. Chuang, F.S. Nuthalapaty, P.M. Casey, J.M. Kaczmarczyk, A.J. Cullimore, J.L. Dalrymple, et al. | To the point: reviews in medical education - taking control of the hidden curriculum | 2010 | Am J Obstet Gynecol | This article, the ninth in the "To the Point" series that is prepared by the Association of Professors of Gynecology and Obstetrics Undergraduate Medical Education Committee, discusses the role of the "hidden curriculum" in shaping the professional identity of doctors in training. The characteristics that distinguish the formal curriculum and hidden curriculum are defined. Specific examples of hidden curricula in clinical environments and the positive and negative impacts that may result are highlighted. Techniques to evaluate clinical training environments and to identify the hidden curriculum are provided and are followed by methods to promote its positive messages and lessen its negative ones. |
| 79 | D. Kay, A. Berry, and N.A. Coles | What Experiences in Medical School Trigger Professional Identity Development? | 2019 | Teach Learn Med | **Phenomenon:** This qualitative inquiry used conceptual change theory as a theoretical lens to illuminate experiences in medical school that trigger professional identity formation. According to conceptual change theory, changes in personal conceptualizations are initiated when cognitive disequilibrium is introduced. We sought to identify the experiences that trigger cognitive disequilibrium and to subsequently describe students' perceptions of self-in-profession prior to the experience; the nature of the experience; and, when applicable, the outcomes of the experience.  **Approach:** This article summarizes findings from portions of data collected in a larger qualitative study conducted at a new medical school in the United States that utilizes diverse pedagogies and experiences to develop student knowledge, clinical skills, attitudes, and dispositions. Primary data sources included focus groups and individual interviews with students across the 4 years of the curriculum (audio data). Secondary data included students' comments from course and end-of-year evaluations for the 2013-2017 classes (text data). Data treatment tools available in robust qualitative software, NVivo 10, were utilized to expedite coding of both audio and text data. Content analysis was adopted as the analysis method for both audio and text data.  **Findings:** We identified four experiences that triggered cognitive disequilibrium in relationship to students' perceptions of self-in-profession: (a) transition from undergraduate student to medical student, (b) clinical experiences in the preclinical years, (c) exposure to the business of medicine, and (d) exposure to physicians in clinical practice.  **Insights:** We believe these experiences represent vulnerable periods of professional identity formation during medical school. Educators interested in purposefully shaping curriculum to encourage adaptive professional identity development during medical school may find it useful to integrate educational interventions that assist students with navigating the disequilibrium that is introduced during these periods. |
| 80 | S. Jarvis-Selinger, D.D. Pratt, and G. Regehr | Competency Is Not Enough: Integrating Identity Formation Into the Medical Education Discourse | 2012 | Acad Med | Despite the widespread implementation of competency-based medical education, there are growing concerns that generally focus on the translation of physician roles into "measurable competencies." By breaking medical training into small, discrete, measurable tasks, it is argued, the medical education community may have emphasized too heavily questions of assessment, thereby missing the underlying meaning and interconnectedness of how physician roles shape future physicians. To address these concerns, the authors argue that an expanded approach be taken that includes a focus on professional identity development. The authors provide a conceptual analysis of the issues and language related to a broader focus on understanding the relationship between the development of competency and the formation of identities during medical training. Including identity alongside competency allows a reframing of approaches to medical education away from an exclusive focus on "doing the work of a physician" toward a broader focus that also includes "being a physician." The authors consider the salient literature on identity that can inform this expanded perspective about medical education and training. |
| 81 | H.D. Frost and G. Regehr | “I AM a Doctor”: Negotiating the Discourses of Standardization and Diversity in Professional Identity Construction | 2013 | Acad Med | Medical educators have expressed concern that students' professional identities do not always align with their expectations or with professional standards. The authors propose that, in constructing appropriate professional identities, medical students today are affected by the competing discourses of diversity and standardization.  Between March and May 2012, the authors conducted a critical review of seminal publications to highlight the discourses of diversity and standardization in the medical education literature. They surveyed the social sciences literature on identity construction and drew examples from medical education to demonstrate how a social constructionist approach could inform the discussion about how medical students' professional identities are affected by these discourses.  The discourse of diversity emphasizes individuality, difference, and a plurality of possibilities and advances the notion that heterogeneity is beneficial to medical education and to patients. In contrast, the discourse of standardization strives for homogeneity, sameness, and a limited range of possibilities and conveys that there is a single way to be a competent, professional physician. Thus, these discourses are in tension, a fact that medical educators largely have ignored. A social constructionist approach to identity suggests that medical students resolve this tension in different ways and construct different identities as a result. To influence medical students' professional identity construction, the authors advocate that educators seek change across the profession-faculty must acknowledge and take advantage of the tension between the discourses of standardization and diversity. |
| 82 | A. MacLeod | Caring, competence and professional identities in medical education | 2011 | Adv Health Sci Educ Theory Prac | This paper considers the multiple discourses that influence medical education with a focus on the discourses of competence and caring. Discourses of competence are largely constituted through, and related to, biomedical and clinical issues whereas discourses of caring generally focus on social concerns. These discourses are not necessarily equal partners in the enterprise of medical education. Discourses of competence tend to be privileged while those discourses of caring are often marginalised. Medical students learn to be physicians, and develop professional identities, in the context of these competing discourses. This paper documents a qualitative study designed to explore how professional identities are developed in the context of competing discourses. The study included a Foucauldian discourse analysis of medical education curriculum documents (67 problem-based learning cases in total), 26 h of observation of a small group learning experience (a problem-based learning tutorial), and in-depth, open-ended interviews with five medical students and nine medical educators at a Canadian medical school.  The paper describes how professional identities are developed in relation to discourses of competence, noting that students displayed what they considered to be desirable professional identities of confidence, capability and suitability. Also explored are the professional identities demonstrated in relation to discourses of caring, including those of benevolence and humbleness. Despite current conceptualisations, medical education is ripe with potential. The data indicate Foucauldian "spaces of freedom"-sites at which the complexity of the practice of medicine and the interwoven natures of the discourses of competence and caring might be taken into account as a means of challenging taken for granted cultural norms and broadening the medical gaze. |
| 83 | C. Rodríguez, S. López-Roig, T. Pawlikowska, F.X. Schweyer, E. Bélanger, M.A. Pastor-Mira | The influence of academic discourses on medical students' identification with the discipline of family medicine | 2015 | Acad Med | **Purpose:** To understand the influence of academic discourses about family medicine on medical students' professional identity construction during undergraduate training.  **Methods:** The authors used a multiple case study research design involving international medical schools, one each from Canada, France, Spain, and the United Kingdom (UK). The authors completed the fieldwork between 2007 and 2009 by conducting 18 focus groups (with 132 students) and 67 semi structured interviews with educators and by gathering pertinent institutional documents. They carried out discursive thematic analyses of the verbatim transcripts and then performed within- and cross-case analyses.  **Results:** The most striking finding was the diverging responses between those at the UK school and those at the other schools. In the UK case, family medicine was recognized as a prestigious academic discipline; students and faculty praised the knowledge and skills of family physicians, and students more often indicated their intent to pursue family medicine. In the other cases, family medicine was not well regarded by students or faculty. This was expressed overtly or through a paradoxical academic discourse that stressed the importance of family medicine to the health care system while decrying its lack of innovative technology and the large workload-to-income ratio. Students at these schools were less likely to consider family medicine.  **Conclusions**: These results stress the influence of academic discourses on medical students' ability to identify with the practice of family medicine. Educators must consider processes of professional identity formation during undergraduate medical training as they develop and reform medical education. |
| 84 | S. Warmington and G. McColl | Medical student stories of participation in patient care-related activities: the construction of relational identity | 2017 | Adv Health Sci Educ Theory Prac | **Abstract:** Professional identity formation is acknowledged as one of the fundamental tasks of contemporary medical education. Identity is a social phenomenon, constructed through participation in everyday activities and an integral part of every learning interaction. In this paper we report from an Australian ethnographic study into how medical students and patients use narrative to construct their identities. The dialogic narrative analysis employed focused on the production of meaning through the use of language devices in a given context, and the juxtaposition of multiple perspectives. Two stories told by students about their participation in patient care-related activities reveal how identities are constructed in this context through depictions of the relationships between medical students, patients and clinical teachers. These students use the rhetorical functions of stories to characterise doctors and patients in certain ways, and position themselves in relation to them. They defend common practices that circumvent valid consent processes, justified by the imperative to maximise students’ participation in patient care-related activities. In doing so, they identify patients as their adversaries, and doctors as allies. Both students are influenced by others’ expectations but one reveals the active nature of identity work, describing subtle acts of resistance. These stories illustrate how practices for securing students’ access to patients can influence students’ emerging identities, with implications for their future disclosure and consent practices. We argue that more collaborative ways of involving medical students in patient care-related activities will be facilitated if students and clinical teachers develop insight into the relational nature of identity work. |
| 85 | K. Foster and C. Roberts | The Heroic and the Villainous: a qualitative study characterising the role models that shaped senior doctors’ professional identity | 2016 | BMC Med Educ | **Background:** The successful development and sustaining of professional identity is critical to being a successful doctor. This study explores the enduring impact of significant early role models on the professional identity formation of senior doctors.  **Methods:** Personal Interview Narratives were derived from the stories told by twelve senior doctors as they recalled accounts of people and events from the past that shaped their notions of being a doctor. Narrative inquiry methodology was used to explore and analyse video recording and transcript data from interviews.  **Results:** Role models were frequently characterised as heroic, or villainous depending on whether they were perceived as good or bad influences respectively. The degree of sophistication in participants’ characterisations appeared to correspond with the stage of life of the participant at the time of the encounter. Heroes were characterised as attractive, altruistic, caring and clever, often in exaggerated terms. Conversely, villains were typically characterised as direct or covert bullies. Everyday events were surprisingly powerful, emotionally charged and persisted in participants’ memories much longer than expected. In particular, unresolved emotions dating from encounters where bullying behaviour had been witnessed or experienced were still apparent decades after the event.  **Conclusion:** The characterisation of role models is an important part of the professional identity and socialisation of senior doctors. The enduring impact of what role models say and do means that all doctors, need to consistently reflect on how their own behaviour impacts the development of appropriate professional behaviours in both students and training doctors. This is especially important where problematic behaviours occur as, if not dealt with, they have the potential for long-lasting undesirable effects. The importance of small acts of caring in building a nurturing and supportive learning atmosphere at all stages of medical education cannot be underestimated. |
| 86 | W. Hendelman and A. Byszewski | Formation of medical student professional identity: categorizing lapses of professionalism, and the learning environment | 2014 | BMC Med Educ | **Background:** Acquiring the values of medical professionalism has become a critical issue in medical education. The purpose of this study was to identify lapses in professionalism witnessed by medical students during their four year MD curriculum, and to categorize, from the students' perspective, who was responsible and the settings in which these occurred.  **Methods:** An electronic survey, developed by faculty and medical students, was sent to all students with two email reminders. It included quantitative responses and some open-ended opportunities for comments. All analyses were performed with SAS version 9.1.  **Results:** The response rate was 45.6% (255 of 559 students) for all four years of the medical school curriculum. Thirty six percent of students had witnessed or been part of an exemplary demonstration of professionalism; 64% responded that they had witnessed a lapse of professionalism. At the pre-clerkship level, the most frequent lapses involved students: arrogance (42.2%), impairment (24.2%), followed by cultural or religious insensitivity (20.5%). At the clerkship level of training, where students are exposed to real clinical situations, the lapses involved primarily faculty (including preceptor and clinician) or other staff; these included arrogance (55.3%), breach of confidentiality (28.3%), and cultural or religious insensitivity (26.6%); impairment involved mostly students (25.5%). These findings are analyzed from the perspective of role modeling by faculty and in the context of the learning environment.  **Conclusions:** Medical students witnessed a lapse of professionalism involving both fellow students as well as faculty and administrative staff, in several domains. Results from this study emphasize the importance of role modeling and the need for faculty development, to improve the learning environment. This study adds to the limited emerging literature on the forces that influence medical student professional identity formation. |
| 87 | R. Sternszus, J.D. Boudreau, R.L. Cruess, S.R. Cruess, M.E. Macdonald, and Y. Steinert | Clinical teachers’ perceptions of their role in professional identity formation | 2020 | Acad Med | **Purpose:** A fundamental goal of medical education is supporting learners in forming a professional identity. While it is known that learners perceive clinical teachers to be critically important in this process, the latter's perspective is unknown. This study sought to understand how clinical teachers perceive their influence on the professional identity formation of learners.  **Method:** In 2017, a research assistant conducted 16 semi structured interviews of clinical teachers from 8 specialties at McGill University. The research assistant audio recorded and subsequently transcribed interviews for analysis. Following principles of qualitative description, the research team developed a coding scheme using both inductive codes (from the words of the participants) and deductive codes (based on the literature and the theory of communities of practice). Through a cross-case analysis, the team then identified salient themes.  **Results:** Participants struggled to describe their influence on learners' professional identity without first being prompted to focus on their own identity and its formation. Once prompted, clinical teachers reported viewing their personal and professional identities as integrated and believed that caring for patients was integral to forming their professional identity. They identified explicit role modeling, engaging in difficult conversations, and providing graded autonomy as ways in which they could influence the identity development of learners. However, they had difficulty discerning the magnitude of their influence.  **Conclusions**: This study was the first to explore professional identity formation from the perspective of clinical teachers. The 2010 Carnegie Foundation report called for an increased focus on professional identity formation. Giving clinical teachers the space and guidance to reflect on this process, helping them make the implicit explicit, and supporting them in using their own experiences as learners to inform their teaching appear to be critical steps in achieving this goal. |
| 88 | S. Jarvis-Selinger, K.A. MacNeil, G. R.L. Costello, K. Lee, and C.L. Holmes | Understanding Professional Identity Formation in Early Clerkship: A Novel Framework | 2019 | Acad Med | **Purpose:** Medical educators should foster students' professional attitudes because individuals are more likely to act in accordance with medicine's professional values if these values have been internalized. Still, there is much to be learned about how students examine and negotiate their emerging identities. This study examined third-year medical students' experiences of professional identity formation (PIF) during clinical clerkship.  **Method:** The authors relied on an interpretivist perspective, informed by a grounded theory approach, to analyze data, which were collected from a pilot course designed to support medical students' efforts to "unhide" the hidden curriculum in relation to their development as medical students and emerging professionals.  **Results:** Twelve third-year medical students engaged in 10 collaborative discussions with 3 faculty members, a resident, and a fourth-year student (2015-2016). Discussions facilitated students' reflection on their professional journeys. Analysis of transcribed discussions resulted in a conceptual framework useful for exploring and understanding students' reflections on their PIF. Through analyzing students' experiences, the authors identified 4 components that constituted PIF stories: context, focus, catalyst, process.  **Conclusions:** The analysis resulted in the development of a conceptual framework and distinct identity formation themes. Discrete reflections focused on either students' current identity (being) or their sense of future self (becoming). The study identified catalysts that sparked participants' introspection about, or their processing of, identity. The moments that generate profound feelings of awareness in students are often moments that would not be recognizable (even post hoc) as remarkable by others. |
| 89 | H. Sadeghi Avval Shahr, S. Yazdani, and L. Afshar | Professional socialization: An analytical definition | 2019 | Ethics Hist Med | Professional socialization is defined as a process through which a person becomes a legitimate member of a professional society. This will have a great impact on an individual’s professional conduct and morality. The aim of this study was to clarify this concept and reduce the ambiguities around it.  This was a qualitative research through which the concept of professional socialization was analyzed using Walker and Avant’s eight-step approach. The review of literature for this concept was done using electronic database without any time limitation. The overall search produced about 780 articles, and after reviewing these articles, 21 were selected purposefully.  Based on concept analysis, we propose the following analytical definition: Professional socialization is a nonlinear, continuous, interactive, transformative, personal, psychosocial and self-reinforcing process that is formed through internalization of the specific culture of a professional community, and can be affected by individual, organizational and interactional factors. This definition is in accordance with the interactionism perspective. Existence of a particular profession and getting involved in a community of practice are the antecedents of this process, and formation of professional identity and professional development are its consequences. A case model, as well as borderline and related cases, has been introduced for this concept. The results of this study can be used to design useful educational interventions to conduct and facilitate the process. |
| 90 | H. Brody and D. Doukas | Professionalism: a framework to guide medical education | 2014 | Med Educ | **Context:** Despite considerable advances in the incorporation of professionalism into the formal curriculum, medical students and residents are too often presented with a mechanical, unreflective version of the topic that fails to convey deeper ethical and humanistic aspirations. Some misunderstandings of professionalism are exacerbated by commonly used assessment tools that focus only on superficially observable behaviour and not on moral values and attitudes.  **Methods:** Following a selective literature review, we engaged in philosophical ethical analysis to identify the key precepts associated with professionalism that could best guide the development of an appropriately reflective curriculum.  **Results:** The key precepts needed for a robust presentation of professionalism can be grouped under two headings: 'Professionalism as a trust-generating promise' (representing commitment to patients' interests, more than a mere business, a social contract, a public and collective promise, and hard work), and 'Professionalism as application of virtue to practice' (based on virtue, deeper attitudes rather than mere behaviour, and requiring of practical wisdom).  **Conclusions:** These key precepts help students to avoid many common, unreflective misunderstandings of professionalism, and guide faculty staff and students jointly to address the deeper issues required for successful professional identity formation. |
| 91 | R.L. Cruess, S.R. Cruess, J.D. Boudreau, L. Snell, and Y. Steinert | A Schematic Representation of the Professional Identity Formation and Socialization of Medical Students and Residents: A Guide for Medical Educators | 2015 | Acad Med | Recent calls to focus on identity formation in medicine propose that educators establish as a goal of medical education the support and guidance of students and residents as they develop their professional identity. Those entering medical school arrive with a personal identity formed since birth. As they proceed through the educational continuum, they successively develop the identity of a medical student, a resident, and a physician. Each individual's journey from layperson to skilled professional is unique and is affected by "who they are" at the beginning and "who they wish to become. “Identity formation is a dynamic process achieved through socialization; it results in individuals joining the medical community of practice. Multiple factors within and outside of the educational system affect the formation of an individual's professional identity. Each learner reacts to different factors in her or his own fashion, with the anticipated outcome being the emergence of a professional identity. However, the inherent logic in the related processes of professional identity formation and socialization may be obscured by their complexity and the large number of factors involved. Drawing on the identity formation and socialization literature, as well as experience gained in teaching professionalism, the authors developed schematic representations of these processes. They adapted them to the medical context to guide educators as they initiate educational interventions, which aim to explicitly support professional identity formation and the ultimate goal of medical education-to ensure that medical students and residents come to "think, act, and feel like a physician." |
| 92 | D.M. Irby and S. J. Hamstra | Parting the clouds: Three professionalism frameworks in medical education | 2016 | Acad Med | Current controversies in medical education associated with professionalism, including disagreements about curriculum, pedagogy, and assessment, are rooted in part in the differing frameworks that are used to address professionalism. Three dominant frameworks, which have evolved in the medical education community, are described. The oldest framework is virtue based and focuses on the inner habits of the heart, the development of moral character and reasoning, plus humanistic qualities of caring and compassion: The good physician is a person of character. The second framework is behavior based, which emphasizes milestones, competencies, and measurement of observable behaviors: The good physician is a person who consistently demonstrates competence in performing patient care tasks. The third framework is identity formation, with a focus on identity development and socialization into a community of practice: The good physician integrates into his or her identity a set of values and dispositions consonant with the physician community and aspires to a professional identity reflected in the very best physicians.  Although each professionalism framework is useful and valid, the field of medical education is currently engaged in several different discourses resulting in misunderstanding and differing recommendations for strategies to facilitate professionalism. In this article, the assumptions and contributions of each framework are described to provide greater insight into the nature of professionalism. By examining each discourse in detail, underlying commonalities and differences can be highlighted to assist educators in more effectively creating professionalism curricula, pedagogy, and assessment. |
| 93 | N. Huda, L. Faden, C.A. Wilson, R A. Plouffe, E. Li, M.K. Saini, et al. | The ebb and flow of identity formation and competence development in sub-specialty residents: Study of a continuity training setting | 2020 | Nil [preprint] | **Background:** Professional identity and competence development are evolving processes, shaped by clinical experiences and socialization in the workplace. The purpose of this study was to investigate the simultaneous development of professional identify formation and competence in a sub-specialty training program.  **Methods:** The study was conducted in a General Internal Medicine sub-specialty (PGY-4 and PGY-5) continuity training setting, at an academic health sciences center, in Canada. Participants included: current residents, recent graduates, attending physicians and administrative assistants. Data was collected from 2017–2018. A constructivist grounded theory approach was used to analyze anonymized focus group and individual interviews.  **Results:** The study identified the following: 1) learning activities that support professional identity formation in advanced residents; 2) the relationship between professional identity formation and competencies; 3) the role of administrative assistants and continuity training supervisors in supporting professional identity formation; and 4) a set of invisible learning experiences that occurred as a result of assumptions made by residents about expectations of training and expectations of patient care. Although, there was limited data available on the latter, findings suggested that invisible learning experiences may adversely impact residents’ functioning as independent physicians.  **Conclusions:** Residents’ professional identities continue to evolve with increasing competency requirements during training. Training programs, for sub-specialty residents, must balance granting of independence with supporting ongoing professional identity formation. They must also be explicit about what constitutes healthy patient care expectations and how practicing physicians manage these expectations. |
| 94 | R. Remmen, J. Denekens, A. Scherpbier, I. Hermann, C. van der Vleuten, P.V. Royen, et al. | An evaluation study of the didactic quality of clerkships | 2000 | Med Educ | **Context:** Previous qualitative research at the University of Antwerp revealed dissatisfaction amongst medical students about clinical clerkships.  **Objective**: To use quantitative methods to further explore student perceptions of their clinical teaching.  **Method, sample and setting:** Monthly questionnaire administered to final-year medical students at the University of Antwerp, Belgium.  **Results:** The response rate was 83.9%. Many of the qualitative findings were reproduced. Educational resources were not optimally used. The junior doctor was the most important clinical teacher. Many activities were passive experiences. Coaching, feedback and supervision associated most with general satisfaction of the clerkship, however, these dimensions were often considered suboptimal.  **Conclusions:** Clinical clerkships do not automatically provide an ideal learning environment for medical students |
| 95 | D.F. Balmer, J.R. Serwint, S.B. Ruzek, and A.P. Giardino | Understanding paediatric resident-continuity preceptor relationships through the lens of apprenticeship learning | 2008 | Med Educ | **Context:** Apprenticeship learning is common in medical education, but is often situated in theoretical frameworks which highlight its cognitive but not its social dimension.  **Methods:** We conducted an ethnographic case study of paediatric residents' learning relationships with their preceptors in a community-based paediatric continuity site. It included 5 months (100 hours) of direct observation, and semi-structured interviews with 10 residents (before and after observation) and 10 primary care paediatricians who served as their continuity preceptors (after observation). Interview transcripts and notes from observations were inductively coded and analysed for major themes.  **Results:** Our observations and reports of resident learning trajectories fit well with the concept of legitimate peripheral participation. Residents learned the everyday practice of primary care as they worked alongside experienced paediatricians in the continuity clinic. Although the direction of learning was towards central participation in patient care, residents learned during transient shifts to the periphery of practice. As a function of residents' increased participation, preceptors moved into more supportive roles. Residents were not only learners; at times they were teachers who facilitated preceptors' learning.  **Conclusions:** Legitimate peripheral participation is a concept that helps to explain apprenticeship as a dynamic social relationship which shapes, and is shaped by, learning that takes place in clinical practice. Other concepts shed light on the bidirectional nature of apprenticeship learning. |
| 96 | A. Bleakley | Pre-registration house officers and ward-based learning: a 'new apprenticeship' model | 2002 | Med Educ | **Introduction:** The pre-registration house officer (PRHO) year can be seen as a formal apprenticeship into the profession of medicine, and as central to the identity construction of the doctor. The year characteristically involves rotation between specialties, including attachment to ward-based 'firms', where consultants teach PRHOs.  **Discussion:** Teaching and learning in ward-based environments is under-researched, and the current literature displays a bias towards a psychological model of pedagogy that focuses upon transmission of knowledge and skills from one individual to another. Such a model offers a necessary, but not sufficient, explanation of how work-based learning occurs. Understanding the PRHO apprenticeship year should include reference to cultural dimensions to learning, especially socialisation into the profession. This constitutes an 'extended' (or 'hidden') curriculum model that may be theorised through contemporary ideas of activity learning within a 'new apprenticeship' framework.  **Conclusion:** The dominant psychological model can lead to an expectation for a uniform method of teaching and learning in ward round contexts that (a) ignores important differences in educational climate between established communities of practice, and (b) orients both teachers and learners to one-to-one transmission and reception, rather than sensitising to how knowledge may be held across members of a working group. The latter shifts emphasis away from reception to issues of active access. PRHOs, as novices, are not relegated to passive learning roles, but may actively co-construct knowledge with experts, offering potential transformation of the practices of ward groups. |
| 97 | S.M. Wearne, L. Butler, and J.A. Jones | Educating registrars in your practice | 2016 | Aust Fam Physician | **Background:** Postgraduate general practice training is an apprenticeship of learning to be a general practitioner by working as a general practitioner under supervision. During this apprenticeship, registrars learn the art, craft and ethics of their vocation, and how to apply clinical knowledge and skills in different contexts.  **Objective:** This article summarises how registrars learn, and the role of general practice supervisors and training practices in supporting their education.  **Discussion:** General practice supervisors form educational alliances with registrars that provide the foundation for deep and broad learning. Overseeing patient safety requires supervisors to respond to registrars' queries and monitor them proactively. Registrars learn best in practices that include them in all their work and share their expertise. Specific issues raised by teams of part-time super-visors, and by rural and remote practice, are discussed. Finally, teaching practices are recommended to seek and implement feedback from registrars. |
| 98 | H.G.A.R. Jochemsen-Van Der Leeuw, N. Buwalda, M. Wieringa-De Waard, and N. Van Dijk | Learning from a role model: A cascade or whirlpool effect? | 2015 | Med Teach | **Background:** Continuing Professional Development (CPD) and Faculty Development (FD) courses have been designed in the expectation that a cascade effect will occur, consisting of a conveyance of information from the courses to clinical trainers to daily practice and/or to trainees by means of role modeling.  **Purpose:** The aims of this study were to gain insight into factors that encourage clinical trainers to incorporate what they have learned in CPD/FD into their role model function and the factors that influence conveyance from master to apprentice.  **Method:** We conducted a qualitative study using semi-structured interviews with GP trainers and their trainees.  **Results:** Twenty-four GP trainers who completed a CPD/FD course and sixteen of their trainees participated in the study. Analysis of their statements enabled the identification of factors that affect the amplification of the competences of clinical trainers and their awareness of being a role model, the applicability in training practice and conveyance to the trainee.  **Conclusions:** As a result of interactions between the trainer, trainee and patient, it seems more accurate to represent the conveyance of competences from master to apprentice using the image of a whirlpool rather than a cascade, with the influential factors and interactions functioning as filters, causing a decline in the effectiveness of CPD/FD. Using the filters as a basis for turning-points for improvements around the whirlpool could increase the effectiveness of CPD/FD. |
| 99 | E. Lightman, S. Kingdon, and M. Nelson | A prolonged assistantship for final-year students | 2015 | Clin Teach | **Background:** The transition from medical student to junior doctor is challenging, therefore adequate preparation during medical school is crucial for a smooth transition. Tomorrow's Doctors expects students to undertake a student assistantship, separate from the local shadowing period prior to commencing employment. Sheffield Medical School initiated a 6-week assistantship within the Yorkshire and Humber deanery. This mixed-methodology study explores this experience from the perspective of final-year medical students.  **Methods:** Final-year medical students responded to a questionnaire about anxieties surrounding their forthcoming foundation year 1 (FY1). Students were purposefully sampled and semi-structured interviews (SSIs) were conducted, with 20 participants exploring their experience of the assistantship. Interviews were transcribed verbatim and thematic analysis was carried out.  **Results:** The questionnaire results highlighted that most students felt anxious about becoming a FY1 doctor, and subsequent interviews demonstrate that the assistantship mostly met with students' expectations. Major themes regarding their anxieties included the value of building professional relationships, familiarisation with the work environment, level of supervision and the value of targeted teaching.  **Discussion:** Student assistantships clearly improve preparedness and confidence in the transition to FY1 by enabling supervised, stepwise responsibility. In the future a nationally cohesive programme should be created for shadowing and assistantships matched with students' prospective jobs. |
| 100 | P. Nirodi, H. El-Sayeh, and H. Henfrey | Applying the apprenticeship model to psychiatry: An evaluation | 2018 | Neurol Psychiatry | Working within the ‘firm’, long the bedrock of medical student clinical education, has been in recent demise. This article assesses the success of an apprenticeship scheme piloted within a mental health setting for fourth year medical students. The results show that it was possible and indeed successful in terms of fulfilling the basic tenets of the apprenticeship model. It was also found to be a novel method of increasing the likelihood of students considering psychiatry as a future career. |
| 101 | N. Ratanawongsa, A. Teherani, and K. E. Hauer | Third-year medical students' experiences with dying patients during the internal medicine clerkship: a qualitative study of the informal curriculum | 2005 | Acad Med | **Purpose:** To explore third-year medical students' experiences with death and dying patients during the first internal medicine clerkship.  **Method:** In August 2002, through purposeful sampling, the authors targeted for open-ended interviews 32 third-year medical students at the University of California, San Francisco in the first core internal medicine clerkship. Interviews averaged 45 minutes in length and were audiotaped, transcribed, and analyzed using a grounded theory approach.  **Results:** Twenty-eight (87.5%) students participated in interviews. All students encountered death or dying patients, and most cared directly for at least one dying patient. Students' relationships with patients were characterized by attachment, empathy, and advocacy. Students valued preparation by preclinical end-of-life (EOL) courses, but assigned greater value to patient care experiences guided by teams that acknowledged deaths, role-modeled EOL care, and respected students' participation in patient care. Clerkship experiences in EOL care affected students' developing professional identities by affording opportunities to manage strong emotions, understand the challenges of transitioning to residency, and gain a sense of self-efficacy as future physicians providing EOL care.  **Conclusions:** Third-year medical students' experiences with dying patients affect their skills and attitudes in EOL care, as well as the emergence of their professional identities. The behaviors and attitudes modeled by residents and attendings during the clerkships can strongly influence students' perceptions of and self-efficacy in EOL care. Further research and interventions into how residents and attendings model responses to death in the clinical clerkship may suggest strategies not only for EOL training, but also for mentoring professional development. |
| 102 | S. Dréano-Hartz, W. Rhondali, M. Ledoux, M. Ruer, J. Berthiller, A.-M. Schott, et al. | Burnout among physicians in palliative care: Impact of clinical settings | 2016 | Palliat Support Care | **Objective:** Burnout syndrome is a work-related professional distress. Palliative care physicians often have to deal with complex end-of-life situations and are at risk of presenting with burnout syndrome, which has been little studied in this population. Our study aims to identify the impact of clinical settings (in a palliative care unit (PCU) or on a palliative care mobile team (PCMT)) on palliative care physicians.  **Method:** We undertook a cross-sectional study using a questionnaire that included the Maslach Burnout Inventory (MBI), and we gathered sociodemographic and professional data. The questionnaire was sent to all 590 physicians working in palliative care in France between July of 2012 and February of 2013.  **Results:** The response rate was 61, 8% after three reminders. Some 27 (9%) participants showed high emotional exhaustion, 12 (4%) suffered from a high degree of depersonalization, and 71 (18%) had feelings of low personal accomplishment. Physicians working on a PCMT tended (p = 0.051) to be more likely to suffer from emotional exhaustion than their colleagues. Physicians working on a PCMT worked on smaller teams (fewer physicians, p < 0.001; fewer nonphysicians, p < 0.001). They spent less time doing research (p = 0.019), had fewer resources (p = 0.004), and their expertise seemed to be underrecognized by their colleagues (p = 0.023).  **Significance of results:** The prevalence of burnout in palliative care physicians was low and in fact lower than that reported in other populations (e.g., oncologists). Working on a palliative care mobile team can be a more risky situation, associated with a lack of medical and paramedical staff. |
| 103 | M.Y.H. Koh, A.Y. M. Hum, H.S. Khoo, A.H.Y. Ho, P.H. Chong, W.Y. Ong, et al. | Burnout and Resilience After a Decade in Palliative Care: What Survivors Have to Teach Us. A Qualitative Study of Palliative Care Clinicians With More Than 10 Years of Experience | 2020 | J Pain Symptom Manage | **Context:** Burnout is common among palliative care clinicians (PCCs). Resilience helps to reduce burnout, compassion fatigue, and is associated with longevity in palliative care.  **Objectives:** We aimed to study PCCs who have remained in the field for longer than 10 years to deepen our understanding on their views on burnout and resilience.  **Methods:** We conducted a qualitative study using semi structured interviews and purposive sampling on 18 PCCs - five doctors, 10 nurses, and three social workers who worked in various palliative care settings (hospital palliative care team, home hospice, and inpatient hospice). The mean age of the interviewees was 52 years, and the mean number of years practicing palliative care was 15.7 years (range 10-25). The interviews were recorded verbatim, transcribed, and analyzed using a grounded theory approach.  **Results:** Four major themes emerged from our analysis - struggling, changing mindset, adapting, and resilience. Intervening conditions, such as self-awareness, reflection, and evolution, were also important factors. The core phenomenon of our study was that of transformational growth - a process that PCCs have to go through before they achieve resilience. We also further classified resilience into both personal and collective resilience.  **Conclusion:** Our findings highlight the evolving process of transformational growth that PCCs must repeatedly undergo as they strive toward sustained resilience and longevity. It also stresses the importance of taking individual and collective responsibility toward building a culture of personal and team resilience. |
| 104 | R.H. Lehto, C. Heeter, J. Forman, T. Shanafelt, A. Kamal, P. Miller, et al. | Hospice Employees’ Perceptions of Their Work Environment: A Focus Group Perspective | 2020 | Int J Environ Res Public Health | Burnout in healthcare professionals can lead to adverse effects on physical and mental health, lower quality of care, and workforce shortages as employees leave the profession. Hospice professionals are thought to be at particularly high risk for burnout. The purpose of the study was to evaluate workplace perceptions of interdisciplinary hospice care workers who provide care to patients at end of life. Six focus groups and one semi-structured interview were conducted with mixed group of social workers, managers, nurses, hospice aides, chaplains, support staff, and a physician (n = 19). Findings from the groups depicted both rewards and challenges of hospice caregiving. Benefits included intrinsic satisfaction from the work, receiving positive patient and family feedback, and teamwork. Challenges reflected issues with workload, technology issues, administrative demands, travel-related problems, communication and interruptions, difficulties with taking time off from work and maintaining work-life integration, and coping with witnessing grief/loss. Hospice workers glean satisfaction from making meaningful differences in the lives of patients with terminal illness and their family members. It is an expected part of the job that certain patients and situations are particularly distressing; team support and targeted grief support is available for those times. Participants indicated that workload and administrative demands rather than dealing with death and dying were the biggest contributors to burnout. Participants reported episodic symptoms of burnout followed by deliberate steps to alleviate these symptoms. Notably, for all except one of the participants, burnout was cyclical. Symptoms would begin, they would take steps to deal with it (e.g., taking a mental health day), and they recovered. At an organizational level, a multipronged approach that includes both personal and occupational strategies is needed to support professional caregivers and help mitigate the stressors associated with hospice work. |
| 105 | M.Y.H. Koh, P.H. Chong, P.S.H. Neo, Y.J. Ong, W.C. Yong, W.Y. Ong, et al. | Burnout, psychological morbidity and use of coping mechanisms among palliative care practitioners: A multi-centre cross-sectional study | 2015 | Palliat Med | **Background:** The prevalence of burnout, psychological morbidity and the use of coping mechanisms among palliative care practitioners in Singapore have not been studied.  **Aim:** We aimed to study the prevalence of burnout and psychological morbidity among palliative care practitioners in Singapore and its associations with demographic and workplace factors as well as the use of coping mechanisms.  **Design:** This was a multi-centre, cross-sectional study of all the palliative care providers within the public healthcare sector in Singapore.  **Setting/participants:** The study was conducted in hospital palliative care services, home hospice and inpatient hospices in Singapore. The participants were doctors, nurses and social workers.  **Results:** The prevalence of burnout among respondents in our study was 91 of 273 (33.3%) and psychological morbidity was 77 (28.2%). Working >60 h per week was significantly associated with burnout (odds ratio: 9.02, 95% confidence interval: 2.3-35.8, p = 0.002) and psychological morbidity (odds ratio: 7.21, 95% confidence interval: 1.8-28.8, p = 0.005). Home hospice care practitioners (41.5%) were more at risk of developing psychological morbidity compared to hospital-based palliative care (17.5%) or hospice inpatient care (26.0%) (p = 0.007). Coping mechanisms like physical well-being, clinical variety, setting boundaries, transcendental (meditation and quiet reflection), passion for one's work, realistic expectations, remembering patients and organisational activities were associated with less burnout.  **Conclusion:** Our results reveal that burnout and psychological morbidity are significant in the palliative care community and demonstrate a need to look at managing long working hours and promoting the use of coping mechanisms to reduce burnout and psychological morbidity. |
| 106 | A.L. Back, K E. Steinhauser, A.H. Kamal, and V.A. Jackson | Building Resilience for Palliative Care Clinicians: An Approach to Burnout Prevention Based on Individual Skills and Workplace Factors | 2016 | J Pain Symptom Manage | For palliative care (PC) clinicians, the work of caring for patients with serious illness can put their own well-being at risk. What they often do not learn in training, because of the relative paucity of evidence-based programs, are practical ways to mitigate this risk. Because a new study indicates that burnout in PC clinicians is increasing, we sought to design an acceptable, scalable, and testable intervention tailored to the needs of PC clinicians. In this article, we describe our paradigm for approaching clinician resilience, our conceptual model, and curriculum for a workplace resilience intervention for hospital-based PC teams. Our paradigm for approaching resilience is based on upstream, early intervention. Our conceptual model posits that clinician well-being is influenced by personal resources and work demands. Our curriculum for increasing clinician resilience is based on training in eight resilience skills that are useful for common challenges faced by clinicians. To address workplace issues, our intervention also includes material for the team leader and a clinician perception survey of work demands and workplace engagement factors. The intervention will focus on individual skill building and will be evaluated with measures of resilience, coping, and affect. For PC clinicians, resilience skills are likely as important as communication skills and symptom management as foundations of expertise. Future work to strengthen clinician resilience will likely need to address system issues more directly. |
| 107 | D. Kavalieratos, D. E. Siconolfi, K.E. Steinhauser, J. Bull, R.M. Arnold, K.M. Swetz, et al. | It Is Like Heart Failure. It Is Chronic...and It Will Kill You": A Qualitative Analysis of Burnout Among Hospice and Palliative Care Clinicians | 2017 | J Pain Symptom Manage | **Context:** Although prior surveys have identified rates of self-reported burnout among palliative care clinicians as high as 62%, limited data exist to elucidate the causes, ameliorators, and effects of this phenomenon.  Objectives: We explored burnout among palliative care clinicians, specifically their experiences with burnout, their perceived sources of burnout, and potential individual, interpersonal, organizational, and policy-level solutions to address burnout.  **Methods:** During the 2014 American Academy of Hospice and Palliative Medicine/Hospice and Palliative Nurses Association Annual Assembly, we conducted three focus groups to examine personal narratives of burnout, how burnout differs within hospice and palliative care, and strategies to mitigate burnout. Two investigators independently analyzed data using template analysis, an inductive/deductive qualitative analytic technique.  **Results:** We interviewed 20 palliative care clinicians (14 physicians, four advanced practice providers, and two social workers). Common sources of burnout included increasing workload, tensions between nonspecialists and palliative care specialists, and regulatory issues. We heard grave concerns about the stability of the palliative care workforce and concerns about providing high-quality palliative care in light of a distressed and overburdened discipline. Participants proposed antiburnout solutions, including promoting the provision of generalist palliative care, frequent rotations on-and-off service, and organizational support for self-care. We observed variability in sources of burnout between clinician type and by practice setting, such as role monotony among full-time clinicians.  **Conclusion:** Our results reinforce and expand on the severity and potential ramifications of burnout on the palliative care workforce. Future research is needed to confirm our findings and investigate interventions to address or prevent burnout. |
| 108 | G. Ercolani, S. Varani, B. Peghetti, L. Franchini, M.B. Malerba, R. Messana, et al. | Burnout in Home Palliative Care: What Is the Role of Coping Strategies? | 2020 | J Palliat Care | **Objective:** The study examines psychophysical distress of health-care professionals providing home-based palliative care. The aim is to investigate potential correlations between dimensions of burnout and different coping strategies.  **Methods:** The present study is an observational cross-sectional investigation. The study involved all the home palliative care teams of an Italian nonprofit organization. Of a total of 275 practitioners working for the organization, 207 (75%) decided to participate in the study and complete questionnaires. Questionnaires employed were Maslach Burnout Inventory, General Health Questionnaire 12, Psychophysiological Questionnaire of CBA 2.0, and Coping Orientation to Problems Experienced. Professionals were physicians (50%), nurses (36%), and psychologists (14%). There were no exclusion criteria. Data were processed by SPSS 23 and analyses employed were Spearman ρ, Mann-Whitney U test, and 1-way analysis of variance on ranks.  **Results:** Among participants, a low number of professionals were emotionally exhausted (11%) or not fulfilled at work (20%), whereas most of them complained of depersonalization symptoms (67%). Emotional exhaustion and depersonalization were found to be associated with avoidance coping strategies, whereas problem-solving and positive attitude were negatively associated with emotional exhaustion and positively with personal accomplishment. Moreover, using avoidance strategies was related to a worse psychological and physical condition.  **Conclusions:** Findings suggest the need to provide professionals training programs about coping and communication skills tailored to fit the professionals' needs according to their work experience in palliative care and aimed at improving the approach to patients and relatives. |
| 109 | B.Y.Q. Tan, A. Kanneganti, L.J.H. Lim, M. Tan, Y.X. Chua, L. Tan, et al. | Burnout and Associated Factors Among Health Care Workers in Singapore During the COVID-19 Pandemic | 2020 | J Am Med Dir Assoc | **Objectives:** The strain on health care systems due to the COVID-19 pandemic has led to increased psychological distress among health care workers (HCWs). As this global crisis continues with little signs of abatement, we examine burnout and associated factors among HCWs.  **Design:** Cross-sectional survey study.  **Setting and participants**: Doctors, nurses, allied health professionals, administrative, and support staff in 4 public hospitals and 1 primary care service in Singapore 3 months after COVID-19 was declared a global pandemic.  **Methods:** Study questionnaire captured demographic and workplace environment information and comprised 3 validated instruments, namely the Oldenburg Burnout Inventory (OLBI), Safety Attitudes Questionnaire (SAQ), and Hospital Anxiety and Depression Scale (HADS). Multivariate mixed model regression analyses were used to evaluate independent associations of mean OLBI-Disengagement and -Exhaustion scores. Further subgroup analysis was performed among redeployed HCWs.  **Results:** Among 11,286 invited HCWs, 3075 valid responses were received, giving an overall response rate of 27.2%. Mean OLBI scores were 2.38 and 2.50 for Disengagement and Exhaustion, respectively. Burnout thresholds in Disengagement and Exhaustion were met by 79.7% and 75.3% of respondents, respectively. On multivariate regression analysis, Chinese or Malay ethnicity, HADS anxiety or depression scores ≥8, shifts lasting ≥8 hours, and being redeployed were significantly associated with higher OLBI mean scores, whereas high SAQ scores were significantly associated with lower scores. Among redeployed HCWs, those redeployed to high-risk areas in a different facility (offsite) had lower burnout scores than those redeployed within their own work facility (onsite). A higher proportion of HCWs redeployed offsite assessed their training to be good or better compared with those redeployed onsite.  **Conclusions and implications**: Every level of the health care workforce is susceptible to high levels of burnout during this pandemic. Modifiable workplace factors include adequate training, avoiding prolonged shifts ≥8 hours, and promoting safe working environments. Mitigating strategies should target every level of the health care workforce, including frontline and nonfrontline staff. Addressing and ameliorating burnout among HCWs should be a key priority for the sustainment of efforts to care for patients in the face of a prolonged pandemic. |
| 110 | A.-F. Q. Dijxhoorn, L. Brom, Y.M. van der Linden, C. Leget, and N.J. Raijmakers | Prevalence of burnout in healthcare professionals providing palliative care and the effect of interventions to reduce symptoms: A systematic literature review | 2021 | Palliat Med | **Background:** In recent years there has been increasing attention for the prevalence and prevention of burnout among healthcare professionals. There is unclarity about prevalence of burnout in healthcare professionals providing palliative care and little is known about effective interventions in this area.  **Aim:** To investigate the prevalence of (symptoms of) burnout in healthcare professionals providing palliative care and what interventions may reduce symptoms of burnout in this population.  **Design:** A systematic literature review based on criteria of the PRISMA statement was performed on prevalence of burnout in healthcare professionals providing palliative care and interventions aimed at preventing burnout.  **Data sources:** PubMed, PsycINFO and CINAHL were searched for studies published from 2008 to 2020. Quality of the studies was assessed using the method of Hawkers for systematically reviewing research.  **Results:** In total 59 studies were included. Burnout among healthcare professionals providing palliative care ranged from 3% to 66%. No major differences in prevalence were found between nurses and physicians. Healthcare professionals providing palliative care in general settings experience more symptoms of burnout than those in specialised palliative care settings. Ten studies reported on the effects of interventions aimed at preventing burnout. Reduction of one or more symptoms of burnout after the intervention was reported in six studies which were aimed at learning meditation, improving communication skills, peer-coaching and art-therapy based supervision.  **Conclusion:** The range of burnout among healthcare professionals providing palliative care varies widely. Interventions based on meditation, communication training, peer-coaching and art-therapy based supervision have positive effects but long-term outcomes are not known yet. |
| 111 | Y.H. Teo, T.Y. Peh, A. Abdurrahman, A. Lee, M. Chiam, W. Fong, et al. | A modified delphi approach to enhance nurturing of professionalism in postgraduate medical education in Singapore | 2021 | Singapore Med J | **Introduction:** Nurturing professional identities instils behavioural standards of physicians, in turn facilitating consistent professional attitudes, practice, and patient care. Identities are socioculturally constructed efforts, thus we must account for the social, cultural, and local healthcare factors that shape physicians' roles, responsibilities and expectations. This study aims to forward a program to nurture professionalism amongst physicians in Singapore.  **Methods:** A 3-phased-evidenced-based-approach was used. First, a systematic scoping review (SSR) was conducted to identify professionalism elements. Second, a questionnaire was created drawing from the SS's findings. Third, a modified Delphi involving local experts identifying socioculturally appropriate elements to nurture professionalism was conducted.  **Results:** The 124 included articles in the SSR revealed definitions, knowledge, skills, and approaches to nurturing professionalism. The modified Delphi identified professional traits, virtues, communication, ethical, self-care, teaching and assessment methods, and support mechanisms.  **Conclusion:** Results formed the basis to a holistic and longitudinal program focused on instilling professional traits and competencies over time through personalised and holistic support of physicians. Findings will be of interest to medical communities in the region and beyond. |
| 112 | F. Kilbertus, R. Ajjawi, and D.B. Archibald | You’re Not Trying to Save Somebody From Death: Learning as “Becoming” in Palliative Care | 2018 | Acad Med | **Purpose:** Learning can be conceptualized as a process of "becoming," considering individuals, workplace participation, and professional identity formation. How postgraduate trainees learn palliative care, encompassing technical competence, compassion, and empathy, is not well understood or explained by common conceptualizations of learning as "acquisition" and "participation." Learning palliative care, a practice that has been described as a cultural shift in medicine challenging the traditional role of curing and healing, provided the context to explore learning as "becoming."  **Method:** The authors undertook a qualitative narrative study, interviewing 14 residents from the University of Ottawa Family Medicine Residency Program eliciting narratives of memorable learning (NMLs) for palliative care. Forty-five NMLs were analyzed thematically. To illuminate the interplay among themes, an in-depth analysis of the NMLs was done that considered themes and linguistic and paralinguistic features of the narratives.  **Results:** Forty-five NMLs were analyzed. The context of NMLs was predominantly a variety of clinical workplaces during postgraduate training. Themes clustered around the concept of palliative care and how it contrasted with other clinical experiences, the emotional impact on narrators, and how learning happened in the workplace. Participants had expectations about their identities as doctors that were challenged within their NMLs for palliative care.  **Conclusions:** NMLs for palliative care were a complex entanglement of individual experience and social and workplace cultures highlighting the limitations of the "acquisition" and "participation" metaphors of learning. By conceptualizing learning as "becoming," what occurs during memorable learning can be made accessible to those supporting learners and their professional identity formation. |
| 113 | B. Burford | Group processes in medical education: learning from social identity theory | 2012 | Med Educ | **Context:** The clinical workplace in which doctors learn involves many social groups, including representatives of different professions, clinical specialties and workplace teams. This paper suggests that medical education research does not currently take full account of the effects of group membership, and describes a theoretical approach from social psychology, the social identity approach, which allows those effects to be explored.  **Methods:** The social identity approach has a long history in social psychology and provides an integrated account of group processes, from the adoption of group identity through a process of self-categorisation, to the biases and conflicts between groups. This paper outlines key elements of this theoretical approach and illustrates their relevance to medical education.  **Results:** The relevance of the social identity approach is illustrated with reference to a number of areas of medical education. The paper shows how research questions in medical education may be usefully reframed in terms of social identity in ways that allow a deeper exploration of the psychological processes involved. Professional identity and professionalism may be viewed in terms of self-categorisation rather than simply attainment; the salience of different identities may be considered as influences on teamwork and interprofessional learning, and issues in communication and assessment may be considered in terms of intergroup biases.  **Conclusions:** Social identity theory provides a powerful framework with which to consider many areas of medical education. It allows disparate influences on, and consequences of, group membership to be considered as part of an integrated system, and allows assumptions, such as about the nature of professional identity and interprofessional tensions, to be made explicit in the design of research studies. This power to question assumptions and develop deeper and more meaningful research questions may be increasingly relevant as the nature and role of the medical profession change. |
| 114 | A.P. Sawatsky, H. C. Nordhues, S.P. Merry, M.U. Bashir, and F.W. Hafferty | Transformative Learning and Professional Identity Formation During International Health Electives: A Qualitative Study Using Grounded Theory | 2018 | Acad Med | **Purpose:** International health electives (IHEs) are widely available during residency and provide unique experiences for trainees. Theoretical models of professional identity formation and transformative learning may provide insight into residents' experiences during IHEs. The purpose of this study was to explore transformative learning and professional identity formation during resident IHEs and characterize the relationship between transformative learning and professional identity formation.  **Method:** The authors used a constructivist grounded theory approach, with the sensitizing concepts of transformative learning and professional identity formation to analyze narrative reflective reports of residents' IHEs. The Mayo International Health Program supports residents from all specialties across three Mayo Clinic sites. In 2015, the authors collected narrative reflective reports from 377 IHE participants dating from 2001 to 2014. Reflections were coded and themes were organized into a model for transformative learning during IHEs, focusing on professional identity.  **Results:** Five components of transformative learning were identified during IHEs: a disorienting experience; an emotional response; critical reflection; perspective change; and a commitment to future action. Within the component of critical reflection, three domains relating to professional identity were identified: making a difference; the doctor-patient relationship; and medicine in its "purest form." Transformation was demonstrated through perspective change and a commitment to future action, including continued service, education, and development.  **Conclusions:** IHEs provide rich experiences for transformative learning and professional identity formation. Understanding the components of transformative learning may provide insight into the interaction between learner, experiences, and the influence of mentors in the process of professional identity formation. |
| 115 | J. Soo, P. Brett-MacLean, M.-T. Cave, and A. Oswald | At the precipice: a prospective exploration of medical students’ expectations of the pre-clerkship to clerkship transition | 2016 | Adv Health Sci Educ Theory Pract | **Abstract:** Medical learners face many challenging transitions. We prospectively explored students' perceptions of their upcoming transition to clerkship and their future professional selves. In 2013, 160/165 end-of-second-year medical students wrote narrative reflections and 79/165 completed a questionnaire on their perceptions of their upcoming transition to clerkship. Narratives were separately analyzed by four authors and then discussed to identify a final thematic framework using parsimonious category construction. We identified two overarching themes: (1) "Looking back": experiences which had helped students feel prepared for clerkship with subthemes focused on of patient care, shadowing, classroom teaching and the pre-clerkship years as foundational knowledge, (2) "Looking forward": anticipating the clerkship experience and the journey of becoming a physician with subthemes focused on death and dying, hierarchy, work-life balance, interactions with patients, concerns about competency and career choice. Questionnaire data revealed incongruities around expectations of minimal exposure to death and dying, little need for independent study and limited direct patient responsibility. We confirmed that internal transformations are happening in contemplative time even before clerkship. By prospectively exploring pre-clerkship students' perceptions of the transition to clerkship training we identified expectations and misconceptions that could be addressed with future curricular interventions. While students are aware of and anticipating their learning needs it is not as clear that they realise how much their future learning will depend on their own inner resources. We suggest that more attention be paid to professional identity formation and the development of the physician as a person during these critical transitions. |
| 116 | S.J. Hamstra, S.I. Woodrow, and R. S. Mangrulkar | Feeling pressure to stay late: socialisation and professional identity formation in graduate medical education | 2008 | Med Educ | Residency can be thought of as a process of socialisation whereby members learn how to behave as part of a culture. Identification with a group is reinforced by establishing common boundaries of behaviour and expectations. For example, the perception of ‘assaults on autonomy’ described in the target article served to change the residents’ behaviour towards an external group (in this case, the patients). Even within the medical profession, distinct sub-cultures may exist. For example, surgery residents perceive themselves as being more resilient to the effects of sleep deprivation than their peers in internal medicine and psychiatry, despite evidence to the contrary. This misperception may result from a surgical sub-culture in which staying late for the sake of patient care is highly valued and reinforced.3 Broader cultural factors may also influence this process of socialisation. In Sweden, for example, a 40-hour working week has been widely accepted in medical training for more than 30 years. In the UK, however, acceptance of the European Working Time Directive has not been entirely smooth and strong concerns have been expressed regarding the reduction in cases during training. Although working hour restrictions are now in effect in most western countries, they vary considerably, as do the health care systems in which they exist. What is needed now is an explicit recognition of leadership and teamwork in the curriculum.  The pressures to stay late may have changed since the imposition of duty hour restrictions in training programmes, but the process of professional identity formation remains. Amidst all the conflicting messages, residents who put in the extra mile may continue to feel less fulfilled, run the risk of burning out, and, consequently, become less engaged with their patients. In response to changes in the process of professional identity formation that have come about from managed care and duty hour restrictions, what is needed now is an explicit recognition of leadership and teamwork in the curriculum. |
| 117 | I. Wilson, L.S. Cowin, M. Johnson, and H. Young | Professional Identity in Medical Students: Pedagogical Challenges to Medical Education | 2013 | Teach Learn Med | **Background:** Professional identity, or how a doctor thinks of himself or herself as a doctor, is considered to be as critical to medical education as the acquisition of skills and knowledge relevant to patient care.  **Summary:** This article examines contemporary literature on the development of professional identity within medicine. Relevant theories of identity construction are explored and their application to medical education and pedagogical approaches to enhancing students' professional identity are proposed. The influence of communities of practice, role models, and narrative reflection within curricula are examined.  **Conclusions**: Medical education needs to be responsive to changes in professional identity being generated from factors within medical student experiences and within contemporary society. |
| 118 | H.S. Wald, J. White, S.P. Reis, A.Y. Esquibel, and D. Anthony | Grappling with complexity: Medical students’ reflective writings about challenging patient encounters as a window into professional identity formation | 2019 | Med Teach | **Aim:** Clerkship-specific interactive reflective writing (IRW)-enhanced reflection may enhance professional identity formation (PIF), a fundamental goal of medical education. PIF process as revealed in students? reflective writing (RW) has been understudied.  **Methods:** The authors developed an IRW curriculum within a Family Medicine Clerkship (FMC) and analyzed students? reflections about challenging/difficult patient encounters using immersion-crystallization qualitative analysis.  **Results:** The qualitative analysis identified 26 unique emergent themes and five distinct thematic categories (1. Role of emotions, 2. Role of cognition, 3. Behaviorally responding to situational context, 4. Patient factors, and 5. External factors) as well as an emergent PIF model from a directed content analysis. The model describes students’ backgrounds, emotions and previous experiences in medicine merging with external factors and processed during student-patient interactions. The RWs also revealed that processing often involves polarities (e.g. empathy/lack of empathy or encouragement/disillusionment) as well as dissonance between idealized visions and lived reality.  **Conclusions:** IRW facilitates and ideally supports grappling with the lived reality of medicine; uncovering a "positive hidden curriculum" within medical education. The authors propose engaging learners in guided critical reflection about complex experiences for meaning-making within a safe learning climate as a valuable way to cultivate reflective, resilient professionals with "prepared" minds and hearts for inevitable challenges of healthcare practice. |
| 119 | R. Matthews, K. Smith-Han, and H. Nicholson | From physiotherapy to the army: negotiating previously developed professional identities in mature medical students | 2020 | Adv Health Sci Educ Theory Pract | **Background:** Professional identity formation (PIF) is a life-long process, starting even before professional education. High levels of motivation for medical school are essential for effective learning and academic success. Both are key factors in future physicians’ professional and personal development, and according to self-determination theory, professional identity (PI) and students’ levels of motivation could be closely linked. Therefore, we sought to investigate whether PI and strength of motivation for medical school are associated in new medical students.  **Methods:** In a cross-sectional survey, all new medical students in Munich, Germany, were asked to complete the Macleod Clark Professional Identity Scale (MCPIS-9) and the Strength of Motivation for Medical School-Revised questionnaire (SMMS-R) as well as to provide information about age, gender, and waiting time before starting medical school.  **Results:** Eight hundred eleven out of 918 new medical students participated in the survey. A positive correlation between the MCPIS-9 and the SMMS-R (p < 0.001) was found. Female students showed higher scores in the SMMS-R (p < 0.05) and the SMMS-R-subscale Readiness to Start (p < 0.001). The amount of waiting semesters showed a positive correlation with the total SMMS-R score (p < 0.01) as well as with the subscales Readiness to Start and Persistence (both p < 0.001).  **Discussion:** We found an association between PI and strength of motivation for medical school in a large cohort of new medical students. Female gender and more waiting semesters were associated with higher levels of self-perceived motivation and higher scores on the SMMS-R-subscale Readiness to Start. More research is needed to better understand this topic to further improve medical education. |
| 120 | E. Stuart, D. O'Leary, R. Rowntree, C. Carey, L. O'Rourke, E. O'Brien, et al. | Challenges in experiential learning during transition to clinical practice: A comparative analysis of reflective writing assignments during general practice, paediatrics and psychiatry clerkships | 2020 | Med Teach | **Introduction**: This study explored the reflective writing (RW) of senior medical students across a co-ordinated reflection education programme in General Practice, Paediatrics and Psychiatry clerkships during their transition to clinical clerkships. The study compared RW themes from within and across three clerkships in order to understand the influence clerkships had on experiential learning and developing professional identity.  **Methods:** All medical students in their penultimate year were invited to participate in the study. 135 reflection assignments were analysed. A qualitative thematic analysis of students' RW was performed. An inductive approach was used and data saturation was achieved.  **Results:** Clerkship specific themes were the intimacy of the experience in General Practice, the powerlessness students felt and the challenge of delivering family centred care in Paediatrics and the sense of perceived risk in Psychiatry. Common themes across the three clerkships were of emotional struggles in developing a professional identity.  **Conclusion:** There is an educational need for developmental space for students during General Practice, greater focus on preparing students for relationship building during Paediatrics and addressing stigma and personal safety issues in students during the Psychiatry clerkships. Across clerkships there is a need for better use of evidence based pedagogies to support emotional development. |
| 121 | S. Rosenthal, B. Howard, Y.R. Schlussel, D. Herrigel, B.G. Smolarz, B. Gable, et al. | Humanism at heart: preserving empathy in third-year medical students | 2011 | Acad Med | **Purpose:** Research suggests that medical student empathy erodes during undergraduate medical education. The authors evaluated the Jefferson Scale of Physician Empathy Medical Student Version (JSPE-MS) scores of two consecutive medical school classes to assess the impact of an educational intervention on the preservation of empathy.  **Method:** The authors conducted a before-and-after study of 209 Robert Wood Johnson Medical School (RWJMS) students enrolled in the classes of 2009 and 2010. Students' clerkships included a mandatory, longitudinal "Humanism and Professionalism" (H&P) component, which included blogging about clerkship experiences, debriefing after significant events, and discussing journal articles, fiction, and film. Students completed the JSPE-MS during their first and last clerkships.  **Results:** The results showed that (1) contrary to previous studies' findings, third-year students did not show significant decline in empathy as measured by the JSPE-MS (these students, from two consecutive RWJMS classes, experienced the H&P intervention), (2) students selected for the Gold Humanism Honor Society (GHHS) were significantly different from their peers in empathy scores as measured by JSPE-MS, and (3) knowledge of selection for the GHHS seems to positively influence students' JSPE-MS scores.  **Conclusions:** Maintaining empathy during the third year of medical school is possible through educational intervention. A curriculum that includes safe, protected time for third-year students to discuss their reactions to patient care situations during clerkships may have contributed to the preservation of empathy. Programs designed to validate humanism in medicine (such as the GHHS) may reverse the decline in empathy as measured by the JSPE-MS. |
| 122 | S.M. Wright, R.B. Levine, B. Beasley, P. Haidet, T.W. Gress, S. Caccamese, et al. | Personal growth and its correlates during residency training | 2006 | Med Educ | To explore the characteristics of and factors associated with personal growth during residency training. In 2003, 359 house officers on 7 internal medicine residency training programmes in the USA were surveyed about their training experiences and issues related to their personal growth. Factor analysis and internal reliability testing were used to develop a 'personal growth scale'. Logistic regression models were then used to identify independent associations between individual variables and 'high' versus 'low' personal growth scores. A total of 281 house officers (80%) responded. The personal growth scale had a Cronbach's alpha of 0.81. Factors that were independently associated with achieving high amounts of personal growth during residency training included: agreeing that reflection is important during residency training (odds ratio [OR] 2.9, 95% confidence interval [CI] 1.1-7.4); being male (OR 2.6, 95% CI 1.4-4.5); being non-white (OR 2.2, 95% CI 1.3-3.9); having a strong desire to develop personally and professionally (OR 2.2, 95% CI 1.1-4.1), and feeling highly supported by one's programme director (OR 2.1, 95% CI 1.2-3.9). Independent predictors of scoring below the median on the personal growth scale included feeling emotionally isolated at work (OR 0.4, 95% CI 0.2-0.7) and noting that negative or disappointing experiences had been powerful (OR 0.4, 95% CI 0.2-0.9). Disparate amounts of personal growth occur among trainees during residency training. Residency programmes interested in promoting personal growth among their trainees may wish to focus on modifiable factors that are associated with personal growth, such as fostering supportive relationships and encouraging reflection. |
| 123 | R. B. Levine, P. Haidet, D.E. Kern, B.W. Beasley, L. Bensinger, D.W. Brady, et al. | Personal growth during internship | 2006 | J Gen Intern Med | **Background:** During clinical training, house officers frequently encounter intense experiences that may affect their personal growth. The purpose of this study was to explore processes related to personal growth during internship.  **Design:** Prospective qualitative study conducted over the course of internship.  **Participants:** Thirty-two postgraduate year (PGY)-1 residents from 9 U.S. internal medicine training programs.  **Approach:** Every 8 weeks, interns responded by e-mail to an open-ended question related to personal growth. Content analysis methods were used to analyze the interns’ writings to identify triggers, facilitators, and barriers related to personal growth.  **Results:** Triggers for personal growth included caring for critically ill or dying patients, receiving feedback, witnessing unprofessional behavior, experiencing personal problems, and dealing with the increased responsibility of internship. Facilitators of personal growth included supportive relationships, reflection, and commitment to core values. Fatigue, lack of personal time, and overwhelming work were barriers to personal growth. The balance between facilitators and barriers may dictate the extent to which personal growth occurs.  **Conclusions:** Efforts to support personal growth during residency training include fostering supportive relationships, encouraging reflection, and recognizing interns’ core values especially in association with powerful triggers |
| 124 | M.A. Fischer, H.-L. Haley, C.L. Saarinen, and K.C. Chretien | Comparison of blogged and written reflections in two medicine clerkships | 2011 | Med Educ | **Context:** Academic medical centres may adopt new learning technologies with little data on their effectiveness or on how they compare with traditional methodologies. We conducted a comparative study of student reflective writings produced using either an electronic (blog) format or a traditional written (essay) format to assess differences in content, depth of reflection and student preference.  **Methods**: students in internal medicine clerkships at two US medical schools during the 2008-2009 academic year were quasi-randomly assigned to one of two study arms according to which they were asked to either write a traditional reflective essay and subsequently join in faculty-moderated, small-group discussion (n = 45), or post two writings to a faculty-moderated group blog and provide at least one comment on a peer's posts (n = 50). Examples from a pilot block were used to refine coding methods and determine inter-rater reliability. Writings were coded for theme and level of reflection by two blinded authors; these coding processes reached inter-rater reliabilities of 91% and 80%, respectively. Anonymous pre- and post-clerkship surveys assessed student perceptions and preferences.  **Results:** Student writing addressed seven main themes: (i) being humanistic; (ii) professional behaviour; (iii) understanding caregiving relationships; (iv) being a student; (v) clinical learning; (vi) dealing with death and dying, and (vii) the health care system, quality, safety and public health. The distribution of themes was similar across institutions and study arms. The level of reflection did not differ between study arms. Post-clerkship surveys showed that student preferences for blogging or essay writing were predicted by experience, with the majority favouring the method they had used.  **Conclusions:** Our study suggests there is no significant difference in themes addressed or levels of reflection achieved when students complete a similar assignment via online blogging or traditional essay writing. Given this, faculty staff should feel comfortable in utilising the blog format for reflective exercises. Faculty members could consider the option of using either format to address different learning styles of students. |
| 125 | D.E. Kern, S.M. Wright, J.A. Carrese, M. Lipkin, Jr., J.M. Simmons, D.H. Novack, et al. | Personal growth in medical faculty: a qualitative study | 2001 | West J Med | **Background:** A physician's effectiveness depends on good communication, and cognitive and technical skills used with wisdom, compassion, and integrity. Attaining the last attributes requires growth in awareness and management of one's feelings, attitudes, beliefs, and life experiences. Yet, little empiric research has been done on physicians' personal growth.  **Objective:** To use qualitative methods to understand personal growth in a selected group of medical faculty.  **Design:** Case study, using open-ended survey methods to elicit written descriptions of respondents' personal growth experiences.  **Setting:** United States and Great Britain.  **Participants:** Facilitators, facilitators-in-training, and members of a personal growth interest group of the American Academy on Physician and Patient, chosen because of their interest, knowledge, and experience in the topic area and their accessibility.  **Measurements**: Qualitative analysis of submitted stories included initially identifying and sorting themes, placing themes into categories, applying the categories to the database for verification, and verifying findings by independent reviewers.  **Results:** Of 64 subjects, 32 returned questionnaires containing 42 stories. Respondents and nonrespondents were not significantly different in age, sex, or specialty. The analysis revealed 3 major processes that promoted personal growth: powerful experiences, helping relationships, and introspection. Usually personal growth stories began with a powerful experience or a helping relationship (or both), proceeded to introspection, and ended in a personal growth outcome. Personal growth outcomes included changes in values, goals, or direction; healthier behaviors; improved connectedness with others; improved sense of self; and increased productivity, energy, or creativity.  **Conclusions**: Powerful experiences, helping relationships, and introspection preceded important personal growth. These findings are consistent with theoretic and empiric adult learning literature and could have implications for medical education and practice. They need to be confirmed in other physician populations. |
| 126 | R. Kimmons and G. Veletsianos | The fragmented educator 2.0: Social networking sites, acceptable identity fragments, and the identity constellation | 2014 | Comput Educ | Social networking sites (SNS) have been used to support educational and professional endeavors. However, little research has been done to understand the relationship between educator identity and participation in SNS or to examine the implications that institutional regulation of such media may have upon educator identity.  Using grounded theory, in this study we developed a framework for understanding how a group of teacher education students viewed their developing identities within social networking sites as they began the life transition to becoming educators. The theory that emerged from this study proposes that educator identity consists of a constellation of interconnected acceptable identity fragments, which are each intentional, authentic, transitional, necessarily incomplete, and socially-constructed and -responsive. This view of educator identity contrasts sharply with previous views of identity by highlighting the complicated, negotiated, and recursive relationship that exists between educator participation in SNS and educator identity. Additionally, this perspective suggests that educator participation in SNS is neither fully representative of authentic identity (as prominent SNS models imply) nor dramaturgical.  These findings yield important implications for educators, researchers, educational institutions, lawmakers, and SNS developers alike, because they lead to a more sophisticated understanding of identity and online participation that is essential for developing mechanisms to support moral and legal judgments, professionalism, and social interactions relative to SNS. |
| 127 | M. J. Gosselink | Medical weblogs: advocacy for positive cyber role models | 2011 | Clin Teach | **Background:** The development of empathy and of medical professionalism is important in medical education. Research has shown a decline in empathy during medical study. An important factor that may contribute to this decline is the lack of positive role models. Students identify positive and negative role models in the clinical ward and in classrooms. Positive clinical role models, showing good professionalism, will foster the student's professional growth.  **Context:** Students base both knowledge and opinions on information they find on the internet, such as 'medical weblogs'. These weblogs may be regarded as a new aspect of the 'informal curriculum', even though they might be written by 'negative role models'. In our experience, students frequently require debriefing of the undifferentiated, frequently disrespectful, grotesque or unprofessional approaches they encounter in general, and in this aspect online in particular.  **Innovation:** When written by appropriate professionals, medical weblogs can provide a unique opportunity for the medical student to follow the writings of positive role models. Would it be a worthwhile investment if globally distributed medical professionals from different specialties put time and effort into writing weblogs on their daily practice and reflections? These weblogs could be assembled on one international educational website, labelled as 'professionally approved' and made easily accessible. Medical students internationally would have access to the daily practice of medical professionals who are excellent positive role models. They could also be encouraged to discuss issues with them. We hypothesise that witnessing the encounters of these globally distributed doctors, and following their reflections, may add to the growth of empathy and professionalism in students. |
| 128 | C. Fieseler, M. Meckel, and G. Ranzini | Professional Personae - How Organizational Identification Shapes Online Identity in the Workplace | 2014 | J Comput Mediat Commun | As organizations become increasingly mediatized, the roles of professionals are reshaped and negotiated, and the boundaries between professional and private relationships are blurred. In this context, the extent to which one identifies with his or her organization might play an important role. This paper investigates how professionals construct their digital identities on social media sites, focusing in particular on their willingness to overlap private and work profiles to create a univocal online persona. Based on a sample of 679 communication and marketing managers, the paper analyzes the self-representational choices of professionals and demonstrates how organizational identification influences professionals' tendency to combine their domains under one online persona, and their confidence to use social media in a professional context. |
| 129 | J. Stokes and B. Price | Social Media, Visual Culture and Contemporary Identity | 2017 | Open Cybern Syst J | In developed nations, university students lead a rich digital life which includes maintaining connections through image-based social networks such as Instagram, Snapchat and Tinder. These ubiquitous technologies are shaping student identities and social practices in an ongoing manner. Social media is used to construct identity through visual branding, wherein individuals utilise imagery designed to attract followers and maintain careful curation of an online persona. In this digital economy, social capital is attached to attributes such as trustworthiness, authenticity and attractiveness. Regular social media use assists in the development of sophisticated visual practices, through which the everyday user shifts their own representation. Network members draw upon increased compositional knowledge and digital tools to depict themselves in a flattering manner which conveys a positive message about their identity as brand. Attractiveness and creativity become dominant factors in these online fora, whereas the widespread use of image editing tools draws trustworthiness and authenticity factors into question. This paper uses Social Learning Theory to explore the use of social media for identity construction, identifying issues inherent for students who place themselves in constant comparison to a wide range of peers, and ways in which educators can utilise these perspectives to inform teaching. |
| 130 | R.O. Maghrabi, R.L. Oakley, and H.R. Nemati | The impact of self-selected identity on productive or perverse social capital in social network sites | 2014 | Comput Human Behav | Social network sites can provide a person with the freedom to represent themselves in various ways, thus exhibiting multiple variations of their identity. Research states that an individual’s identity is self- monitored depending on the contextual situation that they are in. The type of social capital that one derives from social network sites can be impacted by this self-monitoring ability. Current research has addressed how productive social capital can be gained in social network sites. However, limited research has addressed the issue of perverse social capital, especially in social network sites. We argue that social network sites are a particularly unique environment that can affect an individual’s representation of their identity, thus increasing the likelihood of producing perverse social capital. We examine how technology affects an individual’s selected self-identity, as measured through their self-monitoring ability, and how this altered behavior leads to productive or perverse social capital in social network sites. |
| 131 | M. Hojat, M.J. Vergare, K. Maxwell, G. Brainard, S.K. Herrine, G.A. Isenberg, et al. | The Devil is in the Third Year: A Longitudinal Study of Erosion of Empathy in Medical School | 2009 | Acad Med | **Purpose:** This longitudinal study was designed to examine changes in medical students' empathy during medical school and to determine when the most significant changes occur.  **Method:** Four hundred fifty-six students who entered Jefferson Medical College in 2002 (n = 227) and 2004 (n = 229) completed the Jefferson Scale of Physician Empathy at five different times: at entry into medical school on orientation day and subsequently at the end of each academic year. Statistical analyses were performed for the entire cohort, as well as for the "matched" cohort (participants who identified themselves at all five test administrations) and the "unmatched" cohort (participants who did not identify themselves in all five test administrations).  **Results:** Statistical analyses showed that empathy scores did not change significantly during the first two years of medical school. However, a significant decline in empathy scores was observed at the end of the third year which persisted until graduation. Findings were similar for the matched cohort (n = 121) and for the rest of the sample (unmatched cohort, n = 335). Patterns of decline in empathy scores were similar for men and women and across specialties.  **Conclusions:** It is concluded that a significant decline in empathy occurs during the third year of medical school. It is ironic that the erosion of empathy occurs during a time when the curriculum is shifting toward patient-care activities; this is when empathy is most essential. Implications for retaining and enhancing empathy are discussed. |
| 132 | B.W. Newton, L. Barber, J. Clardy, E. Cleveland, and P. O'Sullivan | Is There Hardening of the Heart During Medical School? | 2008 | Acad Med | **Purpose:** To determine whether vicarious empathy (i.e., to have a visceral empathic response, versus role-playing empathy) decreases, and whether students choosing specialties with greater patient contact maintain vicarious empathy better than do students choosing specialties with less patient contact.  **Method:** The Balanced Emotional Empathy Scale was administered at the beginning of each academic year at the University of Arkansas for Medical Sciences for four classes, 2001-2004. Students also reported their gender and specialty choice. Specialty choice was classified as core (internal medicine, family medicine, obstetrics-gynecology, pediatrics, and psychiatry) or noncore (all other specialties).  **Results:** Vicarious empathy significantly decreased during medical education (P < .001), especially after the first and third years. Students choosing core careers had higher empathy than did those choosing noncore careers. Men choosing core careers initially had empathy exceeding population norms, but their empathy fell to be comparable with that of norms by the end of their third year. The empathy of men choosing noncore careers was comparable with that of norms. Women choosing core careers had empathy scores comparable with those of norms, but the scores of women choosing noncore careers fell below those of the norms by their second year.  **Conclusions:** The findings suggest that undergraduate medical education may be a major determinant differentially affecting the vicarious empathy of students on the basis of gender and/or specialty choice. The greatest impact occurred in men who chose noncore specialties. The significant decrease in vicarious empathy is of concern, because empathy is crucial for a successful physician-patient relationship. |
| 133 | J.M. Kaczmarczyk, A. Chuang, L. Dugoff, J.F. Abbott, A.J. Cullimore, J. Dalrymple, et al. | e-Professionalism: A New Frontier in Medical Education | 2013 | Teach Learn Med | **Background:** This article, prepared by the Association of Professors of Gynecology and Obstetrics Undergraduate Medical Education Committee, discusses the evolving challenges facing medical educators posed by social media and a new form of professionalism that has been termed e-professionalism.  **Summary:** E-professionalism is defined as the attitudes and behaviors that reflect traditional professionalism paradigms but are manifested through digital media. One of the major functions of medical education is professional identity formation; e-professionalism is an essential and increasingly important element of professional identity formation, because the consequences of violations of e-professionalism have escalated from academic sanctions to revocation of licensure.  **Conclusion:** E-professionalism should be included in the definition, teaching, and evaluation of medical professionalism. Curricula should include a positive approach for the proper professional use of social media for learners. |
| 134 | J. Brown, H. Reid, T. Dornan, and D. Nestel | Becoming a clinician: Trainee identity formation within the general practice supervisory relationship | 2020 | Med Educ | **Objectives:** Becoming a clinician is a trajectory of identity formation in the context of supervised practice. This is a social process where the supervisory relationship is key. Therefore, to know how to support identity formation of clinical trainees, it is necessary to understand how this happens within the supervisory relationship. Our aim was to develop a conceptualisation of trainee identity formation within the general practice supervisory relationship to aid its support.  **Methods:** We took a critical realist approach using case study design and 'cultural worlds' theory as a conceptual frame. Each case comprised a general practice trainee and supervisor pair. Our data were weekly audiorecordings of interactions between trainee, supervisor and a patient over 12 weeks augmented by post-interaction reflections and sequential interviews. We undertook interpretive analysis using dialogic methods focusing on the doing of language and the cultural discourses expressed.  **Results:** We identified three social discourses centring on: clinical responsibility; ownership of clinical knowledge, and measures of trainee competency. Versions of these discourses defined four trainee-supervisor relational arrangements within which trainee and supervisor assumed reciprocal identities. We labelled these: junior learner and expert clinician; apprentice assistant and master coach, and lead clinician and advisor. We found a trajectory across these identity arrangements. Behind this trajectory was an invitation by the supervisor to the trainee into the social space of clinician and a readiness of the trainee to accept this invitation. Congruence in supervisor and trainee positioning was important.  **Conclusions:** In the supervisory relationship, trainee and supervisor adopted reciprocal identities. For trainees to progress to identity of 'lead clinician,' supervisors needed to invite their trainee into this space and vacate it themselves. Congruence between supervisor positioning of their trainee and trainee authorship of themselves was important and was aided by explicit dialogue and common purpose. We offer a model and language for trainees, supervisors and departments or schools to facilitate this. |
| 135 | B.L. Henschen, J.A. Bierman, D.B. Wayne, E.R. Ryan, J.X. Thomas, R.H. Curry, et al. | Four-Year Educational and Patient Care Outcomes of a Team-Based Primary Care Longitudinal Clerkship | 2015 | Acad Med | **Background:** Longitudinal clerkships show promise in improving undergraduate primary care education. This study examines the Education-Centered Medical Home (ECMH), a longitudinal clerkship embedding teams of students across all four years into primary care clinics to provide patient care and serve as health coaches for high-risk patients.  **Method:** All students graduating in 2015 were surveyed to assess attitudes, experiences, and preferences regarding primary care education. ECMH students were compared with students receiving their primary care training in a traditional curriculum (TC) using paired measures of comparison. To assess the impact of the ECMH on patient care quality, authors performed a detailed chart review at one site.  **Results:** Seventy-six percent of eligible students participated in the study. ECMH students (n = 69) and TC students (n = 68) had similar baseline academic performance and career interests. ECMH students reported more continuity-of-care experiences, higher satisfaction with their primary care learning climate (86% versus 61% in the EMCH and TC cohorts, respectively), more confidence in their quality improvement skills, and scored higher on measures of perceived patient centeredness. Students from both groups recommended the ECMH (91% and 57%, respectively). Student involvement at one ECMH site was correlated with increased patient contacts and improved delivery of recommended preventive care.  **Conclusions:** Incorporating students longitudinally into primary care clinics is highly rated by students. The ECMH model led to improved continuity, improved perceptions of the learning climate, and higher patient centeredness. Preliminary data suggest that students add value and improve patient outcomes during longitudinal clinical experiences. |
| 136 | J.E. Lim-Dunham, D.C. Ensminger, J.A. McNulty, A.E. Hoyt, and A.J. Chandrasekhar | A Vertically Integrated Online Radiology Curriculum Developed as a Cognitive Apprenticeship: Impact on Student Performance and Learning | 2016 | Acad Radiol | **Rationale and Objectives:** The principles of Collins’ cognitive apprenticeship model were used to design a radiology curriculum in which medical students practice radiological skills using online case-based modules. The modules are embedded within clinical third- year clerkships, and students are provided with personalized feedback from the instructors. We describe the development of the vertical online radiology curriculum and evaluate its impact on student achievement and learning process using a mixed method approach.    **Materials and Methods:** The curriculum was developed over a 2-year period. Student participation was voluntary in the first year and mandatory in the second year. For quantitative curriculum evaluation, student metrics for voluntary versus mandatory groups were assessed using independent sample t tests and variable entry method regression analysis. For qualitative analysis, responses from a survey of students about the value of the curriculum were organized into defined themes using consensus coding.  **Results:** Mandatory participation significantly improved (p = .001) the mean radiology examination score (82 %) compared to the voluntary group (73%), suggesting that mandatory participation had a beneficial effect on student performance. Potential preexisting differences in underlying general academic performance were accounted for by including mean basic science grades as the first variable in the regression model. The significant increase in R2 from .16 to .28 when number of radiology cases completed was added to the original model, and the greater value of the standardized beta for this variable, suggest that the curriculum made a significant contribution to students’ radiology examination scores beyond their baseline academic performance. Five dominant themes about curricular characteristics that enhanced student learning and beneficial outcomes emerged from consensus coding. These themes were (1) self-paced design, (2) receiving feedback from faculty, (3) clinical relevance of cases, (4) gaining confidence in interpreting radiological images, and (5) transfer of conceptual knowledge to actual practice.  **Conclusions:** The vertically integrated online radiology curriculum can positively impact student performance and learning process in the context of the cognitive apprenticeship model. |
| 137 | T.K. Sen Gupta, R.B. Muray, A. McDonell, B. Murphy, and A.D. Underhill | Rural internships for final year students: clinical experience, education and workforce | 2008 | Rural Remote Health | **Introduction:** The James Cook University School of Medicine is the only complete medical school in northern Australia, and it has a mission to prepare graduates to meet the unique needs of the region with a particular emphasis on rural, remote, Indigenous and tropical health. Eight-week 'rural internships' have been undertaken by all sixth-year medical students at James Cook University since 2005. Each student had previously completed at least 12 weeks of structured rural placements in years 2 and 4, as well as other core teaching in rural health including the year 2 subject, 'Rural, Remote, Indigenous and Tropical Health'. Students worked in rural hospitals across northern Australia developing and practising clinical skills under the supervision of senior staff. Students undertook full-time inpatient and outpatient responsibilities under supervision, being rostered for after-hours work with appropriate support. Assessment involved a learning portfolio, including multi-source feedback from peers, supervisors and patients, and a population health project and a telephone referral exercise.  **Methods:** This article describes the development, implementation and assessment of the first years of the program, from 2005 to 2007. Evaluation included student questionnaires, site visits and interviews, and follow-up teleconferences with preceptors.  **Results:** The rural internship provides senior medical students with valuable experience by active participation in the healthcare team. Students reported a rich and varied clinical experience. Students accept limited supervised responsibility and further their ability and confidence to undertake the role of the intern. Importantly, they proved not to be a burden to the system. This rotation therefore appears to meet educational needs without compromising the local workforce (and indeed may add to it). Students felt welcomed by their communities and enjoyed the social and cultural aspects of their attachment, as well as the clinical aspects and the opportunity to further their understanding of rural communities, rural health care and the healthcare team. Preparation of the students, the preceptors and the communities emerged as a key element of success.  **Conclusion:** This model extends and enhances the traditional apprenticeship model by its rural focus and distributed nature, and involvement of the entire student cohort. In addition, the contribution to patient care by senior students and junior doctors enables a consultant-registrar-resident model, in which experienced rural doctors function as consultants providing advice, support and tuition rather than predominantly face-to-face patient care. This approach also provides a means to address an emerging paradox: rural preceptors and communities want to teach students, appreciating the long-term workforce implications, but are increasingly constrained by resources, particularly time. Similar innovative approaches should be explored in other settings. |
| 138 | S. Yardley, P.W. Teunissen, and T. Dornan | Experiential learning: AMEE Guide No. 63 | 2012 | Med Teach | This Guide provides an overview of educational theory relevant to learning from experience. It considers experience gained in clinical workplaces from early medical student days through qualification to continuing professional development. Three key assumptions underpin the Guide: learning is 'situated'; it can be viewed either as an individual or a collective process; and the learning relevant to this Guide is triggered by authentic practice-based experiences. We first provide an overview of the guiding principles of experiential learning and significant historical contributions to its development as a theoretical perspective. We then discuss socio-cultural perspectives on experiential learning, highlighting their key tenets and drawing together common threads between theories. The second part of the Guide provides examples of learning from experience in practice to show how theoretical stances apply to clinical workplaces. Early experience, student clerkships and residency training are discussed in turn. We end with a summary of the current state of understanding. |
| 139 | B.L. Dickinson, K. Gibson, K. VanDerKolk, J. Greene, C.A. Rosu, D.D. Navedo, et al. | “It is this very knowledge that makes us doctors”: an applied thematic analysis of how medical students perceive the relevance of biomedical science knowledge to clinical medicine | 2020 | BMC Med Educ | **Background:** Intensive study of the biomedical sciences remains a core component of undergraduate medical education with medical students often completing up to 2 years of biomedical science training prior to entering clerkships. While it is generally accepted that biomedical science knowledge is essential for clinical practice because it forms the basis of clinical reasoning and decision-making, whether medical students perceive an expanded role for their biomedical science knowledge remains to be examined.  **Methods:** We conducted a qualitative research study to explore how medical students in the first clerkship year perceived the relevance of biomedical science knowledge to clinical medicine during this pivotal time as they begin their transition from students to physicians. To identify previously unidentified perspectives and insights, we asked students to write brief essays in response to the prompt: How is biomedical science knowledge relevant to clinical medicine? Ten codes and four themes were interpreted through an applied thematic analysis of students’ essays.  **Results:** Analysis of students’ essays revealed novel perspectives previously unidentified by survey studies and focus groups. Specifically, students perceived their biomedical science knowledge as contributory to the development of adaptive expertise and professional identity formation, both viewed as essential developmental milestones for medical students.  **Conclusions:** The results of this study have important implications for ongoing curricular reform efforts to improve the structure, content, delivery, and assessment of the undergraduate medical curriculum. Identifying the explicit and tacit elements of the formal, informal, and hidden curriculum that enable biomedical science knowledge to contribute to the development of adaptive expertise and professional identity formation will enable the purposeful design of innovations to support the acquisition of these critical educational outcomes. |
| 140 | S. Sarraf-Yazdi, Y.N. Teo, A.E.H. How, Y.H. Teo, S. Goh, C.S. Kow, et al. | A Scoping Review of Professional Identity Formation in Undergraduate Medical Education | **2021** | **J Gen Intern Med** | **Background:** Professional identity formation (PIF) in medical students is a multifactorial phenomenon, shaped by ways that clinical and non-clinical experiences, expectations and environmental factors merge with individual values, beliefs and obligations. The relationship between students' evolving professional identity and self-identity or personhood remains ill-defined, making it challenging for medical schools to support PIF systematically and strategically. Primarily, to capture prevailing literature on PIF in medical school education, and secondarily, to ascertain how PIF influences on medical students may be viewed through the lens of the ring theory of personhood (RToP) and to identify ways that medical schools support PIF.  **Methods:** A systematic scoping review was conducted using the systematic evidence-based approach. Articles published between 1 January 2000 and 1 July 2020 related to PIF in medical students were searched using PubMed, Embase, PsycINFO, ERIC and Scopus. Articles of all study designs (quantitative and qualitative), published or translated into English, were included. Concurrent thematic and directed content analyses were used to evaluate the data.  **Results:** A total of 10443 abstracts were identified, 272 full-text articles evaluated, and 76 articles included. Thematic and directed content analyses revealed similar themes and categories as follows: characteristics of PIF in relation to professionalism, role of socialization in PIF, PIF enablers and barriers, and medical school approaches to supporting PIF.  **Discussion:** PIF involves iterative construction, deconstruction and inculcation of professional beliefs, values and behaviours into a pre-existent identity. Through the lens of RToP, factors were elucidated that promote or hinder students' identity development on individual, relational or societal levels. If inadequately or inappropriately supported, enabling factors become barriers to PIF. Medical schools employ an all-encompassing approach to support PIF, illuminating the need for distinct and deliberate longitudinal monitoring and mentoring to foster students' balanced integration of personal and professional identities over time. |
| 141 | C. L. Alford and D. M. Currie | Introducing first-year medical students to clinical practice by having them "shadow" third-year clerks | **2004** | **Teach Learn Med** | **Background:** Reflection is a key element in learning from observation and experience of future profession’s roles and responsibilities in clinical encounters. Moreover, reflection helps students cope with the challenges, complexities, and uncertainties of professional development. Students’ written reflections on clinical exposure offer valuable information, and their analysis provides instructors with invaluable insight into students' experiences. This study evaluated Operating Room students’ written reflections on their first clinical exposure experiences towards their future profession through the shadowing program.  **Methods:** This study was a qualitative analysis on Operating Room freshmen’s reflections in the undergraduate program of Zahedan and Zabol University of Medical Sciences in Iran. After the shadowing program, all participants were asked to write an unstructured written reflection, and these fifty written reflections were de-identified and independently analyzed‏ using the thematic analysis approach.  **Results:** Qualitative analysis extracted 10 subthemes and four main themes including (i) Moving towards the guiding realities of future profession, (ii) Discovering milestones of realizing professional identity, (iii) Managing the emotions affecting the perception of future profession’s desirability, and (iv) Excellence in professional growth and development.  **Conclusion:** Reflecting on the experience of the shadowing program, the participants described being in the OR environment as a stimulating and valuable learning opportunity. Moreover, this experience helped improve their perception of future profession’s realities, as well as initiate realization of professional identity and planning for professional developments. |
| 142 | J. D. Boudreau, M. E. Macdonald and Y. Steinert | Affirming professional identities through an apprenticeship: insights from a four-year longitudinal case study | **2014** | **Acad Med** | **Purpose:** A four-year course, entitled Physician Apprenticeship, was introduced at McGill University's Faculty of Medicine in 2005. The primary objective of the course is to assist students in their transition from laymen to physicians. The goal of this study was to understand the apprenticeship learning process, particularly its contribution to professional identity formation.  **Method:** For data collection, the authors used a longitudinal case study design with mixed methods. They conducted the study over a four-year curricular cycle, from 2008-2009 to 2011-2012. The case consisted of three apprenticeship groups. Students (n = 24) and teachers (n = 3) represented two subgroups for data analysis.  **Results:** Physician Apprenticeship activities promoted and sustained medical professionalization in the participants. Salient features of successful apprenticeship learning were access to authentic clinical experiences as well as the provision of a safe learning environment and guided critical reflection. The latter two ingredients appear to be mutually reinforcing and contributed to the creation of meaningful student-teacher relationships. Teachers exhibited several qualities that align with a parental role. Students became increasingly aware of having entered the kinship of physicians. Teachers experienced a renewal and validation of their commitment to the ideals of medicine.  **Conclusions:** Findings strongly suggest that a longitudinal apprenticeship in an undergraduate medical program can contribute to the formation and reaffirmation of professional identity. The case study design permitted the authors to create a provisional conceptual model explicating important features of the apprenticeship learning process. |
| 143 | K.S. Peer | Professional Identity Formation: Considerations for Athletic Training Education | **2016** | Athl Train Educ J | **Main findings:** Developing an identity is integral to the health care professional. Five cultural dimensions of personal profession-al identity have evolved. First, identity is a negotiated experience that is defined not only by how students define themselves, but also by how others see them. In clinical education, students work collaboratively with preceptors to discover an identity that is nurtured through interactions with others. Next, identity within a community is established by interpretation of familiar and unfamiliar cues encountered in the learning environment. These cues can be verbal and nonverbal and come from a variety of sources including the preceptor, other health care providers, and patients them-selves. How these cues are interpreted often depends on the nature of the interaction and communication between educator and the student. Reflection on practice anchors these cues and helps formulate the professional identity of health care students. |
| 144 | D.L. Klamen, R. Williams and S. Hingle | Getting Real: Aligning the Learning Needs of Clerkship Students With the Current Clinical Environment | **2019** | **Acad Med** | **Abstract:** The authors present follow-up to a prior publication, which proposed a new model for third-year clerkships. The new model was created to address deficiencies in the clinical year and to rectify a recognized mismatch between students' learning needs and the realities of today's clinical settings. The new curricular model was implemented at Southern Illinois University School of Medicine in academic year 2016-2017. Guiding principles were developed. These were to more deeply engage students in experiential learning through clinical immersion; to pair individual faculty with individual students over longer periods of time so real trust could be developed; to provide students with longitudinal clinical reasoning education under controlled instructional conditions; to simplify goals and objectives for the core clerkships and align them with student learning needs; and to provide students with individualized activities to help them explore areas of interest, choose their specialty, and improve areas of clinical weakness before the fourth year. The authors discuss reactions by faculty and students to the new curriculum, which were mostly positive, as well as several outcomes. Students showed very different attitudes toward what they defined as success in the clerkship year, reflective of their deeper immersion. Students spent more time working in clinical settings and performed more procedures. Performance on Step 2 Clinical Knowledge and Clinical Skills was unchanged from traditional clerkship years. The 2015 article called for rethinking the third-year clerkships. The authors have shown that such change is possible, and the new curriculum can be implemented with successful early outcomes. |
| 145 | P. Régo, R. Peterson, L. Callaway, M. Ward, C. O'Brien and K. Donald | Using a structured clinical coaching program to improve clinical skills training and assessment, as well as teachers' and students' satisfaction | **2009** | **Med Teach** | **Introduction:** The ability to deliver the traditional apprenticeship method of teaching clinical skills is becoming increasingly more difficult as a result of greater demands in health care delivery, increasing student numbers and changing medical curricula. Serious consequences globally include: students not covering all elements of clinical skills curricula; insufficient opportunity to practise clinical skills; and increasing reports of graduates' incompetence in some clinical skills.  **Methods:** A systematic Structured Clinical Coaching Program (SCCP) for a large cohort of Year 1 students was developed, providing explicit learning objectives for both students and paid generalist clinical tutors. It incorporated ongoing multi-source formative assessment and was evaluated using a case-study methodology, a control-group design, and comparison of formative assessment scores with summative Objective Structured Clinical Examination (OSCE) scores.  **Results:** Students demonstrated a higher level of competence and confidence, and the formative assessment scores correlated with the Research students' summative OSCE scores. SCCP tutors reported greater satisfaction and confidence through knowing what they were meant to teach. At-risk students were identified early and remediated.  **Discussion:** The SCCP ensures consistent quality in the teaching and assessment of all relevant clinical skills of all students, despite large numbers. It improves student and teacher confidence and satisfaction, ensures clinical skills competence, and could replace costly OSCEs. |
| 146 | A.N. Gheasuddin, R. Misra and J. Patel | Use of an apprenticeship model to facilitate prescribing learning on clinical placements | **2022** | **Med Teach** | **Abstract:** As three final year medical students at UCL, our limited experiences of prescribing practice are in line with that expressed in Linton and Murdoch-Eaton’s (2020) ‘Twelve tips for facilitating medical students prescribing learning on clinical placement.’ It is widely accepted that prescribing is a commonly tested skill in OSCE examinations, but the authentic practice is relatively hard to come by, even more so during this tumultuous year with the move towards remote prescriptions, further hindering our efforts. With an overall error rate of 7.5% in prescriptions written by junior doctors, it is imperative that medical students use their own initiative to seek out practice opportunities wherever possible. Implementation of an apprenticeship model at UCL—in which students are directly assigned to work within a specific healthcare team—during this disrupted year has meant that we as a student body are overall more involved with individual patients’ journeys. This has meant that we feel more confident not only in suggesting medications for patients but also in following up on the prescriptions ourselves. The apprenticeship model also allows immediate feedback and negates the need for a specific medical student pre-prescription as suggested in Tip 5, allowing us to simultaneously learn from our mistakes while ensuring the utmost patient safety, as our prescriptions are always checked by junior doctors. As is the nature of clinical placements, this one-to-one teaching has been variable between hospitals and clinical teams. Subsequently, supplementation by pharmacist-led workshops throughout the year, as suggested in Tip 8, to fill in any gaps in knowledge has been very valuable. This MDT approach to teaching complements the apprenticeship model well, as we are taught from different perspectives, helping us to understand and implement the best possible practices. We feel that the combination of understanding the rationale behind prescriptions, through the apprenticeship model, and the active writing of them on the wards makes the most effective practice. The apprenticeship model was brought in to mitigate the effects of COVID-19 disruption on our learning. However, we feel that it has been beneficial in many ways, particularly in targeting the universal lack of prescribing practice throughout medical school. We hope that other schools can take this on board when developing their curriculums. |
| 147 | K. A. Bettin | The Role of Mentoring in the Professional Identity Formation of Medical Students | **2020** | Orthop Clin North Am. | **Abstract:** Professional identity formation (PIF) of medical students encompasses how students learn to think, do, and act as physicians. A key component of PIF is socialization, which includes mentoring. Mentoring influences students' career specialty choice, while providing a safe and nurturing environment to form their own professional identities. Mentoring of medical students by orthopedic surgeons may increase interest in the specialty. Suggestions for utilizing mentoring for the PIF of medical students and to increase diversity in orthopedics are discussed. |
| 148 | A. Baerheim and J. Thesen | Medical students' evaluation of preceptorship in general practice in Vestlandet | **2003** | Tidsskr Nor Laegeforen | **Abstract:** Preceptorship in general practice is increasingly popular the world over. Norwegian medical curricula include 4-8 weeks of such instruction. In this study we focus on the experience of University of Bergen medical students with preceptorships, based on their written evaluation. In spring 2002, 60 sixth-year medical students had four-week preceptorships. All students returned an evaluation form, giving free-text descriptions on how satisfied they were with structure and supervision. Many students experienced working in general practice as surprisingly exiting and educational. Their preceptors were mostly experienced as very competent, and working conditions reported as safe and including. Quite a large number of students received supervision during clinical work and got constructive feedback from their preceptors, and many participated in structuring their own work schedule and educational progression. Even though the preceptorship period was experienced by most of the students as productive for learning and evoked much enthusiasm, there is still a need for quality assurance in order to secure a sufficient level of skills among all preceptors. |
| 149 | B.P. Golden, B.L. Henschen, L.A. Gard, E.R. Ryan, D.B. Evans, J. Bierman and K.A. Cameron | Learning to be a doctor: Medical students' perception of their roles in longitudinal outpatient clerkships | **2018** | Patient Educ Couns | **Objective:** To understand how medical students perceive their roles in early longitudinal primary care clerkships.  **Methods:** Medical students enrolled in one of two longitudinal primary care clerkships - Education-Centered Medical Home (ECMH) or Individual Preceptorship (IP) - participated in semi-structured interviews. Interviews were recorded, transcribed, and analyzed using a grounded theory and constant comparative approach.  **Results:** Students (N = 35) in both clerkships perceived benefits of early clinical exposure, reflecting positively on having time to interact with patients. Identified roles ranged from shadower to collaborator to diagnostician; a progression from position-centered to more patient-centered roles emerged. ECMH students also identified as health educators, care managers, and mentors. IP students described the clerkship primarily as an opportunity to acquire clinical knowledge and practice skills, expressing perceptions of being a transient "visitor" in the clinic, whereas ECMH students reported taking an active role in continuity care of patients.  **Conclusion:** Students identified benefits of early longitudinal outpatient primary care clerkships, supporting the inclusion of these experiences within medical school curricula. Clerkships with an emphasis on longitudinal and team-based care may further promote student participation in patient care and professional development. |
| 150 | G.D. Harris | Professionalism: Part II - Teaching and assessing the learner's professionalism | **2004** | Fam Med | This articles discusses how the preceptor can teach concepts of professionalism during office-based care and assess the learner’s professionalism. Teaching professionalism begins with the first-year medical student  in the classroom and continues as the student progresses into the clinical arena. A curriculum that includes clinical and professional ethics, social issues in medicine, community service activities, and longitudinal patient care is necessary. It is a responsibility of the institution to create an environment for professionalism, have leaders who are involved with mentorship, and ensure that the entire faculty is contributing to the same outcome. For most medical students, their initial exposure and preparation for the “outside” world of medical practice occurs during an office based preceptorship. This is a unique opportunity for the preceptor to emphasize to learners their responsibility to patients, to society, to their profession, and to themselves. |
| 151 | L. Sheu, S. Goglin, S. Collins, P. Cornett, S. Clemons and P.S. O’Sullivan | How Do Clinical Electives during the Clerkship Year Influence Career Exploration? A Qualitative Study | **2021** | each Learn Med | **Problem:** Although many students begin medical school with some idea of their specialty interest, up to 80% of these students choose a different specialty by their final year. This pivot tends to happen in the clerkship year, when students are immersed in the clinical environment, gaining a practical understanding of the day-to-day work in different fields. Yet, in this year students have limited experiences with specialties. Clinical electives during the clerkship year may aid students in career development. The authors examined student career exploration through the lens of social cognitive career theory (SCCT). SCCT posits three variables that influence career development: personal goals, self-efficacy, and understanding outcome expectations. With this framework, the authors sought to understand how a program of clinical electives during the clerkship year influences students' perceptions of their career exploration. We aimed to: (1) describe an innovative clerkship elective program designed for career exploration, and (2) explore how this influenced students' career exploration using qualitative analysis.  **Intervention:** Beginning in 2018, students at our institution were required to participate in three 2-week clinical electives during their clerkship year, called Clinical Immersive Experiences (CIExes). CIExes were categorized into four different types: apprenticeship, clinical skills building, integrative (multi-disciplinary), or subspecialty. Authors invited fourth year students to participate in interviews (January to March 2019) about how they selected electives and how these electives contributed to their career exploration. Interviews continued until reaching information sufficiency. Authors coded and analysed transcripts using template analysis.  **Context:** This curricular intervention took place in the context of large-scale curricular redesign. Students began clerkships partway into their second year of medical school. The family and community medicine clerkship, which was previously a 6-week core clerkship, was changed to a longitudinal format, thus freeing up 6 weeks for electives. Other core clerkships included anaesthesia (2 weeks), medicine (8 weeks), neurology (4 weeks), obstetrics and gynaecology (6 weeks), paediatrics (6 weeks), psychiatry (4 weeks), and surgery (8 weeks).  **Impact:** From 15 student interviews, we identified three major themes. First, CIExes facilitated personalized career exploration. All students felt that at least one elective helped them solidify their decision about a specialty choice. Second, CIExes promoted focused learning and skills development that complemented core rotations. They noted the benefit of positive relationships with supervisors, particularly attendings, during these electives. Third, students highlighted how these electives fostered a positive learning environment and enhanced wellbeing. SCCT clarified how the CIEx program helped students advance their personal goals, self-efficacy, and outcome expectations during a pivotal time in medical school.  **Lessons Learned:** We learned that from the student perspective, the inclusion of clinical electives in the clerkship year benefited students' career exploration by helping them develop and refine their career goals, increase self-efficacy, and test outcome expectations in a meaningful way as anticipated from SCCT. In addition, we found that CIExes created a positive learning environment that allowed deep relationships to develop in fields of interest and that supported a strong sense of wellbeing. |
| 152 | A. Hay, S. Smithson, K. Mann and T. Dornan | Medical students’ reactions to an experience-based learning model of clinical education | **2013** | Perspect Med Educ | **Abstract:** An experience-based learning (ExBL) model proposes: Medical students learn in workplaces by ‘supported participation’; affects are an important dimension of support; many learning outcomes are affective; supported participation influences students’ professional identity development. The purpose of the study was to check how the model, which is the product of a series of earlier research studies, aligned with students’ experiences, akin to the ‘member checking’ stage of a qualitative research project.  In three group discussions, a researcher explained ExBL to 19 junior clinical students, who discussed how it corresponded with their experiences of clinical learning and were given a written précis of it to take away. One to 3 weeks later, they wrote 500-word reflective pieces relating to their subsequent experiences with ExBL.  Four researchers conducted a qualitative analysis. Having found many instances of responses ‘resonating’ to the model, the authors systematically identified and coded respondents’ ‘resonances’ to define how they aligned with their experiences. 120 resonances were identified. Seventy (58 %) were positive experiences and 50 (42 %) negative ones. Salient experiences were triggered by the learning environment in 115 instances (96 %) and by learners themselves in 5 instances (4 %), consistent with a strong effect of environment on learning processes. Affective support was apparent in 129 of 203 statements (64 %) of resonances and 118 learning outcomes (58 %) were also affective. ExBL aligns with medical students’ experiences of clinical learning. Subject to further research, these findings suggest ExBL could be used to support the preparation of faculty and students for workplace learning. |
| 153 | L. Abbey, R. Willett, R. Selby-Penczak and R. McKnight | Social Learning: Medical Student Perceptions of Geriatric House Calls | **2010** | Gerontol Geriatr Educ | **Abstract:** Bandura's social learning theory provides a useful conceptual framework to understand medical students' perceptions of a house calls experience at Virginia Commonwealth University School of Medicine. Social learning and role modeling reflect Liaison Committee on Medical Education guidelines for "Medical schools (to) ensure that the learning environment for medical students promotes the development of explicit and appropriate professional attributes (attitudes, behaviors, and identity) in their medical students." This qualitative study reports findings from open-ended survey questions from 123 medical students who observed a preceptor during house calls to elderly homebound patients. Their comments included reflections on the medical treatment as well as interactions with family and professional care providers. Student insights about the social learning process they experienced during house calls to geriatric patients characterized physician role models as dedicated, compassionate, and communicative. They also described patient care in the home environment as comprehensive, personalized, more relaxed, and comfortable. Student perceptions reflect an appreciation of the richness and complexity of details learned from home visits and social interaction with patients, families, and caregivers. |
| 154 | L. Côté and P.A. Laughrea | Preceptors' understanding and use of role modeling to develop the CanMEDS competencies in residents | **2014** | **Acad Med** | **Purpose:** Role modeling by preceptors is a key strategy for training residents in the competencies defined within the CanMEDS conceptual framework. However, little is known about the extent to which preceptors are aware of the importance of role modeling or how they perceive and enact it in their daily interactions with residents. The purpose of this study was to describe how preceptors understand and use role modeling to develop CanMEDS competencies in residents.  **Method:** In 2010, the authors conducted a descriptive qualitative study with preceptors in medical, surgical, and laboratory specialties who supervised residents on a regular basis at the Université Laval Faculty of Medicine (Québec, Canada). Respondents participated in semi structured, individual interviews. An inductive thematic analysis of interview transcripts was conducted using triangulation.  **Results:** Most participants highlighted the importance of role modeling to support residents' development of the CanMEDS competencies, particularly communication, collaboration, and professionalism, which preceptors perceived as "less scientific" and the most difficult to teach. Although most participants reported using an implicit, unstructured role modeling process, some described more explicit strategies. Eight types of educational challenges in role modeling the CanMEDS competencies were identified, including encouraging reflective practice, understanding the competencies and their importance in one's specialty, and being aware of one's strengths and weaknesses as a clinical teacher.  **Conclusions:** Preceptors are aware of the importance of role modeling competencies for residents, but many do so only implicitly. This study's findings are important for improving strategies for role modeling and for the professional development of preceptors. |
| 155 | E.A. Goldstein, C. F. MacLaren, S. Smith, T.J. Mengert, R.R. Maestas, H.M. Foy, et al. | Promoting fundamental clinical skills: A competency-based college approach at the University of Washington | **2005** | **Acad Med** | **Abstract:** The focus on fundamental clinical skills in undergraduate medical education has declined over the last several decades. Dramatic growth in the number of faculty involved in teaching and increasing clinical and research commitments have contributed to depersonalization and declining individual attention to students. In contrast to the close teaching and mentoring relationship between faculty and students 50 years ago, today's medical students may interact with hundreds of faculty members without the benefit of a focused program of teaching and evaluating clinical skills to form the core of their four-year curriculum. Bedside teaching has also declined, which may negatively affect clinical skills development. In response to these and other concerns, the University of Washington School of Medicine has created an integrated developmental curriculum that emphasizes bedside teaching and role modeling, focuses on enhancing fundamental clinical skills and professionalism, and implements these goals via a new administrative structure, the College system, which consists of a core of clinical teachers who spend substantial time teaching and mentoring medical students. Each medical student is assigned a faculty mentor within a College for the duration of his or her medical school career. Mentors continuously teach and reflect with students on clinical skills development and professionalism and, during the second year, work intensively with them at the bedside. They also provide an ongoing personal faculty contact. Competency domains and benchmarks define skill areas in which deepening, progressive attention is focused throughout medical school. This educational model places primary focus on the student. |
| 156 | K. Jones and S. Reis | Learning through vulnerability: A mentor-mentee experience | **2010** | Ann Fam Med | **Abstract:** The following essay, drawn from the journals and work notebook of a family medicine resident and a visiting clinical mentor, chronicles their work together in an Advanced Clinical Mentoring program. This program included afternoons of direct clinical observation immediately followed by feedback sessions. In addition to addressing specific professional issues, such as time management, limiting patient encounters, agenda matching, and the One-Minute Preceptor model, the authors developed personally as they opened themselves to learning and growing as a clinician and a teacher. |
| 157 | S. Kalén, T. Stenfors-Hayes, U. Hylin, M.F. Larm, H. Hindbeck and S. Ponzer | Mentoring medical students during clinical courses: a way to enhance professional development | **2010** | **Med Teach** | **Background:** Mentoring is known to develop professional attributes and facilitate socialization into a profession. Only a few structured mentoring programmes for medical students have been reported in the literature.  **Aim:** The objective of this study was to investigate undergraduate medical students' experiences and perceptions of one-to-one mentoring and whether they felt that the mentorship promoted their personal and professional development.  **Methods:** Medical students (n = 118) during their third and fourth years of their studies were offered a personal mentor for 2 years and followed up via a questionnaire when the mentoring programme was completed. Statistical software was used to compute data. Open-ended questions were analyzed by content analysis.  **Results:** Most of the respondents experienced that the mentoring programme had facilitated their professional and personal development. The role of the mentor was experienced as being more supportive than supplying knowledge. The students appreciated talking to a faculty not connected with their courses. The few barriers to a successful mentorship were mainly related to timing logistics and 'personal chemistry'.  **Conclusions:** One-to-one mentoring during clinical courses seems to enhance the medical student's professional and personal development. Future studies are needed to get a deeper understanding and knowledge about factors of importance for successful mentorship. |
| 158 | M. Tariq, S. Iqbal, S.I. Haider and A. Abbas | Using the cognitive apprenticeship model to identify learning strategies that learners view as effective in ward rounds | **2021** | Postgrad Med J | **Background:** Cognitive apprenticeship model (CAM) is an instructional model for situated learning. There is limited data available on application of the CAM in clinical settings. The aim of the study was to identify learning strategies using CAM, which in the opinion of learners are effective in ward rounds.  **Methods:** Participants were residents and medical students who rotated through internal medicine at Aga Khan University Hospital, Karachi. We sought learners' opinion on a structured questionnaire based on four principal dimensions of cognitive apprenticeship. A previously determined set of 10 defined competencies were compared with CAM's six teaching/learning (T/L) methods (modelling, coaching, scaffolding, articulation, reflection and exploration) as well. Mean and SD were calculated. Mann-Whitney test was used to compare scores.  **Results:** Of 195 participants, there were 100 men (51.3%) and 95 women (48.7%). Perceived learning for six T/L methods, ranged from 3.7 to 3.9 (max=5). Coaching and scaffolding had the highest scores. Statistically significant difference between the student and resident groups was noted. Medical students perceived coaching and scaffolding (4.1 and 4.05) and residents rated coaching, articulation and exploration as most effective (3.9 and 3.8). Majority (82.1%) reported a positive learning environment in wards.  **Conclusions:** CAM enabled to identify two T/L methods (scaffolding and coaching) that are important for learning in ward round. Limited differences in perceived effectiveness of the T/L methods indicate that variety can be used to sustain interest in learners. Positive learning environment, team diversity and tasks of increasing complexity contribute to learning. |
| 159 | C. Braniff, R.A. Spence, M. Stevenson, M. Boohan and P. Watson | Assistantship improves medical students' perception of their preparedness for starting work | **2016** | **Med Teach** | **Background:** The GMC has recommended introducing student assistantships during which final year students, under supervision, undertake most of the responsibilities of a FY1 doctor. The Medical School at Queen's University Belfast in 2011/12 introduced an assistantship programme. We have evaluated the impact of the assistantship on students' perception of their preparedness for starting work.  **Methods:** Students were asked to complete a questionnaire at the beginning of the assistantship. It assessed the students' perception of their preparedness in five areas: clinical and practical skills, communications skills, teaching and learning, understanding the work environment and team working. After the assistantship they again completed the questionnaire. Comparison of the results allowed an assessment of the impact of the assistantship.  **Results:** There was a statistically significant improvement in the students' perception of their preparation for 49 of 56 tasks contained within the questionnaire. After the assistantship 81.2% of students felt well prepared for starting work compared with 38.9% before the assistantship. 93.9% agreed that the assistantship had improved their preparedness for starting work.  **Conclusions:** The assistantship at Queen's University improves medical students' perception of their preparedness for starting work. The majority of medical students feel well prepared for starting work after completing the assistantship. |
| 160 | K. Iwata and D. Gill | Learning through work: clinical shadowing of junior doctors by first year medical students | **2013** | **Med Teach** | **Abstract:** Early clinical contact (ECC) is a key feature of undergraduate programmes, yet they make significant demands on senior clinicians delivering it and usually focus on patient contact. Aims: To explore the potential of an ECC activity oriented to work as a junior doctor and the clinical environment, and the use of very junior doctors as facilitators of this learning. Methods: For two academic years, all first year medical students at UCL Medical School shadowed a Foundation Year (FY) doctor for a four-hour shift to experience and understand the work of junior doctors. Feedback from students and FY doctors was gathered and analysed. Results: The students found the FY doctors to be good near-peer tutors and enjoyed exploring the clinical environment, but felt that the unstructured learning environment was difficult. The FY doctors felt that learning in and about the clinical environment was an important learning outcome for the students, although they found supervising junior medical students in a shadowing context difficult. Conclusions: FY doctors are an effective and under-utilised resource in introducing novices to the role of a medical professional in the clinical environment; however students and FY doctors need support to maximise the learning potential of early shadowing. |
| 161 | D.S. Brody, K. Ryan and M.A. Kuzma | Promoting the development of doctoring competencies in clinical settings | **2004** | **Fam Med** | **Background and objectives:** This UME-21 project was developed to promote a variety of clinical competencies during a 12-week medicine clerkship for third-year students.  **Methods:** The clerkship is divided into three 4-week rotations--two inpatient rotations and one outpatient rotation. During each rotation, students select a competency, review the module about that competency on the clerkship Web site, and perform a literature search. Learning exercises prompt students to ask their preceptor to model and discuss the performance of the competency on at least one patient and to provide feedback on their performance at least twice. At the end of each rotation, students are required to write about what they learned from the articles they read, write a critical analysis of their performance of the competency on one patient, and complete an evaluation questionnaire. This report is based on the results from the students' evaluation questionnaire.  **Results**: At the end of the first six rotations, 120 students completed 330 evaluations of the course (93% response rate). The most frequently selected competency modules were behavior modification and patient education. In 81.5% of the evaluations, students felt that there was at least moderate improvement in their ability to perform the selected competency during the rotation. By the end of the rotation, in 85.3% of the evaluations, students indicated that they were confident performing the competency most or almost all of the time. Observing the preceptor was the component of the curriculum most often rated as helpful (59.1%), followed by literature review (57.9%), reviewing the Web site module (45.2%), and observation and feedback by the preceptor (32.7%).  **Conclusions:** Based on student reports, the approach described in this paper appears to be a promising way to teach important doctoring competencies in a clinical setting. |
| 162 | D.M. Haas, B. Hadaie, M. Ramirez, A.L. Shanks and N.P. Scott | Resident research mentoring teams: A support program to increase resident research productivity | **2023** | J Grad Med Educ | **Background:**Scholarly activity is a requirement for most graduate medical education training programs. However, barriers exist for residents to accomplish projects.  **Objective:**To evaluate the correlation between a resident research mentoring team (RRMT) program and meeting presentations and publications of resident research projects. We further plan to report feasibility of the RRMT.  **Methods:**We performed a before-and-after study of meeting presentations and/or publication of resident research projects before institution of the RRMT (2004-2011) and post-RRMT implementation (2016-2019). The RRMT is a diverse group of faculty, statisticians, and research staff who meet regularly with residents to provide guidance for their research studies. It is part of overall research support from the department, which also includes biostatistics, database and regulatory help, travel funds, and project budget funds. Data on meeting presentations and publications were collected from Google Scholar, PubMed, Scopus, and the IUPUI ScholarWorks institutional repository, using resident and faculty names and titles of projects. Comparisons of pre- and post-RRMT groups were made.  **Results:**Seventy-four residents were in the pre-RRMT group and 40 were in the post-RRMT group. Post-RRMT residents published, presented, and combined published or presented their projects more frequently than those in pre-RRMT group (57.5% vs 28.4%, *P*=.002; 50% vs 16.2%, *P*=.001; 67.5% vs 37.8%, *P*=.002). Controlling for winning a Research Day award and pursuing a fellowship, being in the post-RRMT group was independently associated with presentation or publication of the resident research project (OR 3.62, 95% CI 1.57-8.83).  **Conclusions:**Support of resident scholarly activity, such as thorough implementation of a program like the RRMT, is associated with increased presentations and publications of research projects. |
| 163 | J.S.K. Chan, D.H.H. Lau, E. King, Y.K.L. Shum, L. Roever, T. Liu, et al. | Virtual medical research mentoring | **2023** | Clin Teach | **Background:**Medical research is important for professional advancement, and mentoring is a key means by which students and early-career doctors can engage in research. Contrasting international research collaborations, research mentoring programmes are often geographically limited. As the COVID-19 pandemic has led to increased use of online technology for classes and conferences, a virtual, international approach to medical research mentoring may be valuable.  **Approach:**We hereby describe our experience at the Cardiovascular Analytics Group, a virtual international medical research mentoring group established in 2015. We make use of virtual platforms in multi-level mentoring with peer mentoring and emphasise active participation, early leadership, an open culture, accessible research support and a distributed research workflow.  **Evaluation:**With 63 active members from 14 different countries, the Group has been successful in training medical students and early-career medical graduates in academic medicine. Our members have led over 100 peer-reviewed publications of original research and reviews since 2015, winning 13 research prizes during this time.  **Implications:**Our accessible-distributed model of virtual international medical research collaboration and multi-level mentoring is viable and efficient and caters to the needs of contemporary healthcare. Others should consider building similar models to improve medical research mentoring globally. |
| 164 | M.B. Joe, A. Cusano, J. Leckie, N. Czuczman, K. Exner, H. Yong, et al. | Mentorship programs in residency: A scoping review | 2023 | J Grad Med Educ | **Background:** Mentorship during residency training is correlated with improved outcomes. Many residency programs have implemented formal mentorship programs; however, reported data for these programs have not been previously synthesized. Thus, existing programs may fall short on delivering effective mentorship.**Objective:** To synthesize current literature on formal mentorship programs in residency training in Canada and the United States, including program structure, outcomes, and evaluation.**Methods:** In December 2019, the authors performed a scoping review of the literature in Ovid MEDLINE and Embase. The search strategy included keywords relevant to mentorship and residency training. Eligibility criteria included any study describing a formal mentorship program for resident physicians within Canada or the United States. Data from each study were extracted in parallel by 2 team members and reconciled.**Results:** A total of 6567 articles were identified through the database search, and 55 studies met inclusion criteria and underwent data extraction and analysis. Though reported program characteristics were heterogenous, programs most commonly assigned a staff physician mentor to a resident mentee with meetings occurring every 3 to 6 months. The most common evaluation strategy was a satisfaction survey at a single time point. Few studies performed qualitative evaluations or used evaluation tools appropriate to the stated objectives. Analysis of data from qualitative studies allowed us to identify key barriers and facilitators for successful mentorship programs. **Conclusions:** While most programs did not utilize rigorous evaluation strategies, data from qualitative studies provided insights into barriers and facilitators of successful mentorship programs, which can inform program design and improvement. |
| 165 | A. Lupi, L. Shu and A. Lopez | Local mentoring as a strategy to recruit a more diverse physician workforce | 2022 | Acad Med | **Abstract:** With a lack of diversity in medicine that has remained unchanged over decades, there is an important role for targeted mentorship in underrepresented communities. One proposal has been to start pipeline programs between medical schools and undergraduate institutions with diverse student populations, especially Historically Black Colleges and Universities. Our institution (Vanderbilt University School of Medicine) has implemented such a program; however, we recognize that by design, pipeline programs offer many resources to a small, select group of students. We saw an opportunity to create and share free resources targeted toward students who are underrepresented in medicine to broaden our institution’s scope of mentorship.  A group of students at our university developed a written guide on the premedical process, a template schedule and advice for self-studying for the Medical College Admission Test, and a spreadsheet to track costs associated with applying to medical school. We have reached many students at local universities and the Historically Black Colleges and Universities in our pipeline program to offer these resources. Furthermore, we have started a virtual webinar series covering important aspects of the premedical process, offering live feedback and advice to prospective medical students.  Future goals include developing a robust collection of shadowing and research opportunities for students within our mentorship system. The COVID-19 pandemic has made it even more challenging for premedical students to access these in-person resources and opportunities without institutional assistance. Ultimately, our hope is to lower the barriers of the medical school application process to increase recruitment of a diverse physician workforce. We encourage groups at other medical schools to employ similar initiatives of local outreach to achieve this goal. |
| 166 | S. Drossard and A. Härtl | Development and implementation of digital peer mentoring in small groups for first-year medical students. | **2024** | **GMS J Med Educ** | **Introduction:**Mentoring has become an important educational strategy in medical training. Peer mentoring (PM) can enhance student experience and support transition to higher education. This article documents the implementation of an online peer mentoring program for first year medical students at a newly founded medical school in Germany during the COVID-19 pandemic and its development into in-person PM.  **Project description:**We developed the program in close collaboration between students and teachers. Students were invited to apply as peer mentors via email; they received instructions and reflected on their role and experiences in meetings before, during and after the semester. One or more peer mentors were assigned randomly to a student group. We evaluated the program with an online survey inspired by the "Modified Mentorship Effectiveness Scale". After successful piloting PM was implemented into the core curriculum.  **Results:**In 2020 we assigned 17 peer mentors to 14 groups of 6-7 students. Groups met 3 or more times via Zoom. Overall satisfaction was high. Both student groups reported benefits for their personal and professional identity formation. Atmosphere in online meetings was excellent. Most important topics were exams/learning strategies. In 2021 meetings were held in person. Overall satisfaction, perceived benefits and learning atmosphere were again rated very positively. Most students preferred many-to-many PM and random matching.  **Conclusion:**The implementation of PM was successful and beneficial for the participating students. PM can help first-year medical students reduce anxiety, improve self-organization and orientation at university. It fosters identity formation and has positive effects on peer mentees as well as on peer mentors. |
| 167 | A. Scholz, V. Gehres, A. Schrimpf, M. Bleckwenn, T. Deutsch and A.K. Geier | Long-term mentoring relationships in undergraduate longitudinal general practice tracks - a qualitative study on the perspective of students and general practitioners. | **2023** | **Med Educ Online** | **Background:**Longitudinal general practice tracks have been established in medical faculties in Europe and worldwide to attract more graduates to general practice careers. In many programs, long-term mentoring relationships play an important role in providing students with positive role models, regular practical experiences, and acquisition of clinical skills in a community context. However, little is known about students' and general practitioner mentors' expectations, experiences, challenges, and ideas for improvement within these long-term mentoring relationships in general practice in our medical education system.  **Methods:**Qualitative study based on semi-structured interviews with 15 students and 13 mentors. Interviews were audio-recorded and transcribed verbatim. MAXQDA was used for data analysis, following a mixed deductive/inductive approach.  **Results:**Both groups had few and rather unstated expectations, particularly regarding their relationships. Consequently, expectations were often not clearly communicated. Nevertheless, a high level of satisfaction and good opportunities for teaching were achieved for both sides. The evolving familiarity facilitated a positive learning environment. Students valued independent medical tasks continuously adjusted to their current abilities. However, some felt a reluctance to demand their mentor's time and consideration. Conversely, the mentors criticized a lack of initiative from some of the students and wished that they would get more actively involved. Students, in contrast, wished for more guidance at the start of the project and joint events to deepen the relationship.  **Conclusions:**With this study, we gained detailed insights into and understanding of the nature of long-term relationships between students and mentors. Points for improvement revealed included: 1) education of both participating groups on the goals and benefits of mentoring, including binding expectations for the participants; 2) intensified support and training of teaching physicians; 3) structured and accompanied establishment of initial contact between mentor and mentee; and 4) encouraged additional shared (teaching) time, individualized timing, and intensification, if desired. |
| 168 | M.R. Shen, E. Tzioumis, E. Andersen, K. Wouk, R. McCall, W. Li, et al. | Impact of mentoring on academic career success for women in medicine: A systematic review | **2022** | **Acad Med** | **Purpose:**Research has shown that barriers to career success in academic medicine disproportionately affect women. These barriers include inadequate mentoring, which may perpetuate the underrepresentation of women in senior leadership positions. The purpose of this review was to summarize the qualitative and quantitative evidence of the impact of mentoring on women's career outcomes and to inform future interventions to support the promotion and retention of women in academic medicine.  **Method:**The authors conducted a systematic review of original research published in English-language, peer-reviewed journals through March 20, 2020. Search terms related to mentorship, women, and academic medicine. The authors searched MEDLINE, Embase, Scopus, Current Contents Connect via Web of Science, Cochrane Library, and PsycINFO. They excluded studies not specifically addressing women and those without gender-stratified outcomes. They extracted and analyzed the following data: study design, population, sample size, response rate, participant age, percentage of women, mentoring prevalence, and outcomes.  **Results:**Of 2,439 citations identified, 91 studies met the inclusion criteria, including 65 quantitative and 26 qualitative studies. Mentoring was associated with objective and subjective measures of career success. Women perceived mentorship to be more valuable to their career development yet were more likely to report having no mentor. Additionally, women were more likely to report lower levels of research productivity, less career satisfaction, and greater barriers to promotion. Qualitative results indicated that women had less access to informal mentoring and family responsibilities had a greater effect on their career outcomes. Professional networking, female mentors, and relational aspects of mentoring were common themes.  **Conclusions:**This review examined gender disparities in mentoring and the impact on research productivity, promotion success, and career satisfaction for women in academic medicine. Institution-supported mentoring programs are needed to facilitate identification of appropriate mentors and promotion of a more equitable academic career environment for women. |
| 169 | C.S. Hartlage and D.M. Sosa | Everyone deserves a seat at the table: Mentoring to uplift minoritized trainees | 2023 | Acad Med | Navigating academic medicine as students who are transgender is not easy. It is difficult to understand when someone has not lived it themselves, and mentors in academic medicine who are transgender are few and far between. This can be incredibly frustrating, isolating, and overwhelming. However, some mentors possess the unique ability to uplift the voices of those from marginalized communities, even without living the same experiences of those people. When trainees are learning in a system that was not built for them, mentorship can be the difference between success and burnout. |
| 170 | S. I. Lee and D. A. Bluemke | Mentoring in academic radiology. | 2022 | Radiology | **Abstract:** Academic radiologists fulfill a tripartite mission—patient care, research, and teaching. In carrying out these activities, academic radiologists also have a wonderful opportunity and, we think, a responsibility, to mentor trainees and junior faculty. These newer members of our profession are a vital component of the department’s workforce. Furthermore, most already understand the power of mentoring as a contribution to professional success and satisfaction. For those who choose to mentor the next generation, the rewards are long lasting and satisfying. Mentorship can keep more senior academicians up to date and help avoid burnout. |
| 171 | M. Murphy, H. Record, J. K. Callander, D. Dohan and J. R. Grandis | Mentoring relationships and gender inequities in academic medicine: Findings from a multi-institutional qualitative study. | **2022** | **Acad Med** | **Purpose:**This study examined how mentoring relationships may reinforce or mitigate gender inequities in academic medicine.  **Method:**In-depth, semi structured interviews with medical school faculty members (52 women and 52 men) were conducted at 16 institutions across the United States in 2019. Institutions were recruited using a purposive sampling strategy to seek diversity in geography, ownership (private or public), and prestige. Within institutions, purposive sampling was used to recruit equal numbers of women and men and to seek diversity in degree type (MD, PhD), age, and career stage. A coding scheme was developed through iterative analysis of the interview transcripts. All interview transcripts were then coded with the goal of identifying intersections between mentorship and experiences of and responses to gender inequities.  **Results:**Four key themes at the intersection of mentoring relationships and gender inequities were identified. (1) Both women and men became aware of gender inequities in academic medicine through relationships with women mentors and mentees. (2) Both women and men mentors recognized the challenges their female mentees faced and made deliberate efforts to help them navigate an inequitable environment. (3) Both women and men mentors modeled work-family balance and created family friendly environments for their mentees. (4) Some women, but no men, reported being sexually harassed by mentors.  **Conclusions:**This study shows that mentoring relationships may be a context in which gender inequities are acknowledged and mitigated. It also shows that mentoring relationships may be a context in which gender inequities, such as sexual harassment, may occur. Sexual harassment in academic medicine has been widely documented, and gender inequity in academic medicine has proved persistent. While mentoring relationships may have the potential to identify and mitigate gender inequities, this study suggests that this potential remains largely unrealized. |
| 172 | M. Jafari and A. Moodi Ghalibaf | Peer-research learning and mentoring for undergraduate medical students: Benefits and challenges. | 2022 | Res Dev Med Educ | **Abstract:** Today, research efficiency is a critical element not only for scientific institutions but also for governments. Previous studies indicate that outstanding high-impact research requires the collaboration of professional researchers and teams of experts. An essential step toward establishing expert researchers and professional research teams involves the collaboration of a professional instructor to train and guide the learners during their research process. On the other hand, peer learning, peer education, and peer mentoring should not be neglected in this field of education, teaching, and learning, as well as training. |
| 173 | L. Stadtlander, A. Ozcan, L. Johnson, B. Nicholson and N. Hyder | Faculty and student online mentoring preferences. | 2022 | Educ Res Pract | **Abstract:** Isolation of online doctoral students intensifies when they transition from coursework to the dissertation/capstone phase, limiting them to interacting with their mentors. A three-round modified Delphi study was conducted to examine mentoring preferences of online doctoral students and faculty. The first round provided qualitative data regarding the preferred mentoring practices for faculty and alumni. Round 1 qualitative data were organized into Likert questions and used in the second round, which resulted in data about frequency of mentoring practices for the same participants from Round 1. The third round provided data about importance of each preference rated by faculty and current students. Findings indicated a dichotomy was present in that mentees’ academic self-esteem appeared to be tied to faculty being responsive. Faculty emphasized mentee autonomy while mentees wanted frequent contact with faculty. |
| 174. | J. Trejo, D. Wingard, V. Hazen, A. Bortnick, K. Van Hoesen, A. Byars-Winston, et al. | A system-wide health sciences faculty mentor training program is associated with improved effective mentoring and institutional climate. | **2022** | J Clin Transl Sci | **Introduction:**Mentorship is critical for faculty success, satisfaction, and engagement. However, many faculty, particularly underrepresented racial/ethnic (UR) faculty, lack access to high-quality mentoring. In an effort to improve mentoring for all faculty, we developed and implemented a formally structured faculty mentor training program (FMTP) across UC San Diego Health Sciences, which included institutional support, mentorship training, and department/division mentorship programs.  **Methods:**FMTP impact was evaluated using three primary outcome variables: mentoring quality, mentoring behaviors, and institutional climate. Participants' self-assessed mentoring competencies were measured using validated instruments.  **Results:**A total of 391 (23%) of Health Sciences faculty participated in FMTP. Participation rate was higher for women than men (30% versus 17%) and highest for UR faculty (39%). FMTP was implemented in 16 of 19 departments. Self-reported mentoring improved for FMTP participants with mentoring quality (*p* = 0.009) and meeting mentees' expectations (*p* = 0.01) continuing to improve for up to 2 years after training. However, participants were unsure if they were meeting UR mentees' expectations. FMTP participants were significantly more satisfied with mentoring quality (*p* < 0.001) compared to non-participants, with the greatest increase in satisfaction reported by UR faculty (38-61%). UR faculty reported improved overall morale (51-61%) and a perception that the environment was supportive for UR faculty (48-70%).  **Conclusion:**The implementation of a system-wide formal structured FMTP was associated with improved faculty satisfaction, quality of mentoring, and institutional climate, especially for UR faculty. |
| 175 | K. Farrukh and T. Hoor | Online mentoring session during COVID-19: Experiences of mentees and mentors - a phenomenology. | 2022 | Professional Med J | **Objective:** To explore perceptions and experiences of undergraduate medical mentees and mentors on online mentoring sessions during COVID-19.  **Study Design:** Qualitative study.  **Setting:** A Private Medical College in Karachi, Pakistan.  **Period:** January 2021-May 2021.  **Material & Methods:** A qualitative study was designed using phenomenological approach and constructivist relativist paradigms. Maximum variation purposeful sampling technique was chosen to involve variety of participants from given population. Unstructured individual interviews and four focus group discussions were done. Content analysis was done for analysis of collected data.  **Results:** Most mentors supported online mentoring. However, the mentees opinion was different from mentors as most of them were not in favor of online mentoring sessions. Some mentees praised blended education since it removed some of the traditional teaching barriers. Mentors have common experience that face-to-face mentoring allows them to inspire mentees and have expressive and meaningful connections with them.  **Conclusion:** Face-to face mentoring provides better opportunities to connect during mentoring sessions however blended, online and on campus mentoring sessions can be provided in emergency situations as COVID-19 crisis. |
| 176 | D.T. Bui, T. Barnett, H. Hoang and W. Chinthammit | Development of a framework to support situational tele-mentorship of rural and remote practice. | 2023 | Med Teach | **Abstract:** Situational tele-mentorship refers to the use of technology to provide interactive, two-way communication between an advisor (the mentor) and a novice (mentee) to enhance the management of a dynamic clinical scenario in real-time. This article develops a conceptual framework to support situational tele-mentorship of healthcare professionals working in rural and remote practices by critically exploring the concept of mentorship within medical education literature and applied to healthcare professionals working in more isolated settings. The situational tele-mentorship framework consists of synchronous telecommunication technologies and the problem-solving process. The end-users of the framework are the mentor located centrally and the mentee dealing with a challenging situation at a remote location using communication technology. The problem-solving process' stages are preparation, identification, action, and evaluation. The mentor and mentee use the 5W1H model, which is a summary of the questions of who, what, where, when, why, and how, applied in two-way communication. This framework provides medical teachers and clinicians with a detailed, yet concise exposition of critical elements required to implement situational tele-mentorship. Healthcare providers can also use this framework to help coordinate resources and manage stakeholders in tele-mentoring situations. |
| 177 | S. Ramani, N. Chugh, M.S. Chisolm, R. Hays, J. McKimm, R. Kusurkar, et al. | Mentoring relationships: A mentee's journey | 2023 | Acad Med | **Abstract:** Mentoring relationships are ideally driven by mentees and help mentees to tackle professional challenges and/or plan career development. Mentees can choose from several mentoring formats: senior, near peer, within or outside the institution, dyadic or network, in person or virtual. Regardless of the format, mentors guide key stages of mentee professional development, balance challenging and supporting the mentee, and help the mentee to reflect and make informed decisions. The figure below illustrates how a mentee starts the journey by identifying potential mentors, meets the mentor to discuss aspirations and challenges, and formulates next steps guided by the mentor. The mentee is the architect, and the mentor is the facilitator and guide. A short- or long-term mentoring relationship can be forged based on goals and compatibility. As depicted by the ladders in the figure below, mentoring relationships may require backward jumps and restarts to deal with changing circumstances. |
| 178 | J. S. Williams, R.J. Walker, K.M. Burgess, L.A. Shay, S. Schmidt, J. Tsevat, et al. | Mentoring strategies to support diversity in research-focused junior faculty: A scoping review. | 2023 | J Clin Transl Sci | **Objective:** The purpose of this scoping review is two-fold: to assess the literature that quantitatively measures outcomes of mentorship programs designed to support research-focused junior faculty and to identify mentoring strategies that promote diversity within academic medicine mentoring programs.**Methods**: Studies were identified by searching Medline using MESH terms for mentoring and academic medicine. Eligibility criteria included studies focused on junior faculty in research-focused positions, receiving mentorship, in an academic medical center in the USA, with outcomes collected to measure career success (career trajectory, career satisfaction, quality of life, research productivity, leadership positions). Data were abstracted using a standardized data collection form, and best practices were summarized.**Results**: Search terms resulted in 1,842 articles for title and abstract review, with 27 manuscripts meeting inclusion criteria. Two studies focused specifically on women, and four studies focused on junior faculty from racial/ethnic backgrounds underrepresented in medicine. From the initial search, few studies were designed to specifically increase diversity or capture outcomes relevant to promotion within academic medicine. Of those which did, most studies captured the impact on research productivity and career satisfaction. Traditional one-on-one mentorship, structured peer mentorship facilitated by a senior mentor, and peer mentorship in combination with one-on-one mentorship were found to be effective strategies to facilitate research productivity.**Conclusion:** Efforts are needed at the mentee, mentor, and institutional level to provide mentorship to diverse junior faculty on research competencies and career trajectory, create a sense of belonging, and connect junior faculty with institutional resources to support career success. |
| 179 | S. Koven | What is a mentor? | 2024 | N Engl J Med | **Abstract:** Having benefited from excellent mentors over the course of her career, a physician concludes that a true mentor is someone who has more imagination about you than you have about yourself. |
| 180 | A. Kalet, A.M. Libby, R. Jagsi, K. Brady, D. Chavis-Keeling, M.H. Pillinger, et al. | Mentoring underrepresented minority physician-scientists to success. | 2022 | Acad Med | **Abstract:** As the nation seeks to recruit and retain physician-scientists, gaps remain in understanding and addressing mitigatable challenges to the success of faculty from underrepresented minority (URM) backgrounds.  The Doris Duke Charitable Foundation Fund to Retain Clinical Scientists program, implemented in 2015 at 10 academic medical centers in the United States, seeks to retain physician-scientists at risk of leaving science because of periods of extraordinary family caregiving needs, hardships that URM faculty-especially those who identify as female-are more likely to experience. At the annual Fund to Retain Clinical Scientists program directors conference in 2018, program directors-21% of whom identify as URM individuals and 13% as male-addressed issues that affect URM physician-scientists in particular.  Key issues that threaten the retention of URM physician-scientists were identified through focused literature reviews; institutional environmental scans; and structured small- and large-group discussions with program directors, staff, and participants. These issues include bias and discrimination, personal wealth differential, the minority tax (i.e., service burdens placed on URM faculty who represent URM perspectives on committees and at conferences), lack of mentorship training, intersectionality and isolation, concerns about confirming stereotypes, and institutional-level factors.  The authors present recommendations for how to create an environment in which URM physician-scientists can expect equitable opportunities to thrive, as institutions demonstrate proactive allyship and remove structural barriers to success. Recommendations include providing universal training to reduce interpersonal bias and discrimination, addressing the consequences of the personal wealth gap through financial counseling and benefits, measuring the service faculty members provide to the institution as advocates for URM faculty issues and compensating them appropriately, supporting URM faculty who wish to engage in national leadership programs, and sustaining institutional policies that address structural and interpersonal barriers to inclusive excellence. |
| 181 | S. Behkam, A. Tavallaei, N. Maghbouli, M.K. Mafinejad and J.H. Ali | Students’ perception of educational environment based on Dundee Ready Education Environment Measure and the role of peer mentoring: a cross-sectional study | 2022 | BMC Med Educ | **Objective:** The curricular reform at Tehran University of Medical Sciences (TUMS), Iran, has been implemented since 2011 when peer mentoring program started. The program is believed to have a crucial role in students’ perception of the educational environment (EE). We aimed to determine how students perceive the educational environment and compared the mentees and non-mentees’ perception of EE.  **Methods:** A cross-sectional descriptive study was conducted among 190 first-year medical students enrolling at Tehran University of Medical Sciences from March to September 2019. A questionnaire was used to collect information on students’ age, gender, marital status, dormitory status, and their mentoring status including satisfaction of mentor-mentee relationship. The study also employed Dundee Ready Education Environment Measure (DREEM). The collected data were then entered and analysed using SPSS version 20. To compare the perception of EE between mentees and non-mentees, we used independent t-test.  **Results:** The mean (SD) for total DREEM score for EE was 144.1 (19.3), which signifies a more positive than negative educational environment perception. Nonetheless, the mean scores of total DREEM was not significantly different between students with or without mentors (P =0.390). The overall mean score for student perceptions of learning for mentees was 32.47 (4.5) while for those without a mentor, the score was 31.70 (4.9) (P =0.491). The items concerned with “emphasizing factual learning” and “teacher-based teaching” were rated the least. The item “having an appropriate support system” was scored significantly different between students with or without mentors (P =0.009).  **Conclusions:** Since having an appropriate support system was significantly different between groups, we suggest curriculum designers focus on the above-mentioned issue under caption for improvement during the reform programs. |
| 182 | C. Pfund, F. Sancheznieto, A. Byars-Winston, S. Zárate, S. Black, B. Birren, et al. | Evaluation of a Culturally Responsive Mentorship Education Program for the Advisers of Howard Hughes Medical Institute Gilliam Program Graduate Students | 2022 | CBE Life Sci Educ | Effective mentorship is critical to the success of trainees in research career pathways, significantly impacting their research productivity, academic and research self-efficacy, and career satisfaction. Research faculty may be unaware of or unprepared to address mentor-mentee dynamics in mentoring relationships, especially those that go beyond traditional scientific skill development. Addressing mentorship dynamics can be even more challenging for mentors from well-represented backgrounds working with mentees from historically excluded racial/ethnic groups. The Howard Hughes Medical Institute supports programmatic interventions, like the Mentorship Skills Development (MSD) course, an innovative program that aims to advance the mentorship competencies and cultural diversity awareness of mentors. Between 2015 and 2020, more than 200 faculty mentors participated in the MSD. Quantitative and qualitative data reveal significant gains in mentorship skills and cultural awareness, with mentors reporting increases in their confidence to have conversations around race and culture with their mentees. More than 85% reported actual or intended changes to their cultural responsiveness or mentorship behaviors. Importantly, behavioral changes were also observed by their mentees. These data indicate that culturally responsive mentorship education can increase knowledge and efficacy in effective mentorship practices and improve mentorship experiences of both mentors and mentees. |
| 183 | S.E.M. Hill, W.L. Ward, A. Seay and J. Buzenski | The Nature and Evolution of the Mentoring Relationship in Academic Health Centers | 2022 | J Clin Psychol Med Settings | Mentoring has a long tradition in academic health centers, and from an institutional perspective can positively impact retention, wellness, promotion success, work satisfaction, and more. On the individual level, mentorship can provide professional growth and personal satisfaction for both participants. However, mentors may struggle with how to build their mentorship skills, navigating challenges with mentees over time, or if/how/when to conclude a mentor–mentee relationship. Mentees may not understand how to find a mentor, what the nature of that relationship is, or what their role is (what characterizes a “good” mentee). As important as mentorship is, it can be challenging for both to find and maintain a high-quality mentor–mentee relationship. This article reviews the qualities that are most critical in developing a successful mentoring relationship, the longitudinal nature of this relationship, common problems that arise, and the potential rewards that exist for each person involved in the relationship. |
| 184 | S. Jan and U. Mahboob | Online mentoring: challenges and strategies | 2022 | Pak J Med Sci | **Objectives:** To gain insights into the e-mentoring experience, needs of the stakeholders (mentors and mentees) challenges and strategies to overcome the challenges.  **Methods:** Qualitative exploratory study was conducted in Islamic International Medical College, from February 2021 to July 2021. The study duration was six months. Six ‘Semi-structured interviews’ of mentors and two ‘focus group discussions’ with mentees were conducted. A purposeful sampling technique was employed to select the respondents. Data were audio taped and transcribed verbatim. After that analysis of data was done by inductive content analysis. Data were coded line by line. Open codes were combined to form categories, which were combined to form themes through abstraction.  **Results:** Data was analyzed by using Atlas.ti. After analyzing data from mentors and mentees, 21 open codes sorted into 15 categories and abstracted to from five major themes. Participants talked about the problems of online sessions like; connectivity issues, impaired interaction, nonspecific goals, unaware of MS Teams use. They suggested strategies to make these sessions more practical; like workshops for training, face to face sessions before online mode, blended approach, careful selection of mentors and mentees. All were satisfied with security and witnessed anonymity and privacy.  **Conclusion:** Online mentoring can help students feel less lonely through social contact. E-mentoring provides flexibility to those who would usually deal with discrimination to being mentored because of their gender, ethnicity, disability or geographical location. |
| 185 | L. Heffernan and E. O'Dowd | EM and Me - Near-Peer Mentoring in an Emergency Department | 2023 | Ir Med J | **Background:** Near-peer mentoring (NPM) is increasingly considered a positve means through which junior physicians can be supported. While some research has been done on the value of NPM in other settngs, there are limited studies on the benefits to Emergency Medicine (EM) doctors. Aim This study aimed to design a pilot near-peer mentoring program in an Emergency Department and to assess mentees’ experience of it.  **Methods:** Junior doctors were mentored by near peer mentors during their placement in EM and were subsequently surveyed. This was a cross-sectional, retrospective survey design and descriptive statistics were carried out on the survey data.  **Results:** 33 junior doctors were mentored by 13 mentors over a six month period. Participants’ experiences were broadly positive and highlighted that they found it useful for their professional development. Meetings were focused on the development of professional skills with a lesser emphasis on their wellbeing. Respondents reported feeling more comfortable meeting with a colleague that was closer to them in grade, rather than a senior grade, which highlights the importance of NPM. 90% of respondents agreed that the programme should be continued in the future.  **Discussion:** Near-peer mentoring has the potential to be beneficial to junior physicians in the Emergency Department |
| 186 | S. Schrempf, L. Herrigel, J. Pohlmann, J. Griewatz and M. Lammerding-Köppel | Everybody is able to reflect, or aren't they? Evaluating the development of medical professionalism via a longitudinal portfolio mentoring program from a student perspective | 2022 | GMS J Med Educ | **Introduction:** Reflective competence is fundamental for responsible medical practice and must be systematically incorporated in medical training. To promote this, a longitudinal portfolio-based mentoring program was made mandatory at the Medical Faculty of the University of Tübingen in 2013. This study examines medical students' attitudes toward professional reflection and toward the program in general to draw conclusions about conditions as well as the needs-based design of the program.  **Method:** In winter semester 2017/18, a retrospective questionnaire survey with free text fields was conducted (total sample: N=1.405; students S 1-9; response 37%; S 1-4 “Pre-clinic”: n=231; S 5-9 “Clinic”: n=241). Opinion trends of semester groups were identified through seven semi-structured interviews with semester speaker and peer tutors.  **Results:** Differences in understanding and attitudes resulted in three positions: 1=approval, 2=ambivalence, 3=rejection. All three groups included individuals from pre-clinical and clinical settings with varying levels of experience. Prior experience and hidden curriculum influenced the position. Opinion trends confirmed the feedback.  **Conclusion:** Although reflection appears in the National Competence-based Learning Objectives Catalogue for Medicine (NKLM), reflective competence is not regarded as equivalent to other study content. Motivation, commitment on the part of the mentors, and a trusting mentor-mentee relationship are effective. The flexibility of the portfolio in terms of content and methodology, as well as the curricular integration of the program are also beneficial |
| 187 | J.C. Junn, G.J. Whitman, A.P. Wasnik, M.X. Wang, M. Guelfguat, E.D. Goodman, et al. | Virtual Mentoring: A Guide to Navigating a New Age in Mentorship | 2023 | Acad Radiol | Mentorship is a fundamental part of professional and personal growth. Over time, the fabric of mentorship has been transforming from typical one-on-one mentor-mentee relationship to other types including peer, group, speed, and virtual mentoring. When the COVID-19 pandemic hit, it caused major disruptions in many facets of life and career, including mentoring. In response to the COVID-19 pandemic, live meetings were canceled, and social distancing measures were enacted at many institutions. Thus, the traditional set-up, with a face-to-face mentor and mentee interaction, was impossible. Many virtual platforms were utilized to navigate through these restrictions. In this review, we highlight challenges in mentorship during the COVID-19 pandemic and how we implemented different strategies to promote mentorship. |
| 188 | D.A. Lakhani, K.J. Swaney and J.P. Hogg | Resident Managed Peer-Mentoring Program": A Novel Way to Engage Medical Students and Radiology Residents in Collaborative Research | 2022 | Acad Radiol | **Rationale and objectives:** Engaging medical students and radiology residents in research during clinically focused training in residency can be challenging. We investigated extending a substantial degree of supervised autonomy to qualified residents to engage, mentor, and manage teams of medical students and other residents in research projects, fostering a system of laddered mentoring, referred to as "Resident Managed Peer Mentoring Program." The structure is as follows: a resident with research experience (preceptor) first identifies small-scale hypothesis driven projects which different novice learner-researchers at different levels of research background and training can undertake. The learner and preceptor then outline the learner's deliverables and set deadlines for outcomes, with regular faculty check-ins and oversight.  **Materials and methods**: This observational study assessed the outcomes of our "Resident Managed Peer Mentoring Program" beginning November 2019. Primary outcomes were numbers of peer-reviewed publications credited to individual radiology physicians. Secondary outcomes were: numbers of radiology physicians who participated in publication and academic rank-based analysis of publication numbers for attending radiologists before and after the intervention.  **Results:** Number of peer-reviewed PubMed publications increased after our intervention (47.8%, in year-1; 167.4% in year-2). Increases also occurred in the number of radiology physicians who authored publications. The effect was largest for early career physicians.  **Conclusion**: The "Resident managed peer-mentoring program" was a productive method that proved especially beneficial for students, residents, and early career physicians in our clinically focused training program. This approach may be transferable to other programs where an increase in research participation and productivity are valued. |
| 189 | H. Farid, P. Bain and G. Huang | A scoping review of peer mentoring in medicine | 2022 | Clin Teach | **Background:** While studies have demonstrated the benefits of mentoring between junior and senior faculty, the dearth of senior mentors remains a challenge. Peer mentoring arose out of scarcity by creating communities among faculty at similar stages. Although demonstrative studies abound, no synthesis of the literature exists to characterise programme structure, content and impact on faculty.  **Methods:** We conducted a scoping review of peer mentoring programmes for faculty in academic medicine. We searched MEDLINE, Embase, Web of Science and ERIC for studies of peer mentoring programmes. Two authors independently reviewed the articles and extracted data.  **Findings**: We reviewed the titles and abstracts of 1513 studies, 75 full-text articles, and selected 19 studies for our review. About half of peer mentoring programmes were department-sponsored. The overall size varied from 3 to 104 participants; most were organised into small groups and met monthly. Fifty-eight percent included a didactic curriculum. Several studies showed an increase in publications, grant funding, retention rates and promotion, in addition to increased personal satisfaction. Qualitative data demonstrated themes of collaboration and mutual support.  **Discussion:** Programme outcomes were invariably positive with respect to participant satisfaction, and additionally, some studies showed an increase in publications, grant funding, retention rates and promotion. Camaraderie emerged as a strong theme in the programmes.  **Conclusions:** This scoping review of peer mentoring programmes can guide institutions in their efforts to create similar initiatives. |
| 190 | M.K. Li, S.L. Adus and K. Weyman | “There's always something to talk about!” The unexpected benefits of going virtual in a Canadian diversity mentorship program | 2022 | Can Med Educ J | In recent years, undergraduate medical education programs have seen an increase in the diversity of their classes across race, income, gender, sexual orientation, and more as a means of better reflecting the demographics of the general patient population. However, schools must do more than simply grant entrance into medicine to members of equity-seeking groups.  First, the virtual format alleviated cognitive and administrative burdens associated with in-person mentorship meetings which have been shown to commonly place an onus on mentees to prepare for each encounter and to shape and drive the relationship. Secondly, an important mentee milestone during relationship-building is the transition from leading formal question-and-answer discussions to more organic, free flowing conversations as equals. Finally, frequent meetings within familiar spaces allowed our mentoring relationships to grow |
| 191 | C.A. Frizell, K. L. Caruthers and D. Sturges | Intentional Mentoring of Healthcare Provider Students from Underrepresented Groups in Medicine | 2023 | Med Sci Educ | Diversity and inclusion in medicine are imperative to advance care, diversify the workforce, and decrease health disparities disproportionately affecting underrepresented groups (URG), which includes individuals from marginalized communities, eg, Black/African American, Indigenous, and People of Color (BIPOC). The National Academy of Medicine (NAM, formerly the Institute of Medicine), the Health Resources and Services Administration (HRSA), and others have urged many healthcare professions to increase URG representation among its ranks, but many have fallen short in their pursuits (or lack thereof), affecting retention. The minority tax (the service demands put on URG who represent URG opinions/service on various organization levels), bias and discrimination, personal wealth disparity, a lack of mentorship training, intersectionality and isolation, worries about confirming stereotypes, and institutional-level factors are some of the problems that threaten retention of URG in various settings. Therefore, healthcare providers in clinical settings, nonclinical settings, and training programs can be more intentional in their recruitment and retention of URG via structured mentoring opportunities.  An intentional and thoughtful mentor/mentee relationship provides new and established healthcare providers a personal advocate, increased job satisfaction and productivity, and protection from the minority tax. Unfortunately, many URG do not have ample accessibility to mentors. Additionally, these URG may feel isolated and receive mentoring disparately compared to majority counterparts impacting promotion, wellness, and longevity in their roles. Innovative styles of mentoring should be considered when establishing relationships, as the classic mentoring styles may not be effective in millennial and Generation Z healthcare providers. |
| 192 | A. Aziz, W. Shadab, L. Siddique and U. Mahboob | Exploring the experiences of struggling undergraduate medical students with formal mentoring program at a private medical college in Rawalpindi | 2023 | Pak J Med Sci | **Objective:** To explore the experiences of struggling undergraduate medical students (mentees) with formal mentoring program at a private medical college in Rawalpindi.  **Methods:** A qualitative exploratory study was carried out from March to August 2019. Data was collected from a purposive sample of sixteen struggling undergraduate students. Validated interview guide was utilized to conduct semi-structured one-to-one interviews. Interviews were audio recorded and transcribed accurately. Confidentiality and anonymity were ensured to the participants due to the sensitive nature of the data. Various measures were taken to achieve trustworthiness in the study. Manual thematic analysis was performed and consensus among all authors was built regarding themes and subthemes.  **Results:** Four themes and twelve subthemes emerged from the data. Participants were satisfied with the psychosocial outcomes of the mentoring program such as emotional, moral, and psychological support, and personal and professional development. Mentees told that mentors were their best guides who shared their life experiences. Moreover, mentors provided guidance on Islam, research methods, and case-based learning. Further, mentees said that mentors provide solutions to their problems. Useful suggestions were provided by the mentees regarding betterment in the present mentoring program such as recruitment of committed staff, the need for verbal feedback from mentees about their mentors, need for career counselling and one-to-one mentoring sessions.  **Conclusions**: Majority of the mentees were satisfied with the formal mentoring program. Mentoring focuses on personal and professional development of all medical students. In addition to the useful suggestions provided by the mentees, there is a need for the addition of specific strategies to deal with students struggling with personal or professional problems. |
| 193 | N.G. Penaloza, K.E.Z. Ardines, S. Does, S.L. Washington III, M.D. Tandel, C.H. Braddock III, et al. | Someone Like Me: An Examination of the Importance of Race-Concordant Mentorship in Urology | 2023 | Urology | **Objective:** To describe differences in urology mentorship exposure for medical students across race/ethnicity and to explore how much potential mentees valued the importance of race-concordant mentorship.  **Methods:** All medical students at UCLA received a cross-sectional survey. Dependent variables were perceived quality of mentorship in urology and association between race-concordant mentorship and perceived importance of race-concordant mentorship. Mentors were self-selected by medical students. Variables were compared across race/ethnicity using descriptive statistics and multivariate analyses. Subset analyses looking at race-concordance between mentor and student was performed using stratified Cochran-Mantel-Haenszel tests. This was performed to determine if there were differences, across race/ethnicity, in rating of importance of having a race-concordant mentor.  **Results**: The likelihood of having a urologist as a mentor was similar across race/ethnicity. Under-Represented in Medicine (URiM) students were more likely to report that having a mentor of the same race/ethnicity was extremely important (Asian 9%, Black 58%, Latinx 55% and White 3%, P < .001) compared to their non-URiM peers who were more likely to rate having a race-concordant mentor as not at all important (Asian 34%, Black 5%, Latinx 8%, White 79%, P < .001). URiM students with race-concordant mentors were still more likely to rate having a mentor of the same race/ethnicity as extremely/very important (73%) compared to their non-URiM peers (9%, P = .001). URiM students with race-discordant mentors also rated importance of mentors of the same race/ethnicity as extremely/very important (67%) compared to their non-URiM peers (11%, P = .006).  **Conclusion**: URiM medical students regard race-concordant mentorship as extremely important. Interventions addressing mentor racial/ethnic concordance and those promoting culturally responsive mentorship may optimize recruitment of URiM students into urology. |
| 194 | J. K. Silver | Six Practical Strategies to Mentor and Sponsor Women in Academic Medicine | 2023 | J Med Internet Res | This article focuses on the importance of mentorship and sponsorship for women in academic medicine, including trainees and faculty, and emphasizes the need for flexible and expanded definitions. Both the benefits and potential harms associated with sponsorship are described. There are 6 actionable strategies illustrated that may be added to a multidimensional mentoring model in order to better support women in medicine. |
| 195 | J.A. Munoz, M.A. Sarmiento, Y. Alejandra Esparza, A. Shipp, A. Luisa Veloz, Y. Edith Esparza, et al. | Mentoring Medical Students: Voices from Zoom Rooms during the Pandemic | 2022 | Int J Divers Educ | Even before the COVID-19 pandemic, careers in medicine were challenging, and the academic journey could be a lonely one. The pandemic presented both challenges and opportunities in mentoring such as utilizing technology to transform formal mentorship programs into those that are beneficial for women and minorities because these programs can offer critical engagement, opportunities to hear and express multiple perspectives, expand our capacity to understand and talk about complex social issues, and meaning-making frameworks. Medical schools can utilize innovative and creative mentoring programs to support medical students as they enter the medical profession. |
| 196 | K.A. Cameron, T.J. Rodrigues and R.N. Keswani | Developing a Mentoring Network to Navigate Fellowship and Beyond: Moving From "Mentor" to "Mentors" | 2024 | Gastroenterology | The benefits of being mentored in academic medicine are well-established: mentoring can build resiliency and adaptability, increase career satisfaction and employee retention, mitigate burnout, and result in cost savings. Mentees with active mentors have better research careers, increased scholarly productivity, move more quickly through the promotion process, and possess greater self-confidence. Mentors may serve as role models, provide insight regarding career paths, assist mentees in developing their networks, and offer mentees opportunities for regional, national, or international exposure. |
| 197 | J.K. Silver and N. Gavini | The Push-Pull Mentoring Model: Ensuring the Success of Mentors and Mentees | 2023 | Med Internet Res | Mentorship is vital for professional development in academic research and clinical practice, yet it faces challenges due to a limited number of experienced mentors and a lack of protected time for mentorship that may disproportionately affect women mentors in midcareer who are doing much of this “invisible work.” The Push-Pull Mentoring Model offers a potential solution by emphasizing shared responsibility and active engagement between mentors and mentees; it fosters a flexible and collaborative approach that is mutually (though not necessarily equally) supportive of both individuals’ career goals, with mentees pushing mentors up and facilitating opportunities in their realm of influence, including but not limited to sponsorship, while mentors are simultaneously pulling them up. The Push-Pull Mentoring Model provides a promising alternative to traditional mentoring models and may help institutions address the challenges associated with limited mentorship resources. |
| 198 | M. Kuzman | Academic Mentoring for Psychiatric Trainees During the Pandemic | 2022 | Eur Psychiatry | In most academic settings, mentoring students is a part of the activities of the academic staff. Usually, associate professors, assistant professors and professors are required to mentor graduate and postgraduate students, and in some academic settings postgraduate students are required to mentor junior students. In psychiatric clinical settings, mentoring also extends to the supervision and evaluation of clinical work of postgraduate psychiatric trainees, usually for different parts of the psychiatric training. Depending on the local organisation of academic and clinical work, numbers of mentees per one mentor as well as description of activities may vary greatly across regions. Nevertheless, “mentoring” is not systematically taught and evaluated in the majority of systems but is infrequently self-taught. The situation of COVID-19 pandemic, especially in the first wave, has created a new situation which needed quick adaptations in many of the fields of the academic work, mentoring included. In this presentation, these new situations and lessons learnt will be presented and discussed from the point of the academic centre in Croatia. Disclosure No significant relationships. |
| 199 | J. Tetzlaff, G. Lomberk, H.M. Smith, H. Agrawal, D.H. Siegel and J.N. Apps | Adapting Mentoring in Times of Crisis: What We Learned from COVID-19 | 2022 | Acad Psychiatry | In this commentary, the authors consider changes within mentoring in academic medicine during the COVID-19 pandemic. They outline several facets of the mentoring process that have been affected by COVID-19 and describe ways mentoring relationships may evolve to respond to these issues. Since the beginning of COVID, several articles have been published offering guidance on how to improve mentoring techniques, such as the importance of maintaining nurturing and consistent mentor-mentee relationships, displaying transparency regarding personal struggles, maintaining active and open communication, acknowledging challenges, and engaging in reflective practices. The current paper adds to the literature by expanding on psychological perspectives in order to offer additional suggestions for managing the mentoring relationship during unprecedented times. |
| 200 | A. Sharma, H. Leeper, S. Bang, D. Molaie and A. B. Porter | The impact of mentoring on early career faculty: Assessment of a virtual mentoring program | 2022 | J Clin Oncol | **Background:** Participation in mentorship programs for early career physicians may be crucial to developing key skills and professional networks to navigate racial, ethnic and gender leadership disparities in medicine. A 6-month virtual facilitated peer mentorship program was developed and piloted through the Society for Neuro-Oncology (SNO) Women & Diversity Committee. The evaluation of the program’s feasibility to positively impact early career physicians, investigators and trainees is presented here.  **Methods:** We designed and conducted a virtual mentoring program pilot open to SNO’s multidisciplinary members in residency, fellowship, or early career phase, leveraging peer-mentoring sessions with mid-to late-career physician mentors. A curriculum with online resources was provided recommending groups meet for 6 sessions: 3 involving the mentor and 3 dedicated to peer-mentoring. Group assignments were based on time-zones and interests. Pre- and post-participation surveys assessed mentee experience. Descriptive statistics were used to assess participant demographics and survey results.  **Results:** Our call for participation was broad; all 20 mentee applicants participated in 5 groups. Mentees were 90% women and 60% were from diverse racial and ethnic backgrounds. Most were aged 31-40 (75%) and junior faculty (50%) in neuro-oncology (65%). The 5 senior mentors (3 men and 3 of diverse race and ethnic backgrounds) practiced either neuro-oncology (3), neurosurgery (1) or radiation oncology (1). The proportion who reporting having a signature lecture increased from 15% to 62% during the pilot. A large majority reported their participation was worthwhile (85%), that they would participate again (92%) and would recommend it to others (92%). Feedback themes included positive personal growth, peer support, networking and job opportunities, access to CV reviews, lack of and desire for late career female mentors, and virtual scheduling constraints. While the pilot was limited by several variables, it was timely to connect participants in Q3 of 2020 early in the COVID-19 global pandemic. The virtual meeting environment created a venue to share and discuss topics such as work-life balance, burnout, leading through change and social connection. Despite not achieving 100% professional concordance, participants found the experience worthwhile. The tools and curriculum of topics provided was implemented differently across groups, leading to varied experiences. Finally, we did not have 100% post pilot follow up despite multiple attempts limiting our complete understanding of the pilot.  **Conclusions:** This virtual mentorship pilot program proved feasible and of value in development of early career women and diverse individuals. A resource toolkit has been designed to scale and diffuse. |
| 201 | M.M. Yang, B.P. Golden, K.A. Cameron, L. Gard, J.A. Bierman, D.B. Evans, et al. | Learning through Teaching: Peer Teaching and Mentoring Experiences among Third-Year Medical Students | 2022 | Teach Learn Med | **Phenomenon:** Classroom studies of peer-led teaching and mentoring report benefits for students both as teachers and learners. Such benefits include both improved content mastery and personal and professional development. While benefits of peer-led teaching in the clinical setting have been well characterized among other health professions, less is known within undergraduate medical education. In this study, we explored medical students' perceptions and experiences relevant to peer teaching and mentoring in outpatient clinical clerkships.  **Approach:** Third-year medical students enrolled in two different longitudinal primary care clerkships, Education Centered Medical Home (ECMH) or Individual Preceptorship (IP), participated in semi-structured interviews in 2018. Students were asked to describe their peer teaching experiences during the clerkship and to reflect on their experiences serving as role models or mentors. We analyzed transcripts utilizing a two-cycle team-based inductive approach.  **Findings:** Thirty-three students completed interviews. We derived three main themes: (1) diversity of peer teaching and mentoring opportunities, (2) transitioning one's role from learner to teacher, and (3) personal and professional development. While participants from both clerkships participated in peer teaching and mentoring experiences, ECMH students described more opportunities to interact with students across all years of medical school training, noting that "getting that guidance and in turn being able to teach is a valuable experience." ECMH students further perceived the responsibility of creating a comfortable learning environment for others. Students from both clerkships reflected on 'learning through teaching,' that teaching served as a reaffirmation of the knowledge they gained, and that teaching experience contributed to their personal and professional growth.  **Insights:** Students perceived their participation in peer teaching and mentoring experiences in the clinical setting as contributing positively to personal and professional development. Students from both clerkships reflected on their teaching and mentoring opportunities as a facilitator of growth in their own teaching skills; ECMH students further described a heightened sense of self-confidence and fulfillment. These findings highlight the importance of creating learning environments that foster peer teaching and mentoring, as such opportunities may lead to further growth as a learner and as a physician. |
| 202 | L. Lynn, L. Stadtlander, D. Inman, G. Burkholder and A. Morgan | Transforming doctoral mentoring expectations and culture: An action model approach | 2023 | New Dir Teach Learn | This article examines one large online university's action model approach to transforming doctoral faculty expectations, support, and practices through internal research studies, community conversation, and planning. Research findings, progress, and recommendations are shared. |
| 203 | J.L. Moore, A. Myers and H. McConnell | Mentoring High-Impact Undergraduate Research Experiences | 2022 | Pedagogy | This article illustrates the Ten Salient Practices of Undergraduate Research Mentors with examples for English studies. The authors include both one-to-one and research-team examples, recognizing that although much English scholarship is solitary, peers and near peers play key roles in high-quality, mentored undergraduate research experiences. |
| 204 | M. Palmeri, K. Bono, A. Huang, J.R. Gunther and M.D. Mattes | Characterization of Research Mentorship During Medical School for Future Radiation Oncology Trainees | 2024 | Adv Radiat Oncol | **Purpose:** Medical student access to radiation oncology (RO) research opportunities is important for stimulating interest in the specialty. The purpose of this study was to assess the publication record during medical school of students who ultimately matched in RO, to characterize the source(s) of their RO mentorship relative to other specialties.  **Methods and materials:** We performed web-based searches to identify manuscripts published during medical school (defined as being published from January 2016 to December 2019) for all RO residents with postgraduate year 2 status in 2020 to 2021. Students with a PhD degree and international graduates were excluded. Characteristics of these publications, the student, and the primary mentor, were assessed.  **Results:** A total of 435 publications were authored by the 148 included residents. In total, 115 (78%) attended a medical school with an affiliated RO residency program. The median number of publications per student was 2 (interquartile range, 1-4), and students' median byline author position was 2 (interquartile range, 1-4). In total, 351 publications (80.7%) were on a cancer-related topic, with 234 (53.8%) published in oncology-oriented journal, and 96 (22.0%) published in RO-oriented journals. There were 294 unique mentors, with 70 mentors (24%) on 2 or more student publications. Most mentors (n = 187, 64%) shared the same institution as the student. Mentors were most commonly radiation oncologists/radiation biologists/medical physicists (n = 153, 52.6%), surgical subspecialists (n = 53, 21%), and medical oncologists (n = 18, 6.2%). Students working with primary RO mentors were more likely to publish in an oncology-oriented journal (79.1% vs 18.2%, P < .01) or RO-oriented journal (36.2% vs 2.2%, P < .01), compared with students working with non-RO mentors, respectively. A higher percentage of publications with RO mentors occurred in the last 2 years of medical school compared with the first 2 years (64.0% vs 40.9%, respectively, P < .01).  **Conclusions**: Approximately one-half of student publications among future RO residents are published in nononcology journals, and result from mentoring relationships with non-RO physicians. |
| 205 | S. De Rosa, D. Battaglini, V. Bennett, E. Rodriguez-Ruiz, A. M. S. Zaher, L. Galarza, et al. | Key steps and suggestions for a promising approach to a critical care mentoring program | 2023 | J Anesth Analg Crit Care | In critical care medicine, where there is a demanding career with a problematic work-life balance, mentoring is an important support tool to grow professionally, creating a network of support throughout the career. The mentoring process consists of evidence-based steps to guide critical care mentors and mentees and pair them with each other according to the correct selection and matching of participants. In order to focus on the active role of a young intensivist selected as a mentee at any level and to support their success in a mentoring relationship, the NEXT Committee of the European Society of Intensive Care Medicine (ESICM) developed 2012 a mentoring program.  The critical steps of the mentoring program start from establishing a policy and program objectives, passing through the selection of participants, and matching with mentors up to the definition of the personal development plan supported by checklists, worksheets, and evaluation forms. The present manuscript provides key steps and tips for a good, essential based on our experience in the ESICM NEXT-Mentoring Program so that they guide for future mentoring programs conducted by other scientific societies. In addition, we discuss common challenges and how to avoid them. |
| 206 | R.A. Jacob, P.N. Williams and A. Chisty | Twelve tips for developing and maintaining a successful peer mentoring program for junior faculty in academic medicine | 2022 | Med Teach | Effective mentorship is widely believed to be an important factor in career satisfaction and advancement. Adequate mentorship has been linked to perceived institutional support, research productivity, and protects against burnout. Despite these facts, less than half of junior faculty in academic medicine feel as if they have adequate mentorship. Given that the current landscape in academic medicine has a paucity of available mentors, both in rank and representation for true dyadic mentorship, junior faculty clinician educators may need mentorship and paths to sponsorship. The importance of peer networks has become increasingly recognized, and some institutions have begun to use peer mentoring as a means of addressing mentorship and sponsorship needs. This model can potentially circumvent some of the main barriers to mentorship by providing protected time, ameliorating power differentials, creating an environment where members have shared goals, and mitigating the need for senior faculty mentorship. The following are twelve tips to create and maintain a successful peer mentoring group for junior faculty clinician educators in academic medicine which can serve to complement the dyadic mentorship model. |
| 207 | N. R. Templeton, S. Jeong and E. Villarreal | Editorial overview: mentoring to support professional knowledge | 2022 | Mentor Tutoring: Partnersh Learn. | The authors present findings confirming various constructs of mentoring within and across disciplines. While mentoring is typically used to support career functions, there exists a more divergent approach inherent to the interrelation of social factors contributing to individual thought and behaviors. Specifically, feedback loops, the extension of opportunity, interconnectivity, and role modeling are manifest variables intertwined in effective mentoring cycles, regardless of field of study. |
| 208 | K.S. Rallis, A. Wozniak, S. Hui, A. Stammer, C. Cinar, M. Sun, et al. | Mentoring Medical Students Towards Oncology: Results from a Pilot Multi-institutional Mentorship Programme | 2022 | J Cancer Educ | **Abstract:** The mounting global cancer burden has generated an increasing demand for oncologists to join the workforce. Yet, students report limited oncology exposure in undergraduate medical curricula, while undergraduate oncology mentorships remain underutilised. We established an undergraduate oncology society–led mentorship programme aimed at medical students across several UK universities to increase medical student oncology exposure. We electronically recruited and paired oncologist mentors and medical student mentees and distributed a dedicated questionnaire (pre- and post-mentorship) to compare mentees’ self-reported cancer specialty knowledge and oncology career motivation after undertaking a 6-week mentorship. We also determined students’ interest across specialties and subspecialties and measured mentor availability via percentage programme uptake. Statistical analysis included univariate inferential tests on SPSS software. Twentynine (23.4%) of 124 oncology specialists agreed to become mentors. The mentorship was completed by 30 students across three medical schools: 16 (53.3%) Barts, 10 (33.3%) Birmingham, and 4 (13.3%) King’s; 11 (36.7%) mentored by medical oncologists, 10 (33.3%) by clinical/radiation oncologists, and 9 (30%) by surgical oncologists. The mentorship generated a statically significant increase in students’ knowledge of the multidisciplinary team and all oncology-related specialties including academia/research but not interest towards a career in oncology. Undergraduate oncology mentoring is an effective educational, networking and motivational tool for medical students. Student societies are a valuable asset in cultivating medical student oncology interest by connecting students to faculty and increasing mentor accessibility. Further research should focus on developing an optimal mentorship structure and evaluating long-term outcomes of such educational initiatives. |
| 209 | J.J. Kusner, J.J. Chen, F. Saldaña and J. Potter | Aligning Student-Faculty Mentorship Expectations and Needs to Promote Professional Identity Formation in Undergraduate Medical Education | 2022 | J Med Educ Curric Dev | **Background:** During professional identity formation, medical students integrate their newly developing professional identities with their longstanding personal identities. Longitudinal mentorship has been shown to aid students in this process. Lack of clear relationship expectations among students and faculty is a barrier to effective longitudinal mentorship relationships.  **Methods**: A cross-sectional, survey-based study collected information about experiences from both students (mentees) and faculty (mentors). Surveys focused on collecting participants' attitudes and expectations regarding ideal and actual mentorship experiences. Descriptive statistics and Fisher's exact test analyses were used to compare the responses within and between students and faculty.  **Results**: A total of 234 faculty and 181 medical students completed the survey. There were 187 faculty respondents (79.9%) who had previously mentored students. Faculty who had versus had not previously mentored students differed significantly in their responses on the importance of mentors discussing aspects of their personal lives (71.1% vs. 54.3%, respectively, p = 0.0491), a quality valued by the majority of student respondents. As students progressed through medical school, they expressed increasing needs for personal mentorship and conversations regarding work/life integration and wellness (M1: 12.2%, M2: 18.8%, M3: 29.3%, M4: 51.7%). A minority of students (27% of M3 and 14.8% of M4 students) reported meeting faculty mentors through their clinical year experiences.  **Conclusions:** Faculty mentoring chexperience may improve student-faculty value alignment, which may in turn help to address student-identified needs pertaining to personal development and professional identity formation in medical school. By contrasting student-identified mentorship expectations with those of faculty at various stages of mentorship experience, this study lays the groundwork for the formation of more effective longitudinal mentorship programs. |
| 210 | M. Khatun, P. Akter, S. Yunus, K. Alam, C. Pedersen, U. Byrskog, et al. | Challenges to implement evidence-based midwifery care in Bangladesh. An interview study with medical doctors mentoring health care providers | 2022 | Sex Reprod Healthc | **Background:** In 2013 the first midwives in Bangladesh to be educated according to international standards completed their course and were awarded a diploma. Sixty percent of their training took place in clinical placement sites. In order to achieve appropriate mentor support while in clinical practice, a mentorship programme was initiated whereby local doctors were appointed by Save the Children. The aim of this study is to describe the mentors' purpose and the actions they took to improve midwifery care at clinical placement sites. Their appointment was intended to support local Health Care Providers (HCPs) at clinical placement sites meant for educating midwifery students in evidence-based midwifery care.  **Methods:** An open-ended interview study with 14 mentors. The data was analysed using content analysis.  **Results:** The main category, the theme that emerged from the analysis was "Creating commitment". "Creating commitment" describe how the mentors; the medical doctors employed by Save the Children, "Motivate", "Educate", "Mentor", "Advocate" and "Communicate" (subcategories) to creating commitment for quality midwifery care "In the organization of care" and "In clinical care practices" (categories). As intended, they enabled HCPs, midwifery students, and newly graduated midwives to provide quality midwifery care.  **Conclusions:** Using medical doctors' status and power to support the development of a newly emerging midwifery cadre in a country where midwifery is just emerging as a profession is because midwives integrated in the health system will improve the birthing process, improve life chances for newborns, and reduce morbidity and mortality in Bangladesh. It is recommended for implementation in other similar national contexts. |
| 211 | J.A. Keating, A. Jasper, J. Musuuza, K. Templeton and N. Safdar | Supporting Midcareer Women Faculty in Academic Medicine Through Mentorship and Sponsorship | 2022 | J Contin Educ Health Prof | Midcareer women faculty face unique career challenges that may benefit from mentorship and sponsorship, yet such programs focused on the needs of this career phase are scarce in academic medicine. Many midcareer faculty require intentional and individual career planning to choose a path from the broad array of options in academic medicine. Ambiguous promotion criteria, increased workloads because of service or citizenship tasks, and a lack of sponsorship are among the barriers that inhibit midcareer faculty's growth into the high-visibility roles needed for career advancement. In addition, issues faced by women midcareer faculty members may be further exacerbated by barriers such as biases, a disproportionate share of family responsibilities, and inequities in recognition and sponsorship. These barriers contribute to slower career growth and higher attrition among women midcareer faculty and ultimately an underrepresentation of women among senior leadership in academic medicine. Here, we describe how a mentoring program involving individuals (eg, mentors, mentees, and sponsors) and departments/institutions (eg, deans and career development offices) can be used to support midcareer faculty. We also provide recommendations for building a mentoring program with complementary support from sponsors targeted toward the specific needs of women midcareer faculty. A robust midcareer mentoring program can support the career growth and engagement of individual faculty members and as a result improve the diversity of academic medicine's highest ranks. |
| 212 | A. Vieira, M.M. Cabri, S. Spijkers, A.C. Vieira and M. Maas | Mentoring in radiology: An asset worth exploring! | 2022 | Eur J Radiol | Residents experience high pressure to be successful in both their career and in keeping up an optimal work-life balance. With a mentoring program, faculties can alleviate stress and provide help for their residents. It is now well established that mentor-mentee relationships during medical school, have influence in career decisions and professional identity formation. The same can be said for mentor-mentee relationships during radiology residency. In general, universal rules of mentoring are also useful and applicable in the field of radiology. These universal rules for establishing a successful mentoring relationship include creating a relationship of trust and confidentiality, clearly defining roles and responsibilities, establishing short- and long-term goals, using open and supportive communication, and collaboratively solving problems. The institutions and the radiology departments should be well prepared and aware of the responsibility to have trainees, providing time for mentors to dedicate to their academic duties. They have to implement strategies to effective mentor matching and orientation as well as the ability to provide evaluation with qualitative feedback. Periodic assessment should be warranted together with the incorporation of new technology as it plays a critical role in the training of millennial radiologists as they take the profession into a technology-laden future of medical imaging. |
| 213 | M. K. Mishra | Evidence-based learning modules and culturally responsive mentoring to engage underserved undergraduate students | 2022 | J Immunol | The number of underrepresented minorities in the biomedical workforce has quadrupled over the past three decades, yet doctoral-level minority scientists still make up less than 5% of the total workforce. Thus, it is imperative to implement strategies to maintain the degree and diversity of scientific research and education expertise in biomedical disciplines. In this study, we develop, deploy, and assess the effectiveness of immunology learning by integrating evidence-based learning (EBL) modules to engage and motivate underserved students. Additionally, we provided robust culturally responsive mentoring to engage students in immunology education and research. Our initial finding demonstrates that EBL modules enhanced the collaborative learning and research activities and broadened their understanding of immunological problems. Therefore, our study suggests that a) implementing EBL modules in the immunology curriculum and b) engaging students through in-person mentoring during classroom engagement, attract and sustain underrepresented minority students to the immunology courses, improve undergraduate research training, and enhance educational appetite in biomedical disciplines. |
| 214 | L. Verhoef, A. Vivekanantham, A. Berti, E. Bolek, H. Smeele, M. Oztas, et al. | The Emerging Eular Network (EMEUNET) Peer-Review Mentoring: Ten Years of Initiative | 2022 | Ann Rheum Dis | **Background:** In 2012, the Emerging EULAR (European Alliance of Associations for Rheumatology) Network (EMEUNET) started a mentoring program in collaboration with the editorial board of top-leading journals in rheumatology, the Annals of the Rheumatic Diseases (ARD) and a few years later RMD Open, with the aim of improving peer reviewing skills of young researchers (mentees).1 In this program, now in its 6th edition, senior reviewers (mentors) critically discuss manuscripts submitted to ARD or RMD Open with mentees. At the end of the program, senior reviewers certify the capability of mentees to independently conduct a good quality review. The program is organized by members of the EMEUNET Peer Mentoring Subcommittee, including facilitating communication within the groups. Several strategies, such as face-to-face meetings and periodic videoconferences, were implemented recently, following the outcome of a previous survey among mentees.  **Objectives:** To assess the experienced benefits and challenges of the EMEUNET Peer Mentoring program of young rheumatologists and researchers and their mentors.  **Methods**: In November 2021, a survey was sent by email to mentors and mentees who successfully completed the first five editions of the program (launched between 2012 and 2019), asking for demographics, and potential benefits and challenges of the program. Felt change in peer-review skills before and after the program were rated on a scale from 0 (no skill) to 10 (perfect skill). Results were analysed descriptively.  **Results:** A response rate of 55% for mentors (11/20) and 43% for mentees (37/87) was obtained. Mentors had a mean(SD) age of 52(9.5) years, 64% were male and 7 different nationalities were included. Mentees had a mean age of 34(3.7), 43% were male and 16 nationalities were included. Mentees/mentors from all the editions were included, although recent editions were somewhat overrepresented. Almost all respondents said their overall experience with the program was positive (46/47), that the objectives of the peer-reviewing mentoring program were met (46/47) and that they would recommend the program to others (44/45).  Mentors indicated an initial average peer-review skill level of 5.2(1.8) for content and 4.2(1.9) for form, which improved by 2.7(1.3) points 3.2(1.8) points, respectively. Interestingly, improvement scores of the mentees paralleled those of mentors: content and form were initially rated at 4.9(1.7) and 5.1(1.8) and improved by 2.6(1.3) and 2.7(1.7), respectively. Nine out of ten mentors said the program had helped them improve their own skills (i.e., peer-reviewing, mentoring, and teaching). The number of peer reviews after completion of the program varied quite significantly between mentees (median 10, IQR 9.5-29). For most mentees, the number of peer-reviews stayed the same (18/32) or increased (12/32). Fifteen out of 32 respondents said they were invited as an independent reviewer for ARD and/or RMD Open after completion of the program. Added value mentioned by both mentors and mentees was the opportunity to contribute to high quality peer-review standards and improve their skills. Challenges reported by mentors were communication with mentees, stringent deadlines, and the program being time-consuming; challenges for mentees were the communication with their mentor, deadlines and insufficient clarity of the process.  **Conclusion:** After 5 editions over 10 years, the EMEUNET Peer-Review Mentoring Program continues to be a highly valued opportunity in the field of rheumatic diseases, as both mentors and mentees experience a significant impact on their skills. Areas for improvement were identified (e.g., communication, deadlines) and will be addressed in future editions. |
| 215 | H. Moawad | Best Practices for Mentoring New Physicians | 2022 | Oncology | Established physicians often mentor younger physicians within the same field. Recently, formal mentoring has increased, with systemized processes becoming more and more prevalent. When considering a mentorship, physicians should clarify what guidance they will provide, communicate their availability, and provide transparency about the formality, or informality, of the process. |
| 216 | C. Hoffmeyer, A. Milliren and D. Eckstein | The Hoffmeyer Mentoring Activity Checklist: Invitations to Professional Growth | 2022 | J Invitational Theory Pract | In this article, invitational theory is applied to the process of mentoring. The results of three research studies exploring mentoring of first-year school teachers led to factor analysis research in which 22 specific mentor related activities and behaviors in assisting first-year teachers are identified. These results were compiled to create the Hoffmeyer Mentoring Activity Checklist (HMAC). |
